# Supplementary material for: A high-quality chromosome-level genome assembly of the oligophagous fruit fly Bactrocera tsuneonis (Diptera: Tephritidae) and insights into its host specificity
Source: Gigascience. 2025 Nov 20;14:giaf143. doi: 10.1093/gigascience/giaf143 (PMC12723664; doi:10.1093/gigascience/giaf143)
Supplement: giaf143_Supplemental_File [file giaf143_supplemental_file.docx]

**Supplemental Information**


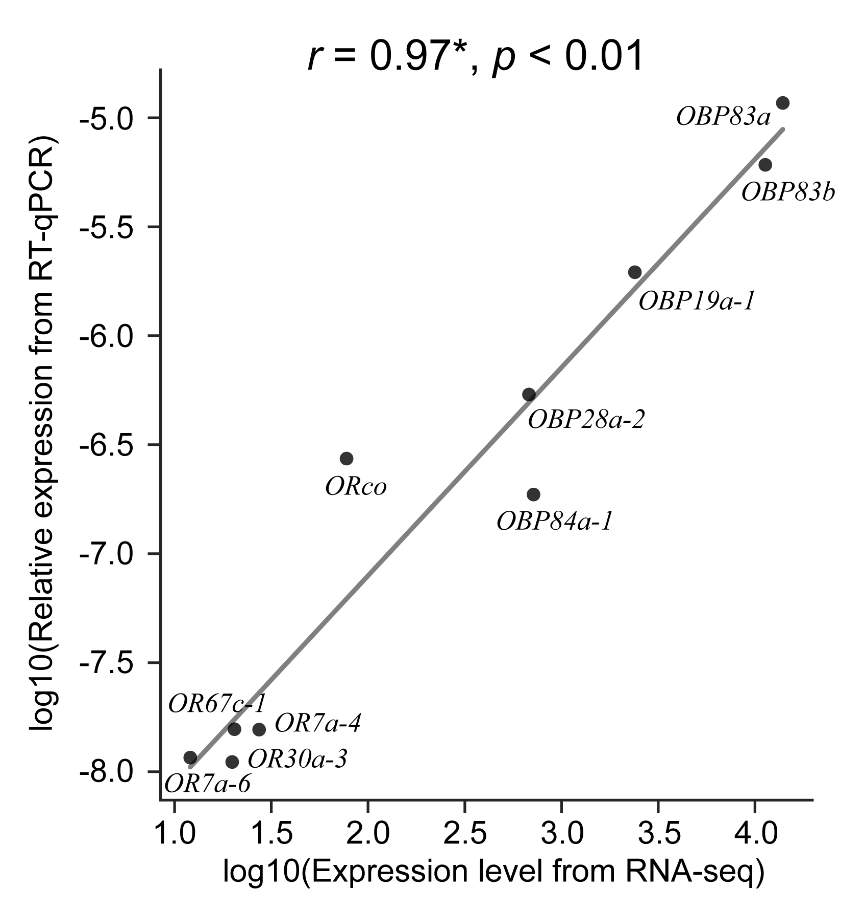


**Figure S1:** Correlation between gene expression levels from RNA-seq and RT-qPCR


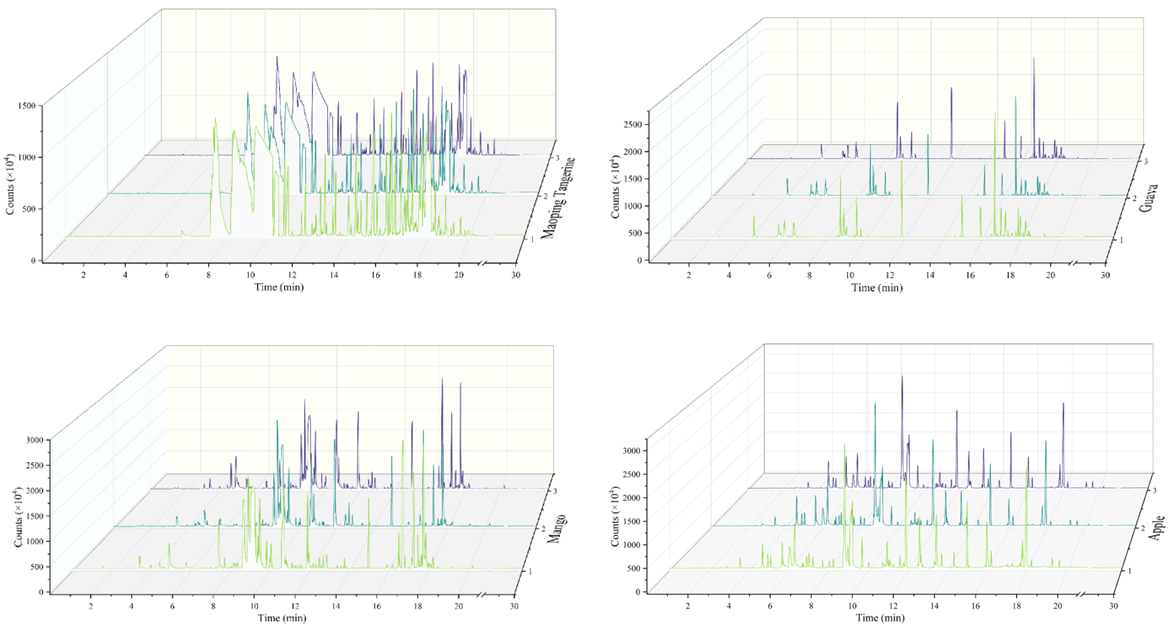


**Figure S2:** Gas chromatograph-mass spectrometry analyses (GC-MS) of host fruits (guava, mango, and apple from the previous study).


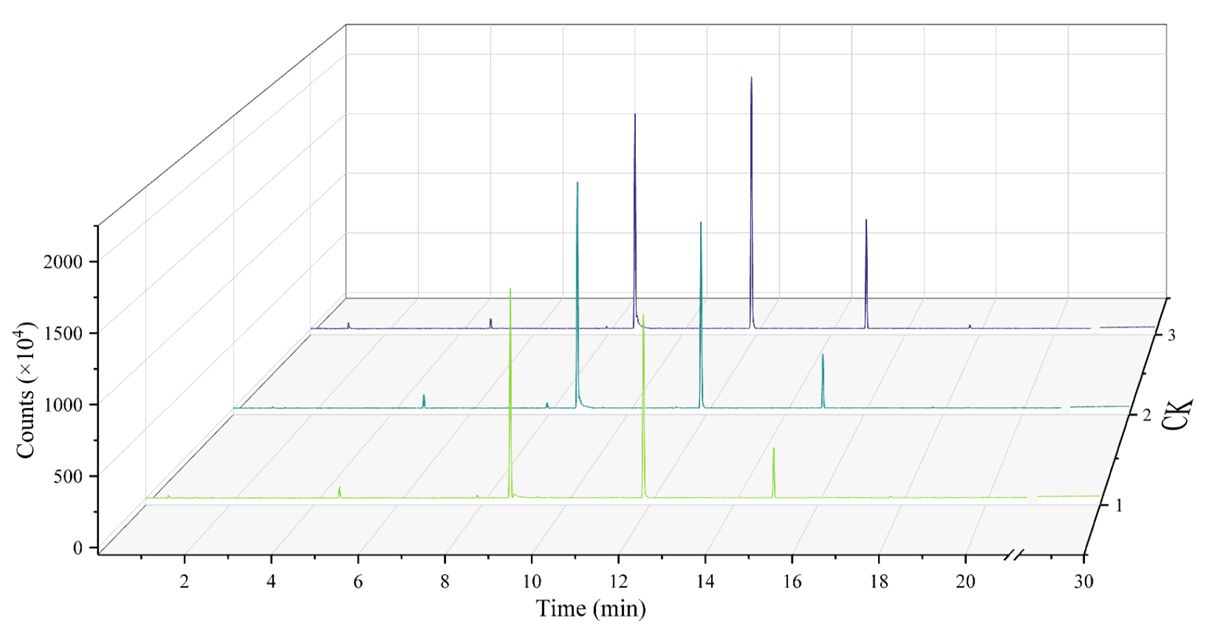


**Figure S3:** Gas chromatograph-mass spectrometry (GC-MS) analyses without fruit.


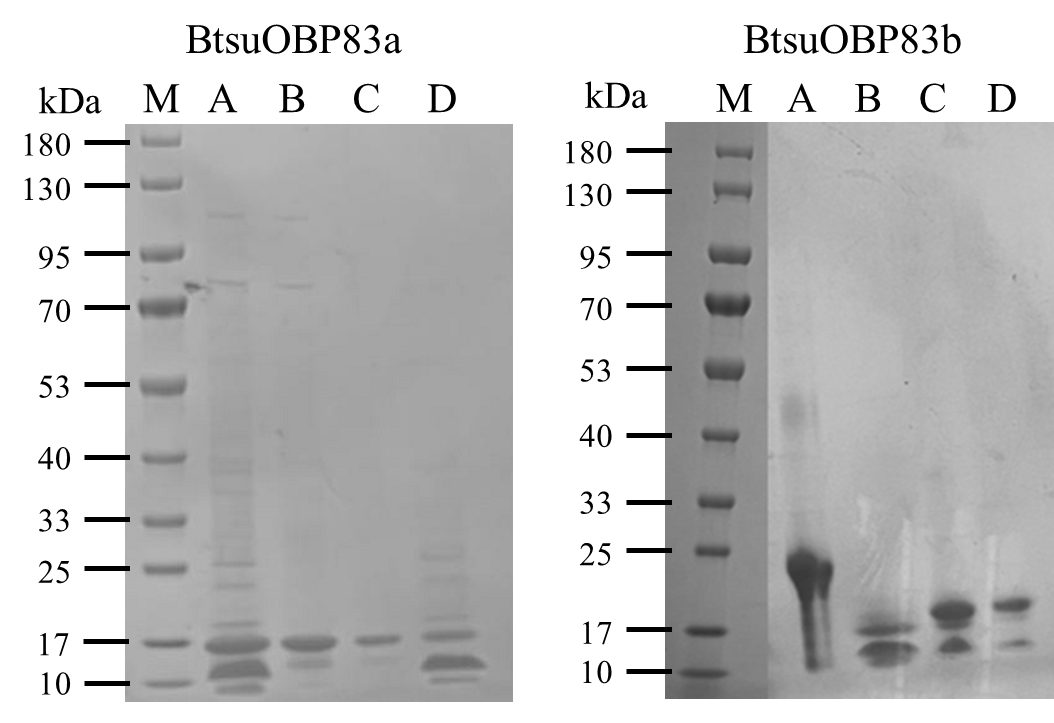


**Figure S4:** SDS-PAGE analysis of recombinant target proteins. (M: Molecular Weight Marker; A: Protein Sample After Enterokinase Cleavage; B: Target Protein After Enterokinase Cleavage; C: PBS Elution Fraction; D: 500 mM Imidazole Elution Fraction).


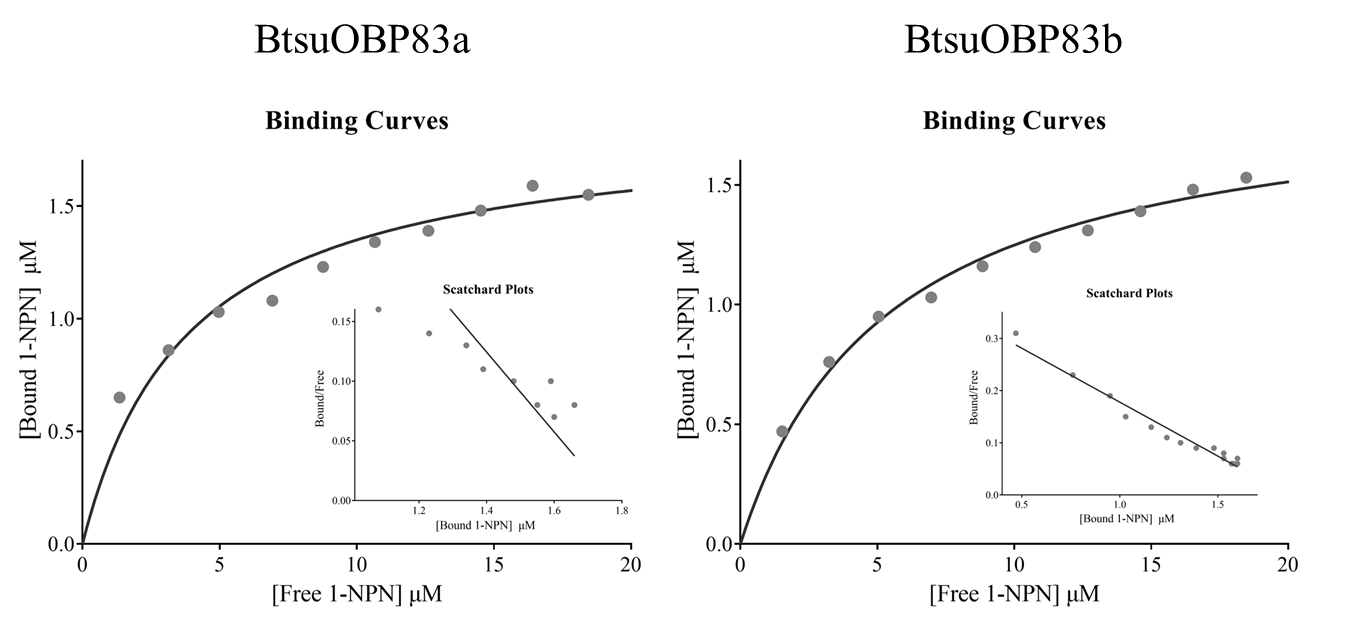


**Figure S5:** Binding curves and Scatchard plots of the fluorescence probe 1-NPN to BtsuOBPs.


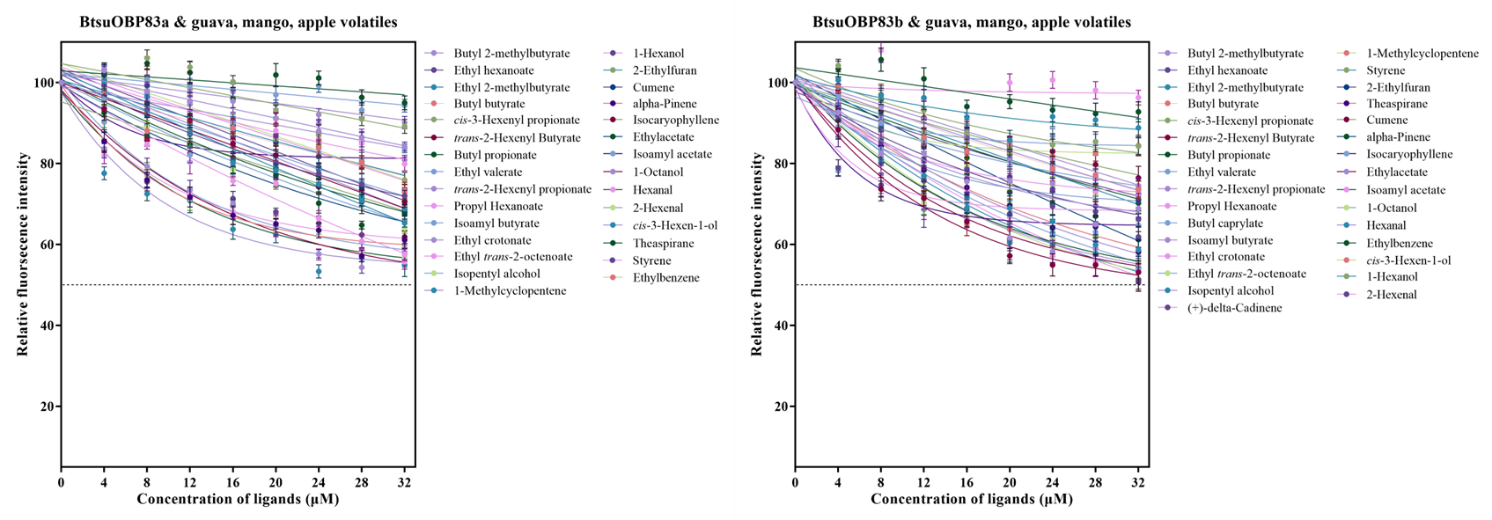


**Figure S6:** Comparison of binding properties of (A) BtsuOBP83a and (B) BtsuOBP83b with respect to guava, mango, apple.


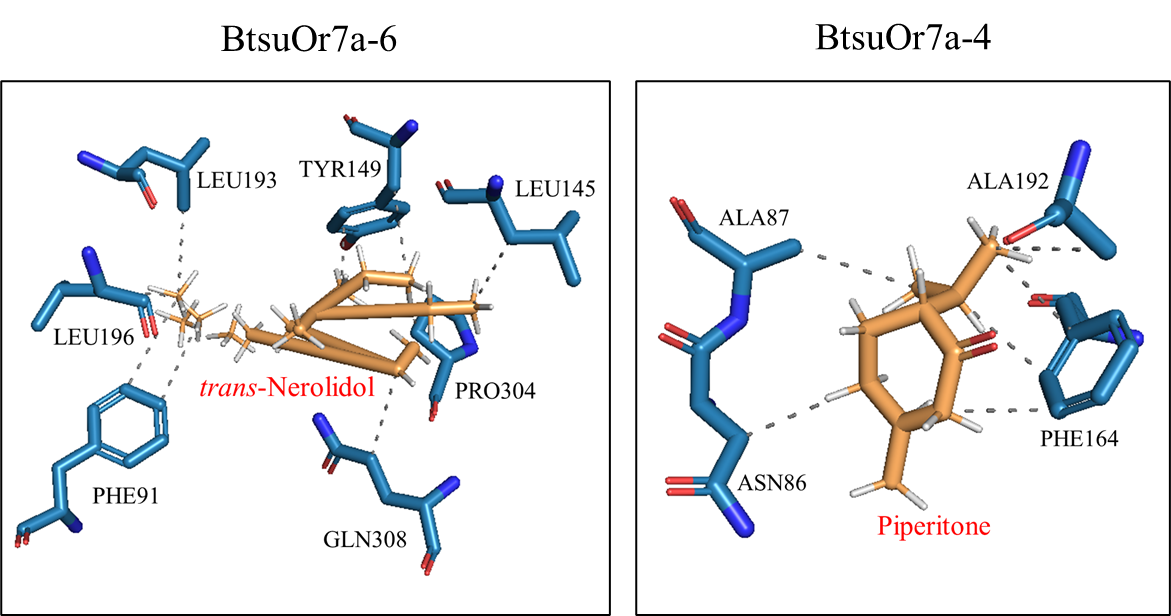


**Figure S7:** Key residues of BtsuOrs with respect to *trans*-nerolidol and piperitone.


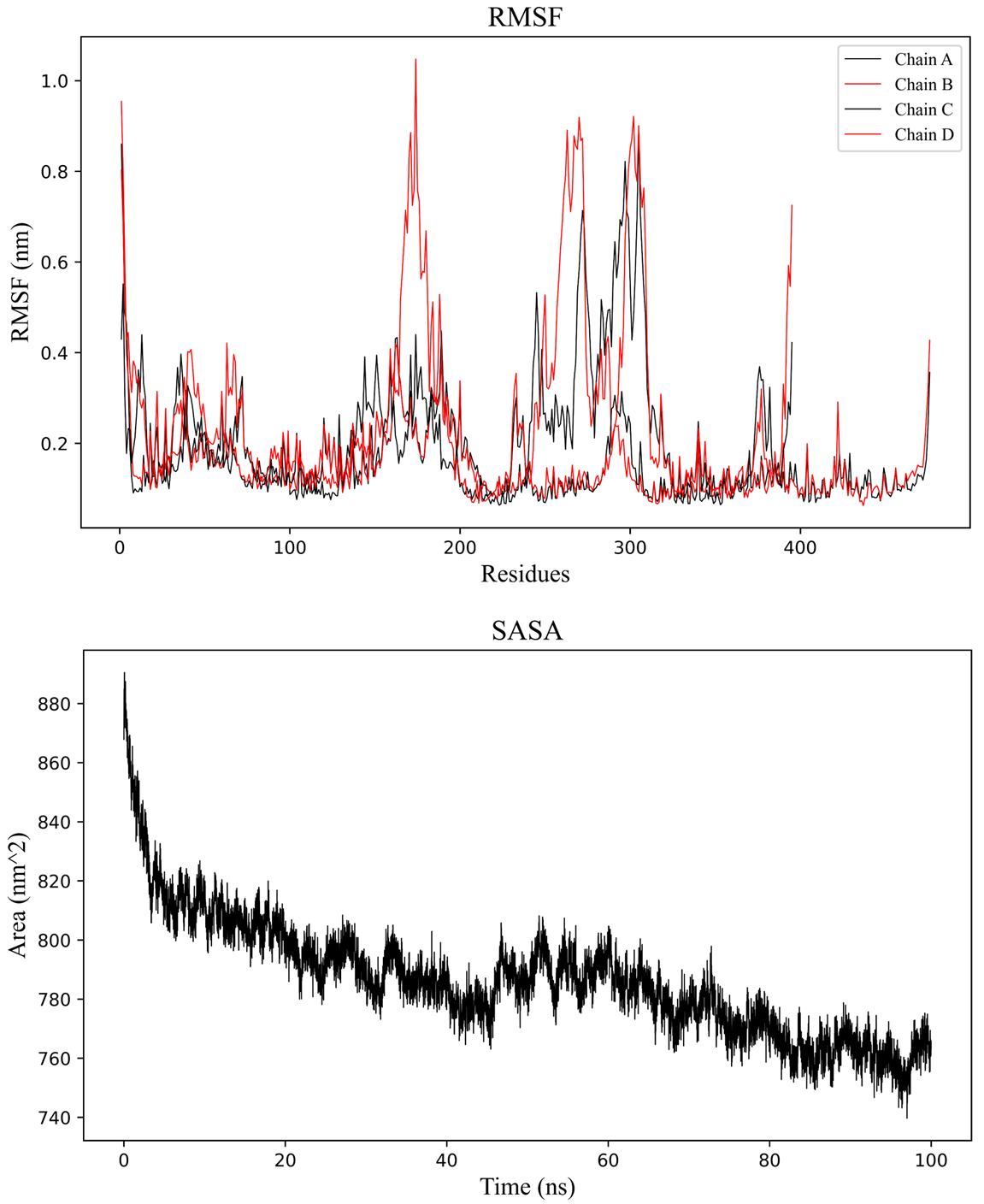


**Figure S8:** RMSF and SASA analysis of BtsuOr7a-6 in complex with *trans*-nerolidol.


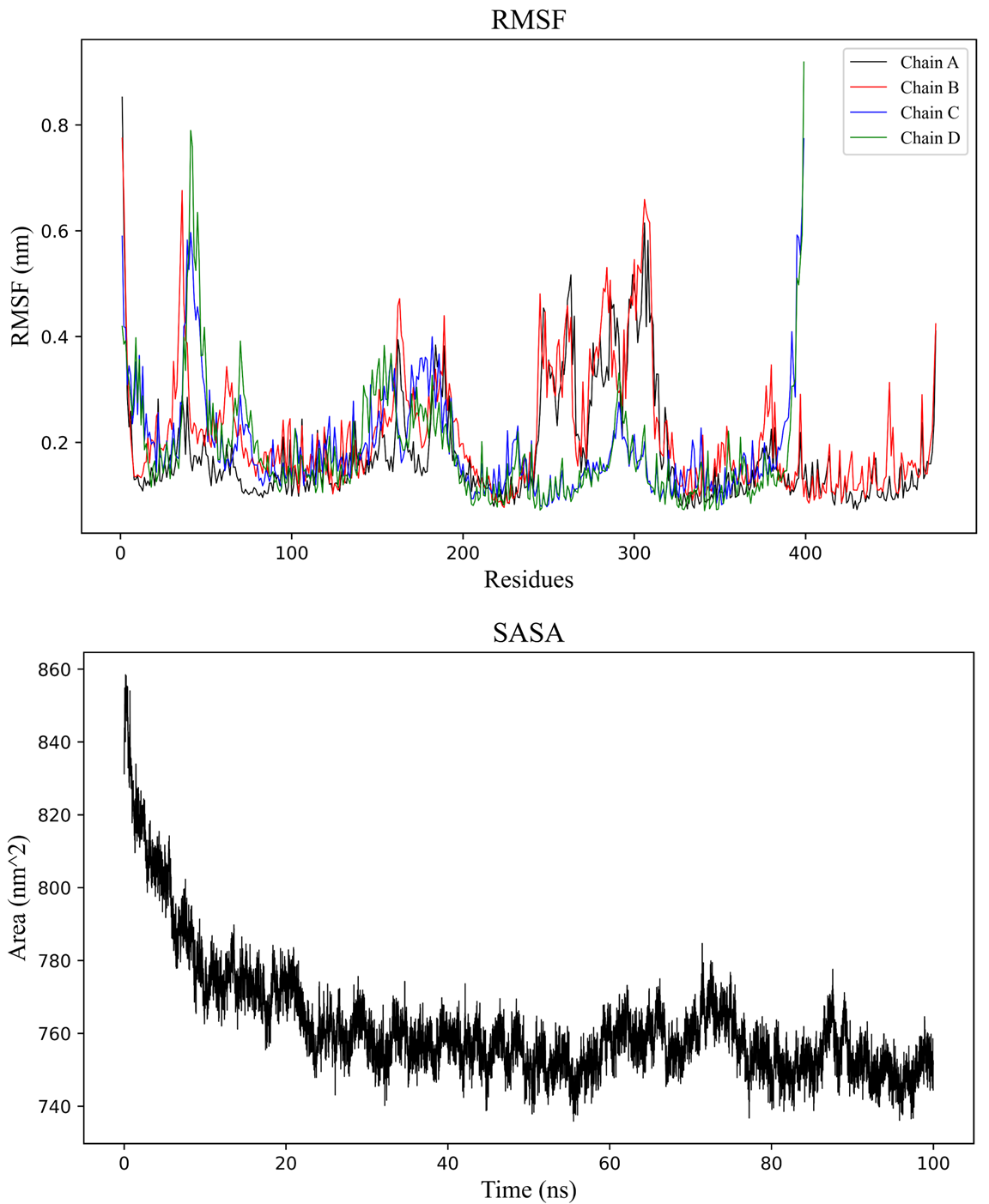


**Figure S9:** RMSF and SASA analysis of BtsuOr7a-4 in complex with piperitone.

**Table S1:** Primers used in this study.

| Name | Formard (5’-3’) | Reverse (5’-3’) |
| --- | --- | --- |
| Primers for vector construction | | |
| BtsuOBP83a | TCATCGCTGACTCTGATGCA | CCCTGAAAGCTCTATGGTAGGAA |
| BtsuOBP83a  (with restriction enzymes) | CGCGGATCCTCATCGCTGACTCTGATGCA | AAGGAAAAAAGCGGCCGCCCCTGAAAGCTCTATGGTAGGAA |
| BtsuOBP83b | ATGAAACCTATCCACCCTCC | GAGTGACTCACATTTAGATCAAG |
| BtsuOBP83b  (with restriction enzymes) | GAGAATTCATGAAACCTATCCACCCTCC | AAGGAAAAAAGCGGCCGCGAGTGACTCACATTTAGATCAAG |
| Primers for RT-qPCR | | |
| BtsuOBP83a | CGATGTAGTGGACGATAACGGTGAT | TCGGCCTTCTTCCAGCATTGATG |
| BtsuOBP83b | GGGTACACTGTTGTGGTTCGGTTT | TACATCGCCATCGCTGAACTCCT |
| BtsuOBP19a-1 | ACTGCTGTATTCATCGCCTTGGTT | CCATCTGCCACTTCTGCTGATACC |
| BtsuOBP84a-1 | TTGCTCTCTCACTGCTTCTGTTTGG | TGGTCGTCTCCGTGGACTGTTG |
| BtsuOBP28a-2 | CGAACCTGCTGATGACGCCTTC | GACCTTCCATTGTACCGTACCGATT |
| BtsuORco | AGCCTATTCGTGCCACTGGTATGA | GACTGTGAAGAACTTCGCTCCTGAT |
| BtsuOR7a-4 | TACAGCGTGCTCAGGTGGAAGT | TTCTGCCCAAAGCCCATCTCATTC |
| BtsuOR67c-1 | GCTGATATGAAAGTGCTGGGACCAT | AACAAGTGCCGACGTAGTTGAGAAG |
| BtsuOR30a-3 | TGCCACTTACGCTTCCTTCATACG | AATGCTTCCAGCTCGGTCATATCG |
| BtsuOR7a-6 | AGCCTCTTGACGGATCGTGTGA | GCAGACGCAGATAATAGCCGCATA |
| BtsuTub | CTGAACGCTGACTTACGC | GAGATAACGTCCGTGTCG |
| BtsuUBQ | TGATGCGTCCACGAATAG | CGTTCAATATCGTCCACA |

**Table S2:** Restriction enzymes used in this study.

| OBP protein | 5’ | 3’ |
| --- | --- | --- |
| BtsuObp83a | EcoR Ⅰ | Not Ⅰ |
| BtsuObp83b | BamH Ⅰ | Not Ⅰ |

**Table S3:** Statistics of sequencing data of *Bactrocera tsuneonis* genome.

| Library | type Usage | Insert Size (bp) | Clean Data (Gb) | Coverage (X) |
| --- | --- | --- | --- | --- |
| Illumina | Genome survey | 350 | 24.91 | 71.45 |
| PacBio | Genome assembly | 15,404 | 37.82 | 108.44 |
| Hi-C | Hi-C assembly | 400 | 58.88 | 168.85 |

**Table S4:** Completeness of *Bactrocera tsuneonis* genome assembly and annotation evaluated by BUSCO based on insecta_odb10 database

| Source | Complete  (C) | Single  copy (S) | Duplicated  (D) | Fragmented  (F) | Missing  (M) |
| --- | --- | --- | --- | --- | --- |
| Contig-level | 99.5 | 96.6 | 2.9 | 0.4 | 0.1 |
| Chromosome-level | 99.1 | 98.0 | 1.1 | 0.4 | 0.5 |
| Annotation | 99.1 | 85.6 | 13.5 | 0.2 | 0.7 |

**Table S5:** Statistics for repeat elements in the genome of *Bactrocera tsuneonis*

| Types | Number | Length (bp) | Percentage (%) |
| --- | --- | --- | --- |
| Retroelements | 36582 | 11693589 | 3.45 |
| SINEs | 0 | 0 | 0 |
| Penelope | 0 | 0 | 0 |
| LINEs | 25559 | 6164194 | 1.82 |
| CRE/SLACS | 0 | 0 | 0 |
| L2/CR1/Rex | 7677 | 1473054 | 0.43 |
| R1/LOA/Jockey | 5829 | 1921209 | 0.57 |
| R2/R4/NeSL | 23 | 22878 | 0.01 |
| RTE/Bov-B | 2312 | 418395 | 0.12 |
| L1/CIN4 | 60 | 3986 | 0 |
| LTR elements | 11023 | 5529395 | 1.63 |
| BEL/Pao | 1564 | 1219121 | 0.36 |
| Ty1/Copia | 0 | 0 | 0 |
| Gypsy/DIRS1 | 8893 | 4163429 | 1.23 |
| Retroviral | 175 | 39346 | 0.01 |
| DNA transposons | 67129 | 15698410 | 4.63 |
| hobo-Activator | 4309 | 734339 | 0.22 |
| Tc1-IS630-Pogo | 51440 | 13034722 | 3.84 |
| En-Spm | 0 | 0 | 0 |
| MuDR-IS905 | 0 | 0 | 0 |
| PiggyBac | 1148 | 169572 | 0.05 |
| Tourist/Harbinger | 95 | 37813 | 0.01 |
| Other (Mirage, P-element, Transib) | 198 | 67508 | 0.02 |
| Rolling-circles | 34326 | 9972397 | 2.94 |
| Unclassified | 150290 | 33734240 | 9.95 |
| Total interspersed repeats |  | 61126239 | 18.03 |
| Small RNA | 238 | 182322 | 0.05 |
| Satellites | 865 | 56911 | 0.02 |
| Simple repeats | 203409 | 9232637 | 2.72 |
| Low complexity | 25943 | 1375047 | 0.41 |

**Table S6:** Functional annotation statistics of *Bactrocera tsuneonis* genome

| Annotation type | Genes number | Percent (%) |
| --- | --- | --- |
| NR | 12,969 | 89.26 |
| Swiss-Prot | 8,762 | 60.31 |
| GO | 9,073 | 62.45 |
| KEGG | 7,539 | 51.89 |
| COG | 10,748 | 73.98 |
| Interproscan | 12673 | 87.23 |
| Eggnog | 11,697 | 80.51 |
| Pfam | 10483 | 72.15 |
| Total annotated genes | 13,513 | 93.01 |

**Table S7:** Information regarding gene family clustering in the 17 species used for comparative analyses.

| Species | *Afra* | *Alud* | *Aobl* | *Bcor* | *Bdor* | *Blat* | *Bmin* | *Bole* | *Btry* | ***Btsu*** | *Ccap* | *Dmel* | *Nasi* | *Rpom* | *Rzep* | *Zcuc* | *Ztau* |
| --- | --- | --- | --- | --- | --- | --- | --- | --- | --- | --- | --- | --- | --- | --- | --- | --- | --- |
| Number of genes | 45004 | 50460 | 51382 | 21015 | 14607 | 12490 | 35655 | 20703 | 13817 | **14529** | 12222 | 13955 | 21271 | 21503 | 25206 | 12617 | 28803 |
| Number of genes in orthogroups | 41494 | 40564 | 40986 | 18538 | 14563 | 12418 | 27804 | 16066 | 13778 | **14293** | 12105 | 12546 | 20481 | 21072 | 22056 | 12540 | 25992 |
| Number of unassigned genes | 3510 | 9896 | 10396 | 2477 | 44 | 72 | 7851 | 4637 | 39 | **236** | 117 | 1409 | 790 | 431 | 3150 | 77 | 2811 |
| Percentage of genes in orthogroups (%) | 92.2 | 80.4 | 79.8 | 88.2 | 99.7 | 99.4 | 78.0 | 77.6 | 99.7 | **98.4** | 99.0 | 89.9 | 96.3 | 98.0 | 87.5 | 99.4 | 90.2 |
| Percentage of unassigned genes (%) | 7.8 | 19.6 | 20.2 | 11.8 | 0.3 | 0.6 | 22.0 | 22.4 | 0.3 | **1.6** | 1.0 | 10.1 | 3.7 | 2.0 | 12.5 | 0.6 | 9.8 |
| Number of orthogroups containing species | 17968 | 19565 | 20010 | 12914 | 11386 | 11069 | 16320 | 13606 | 11272 | **12543** | 10910 | 10181 | 12617 | 12178 | 13118 | 11073 | 14369 |
| Percentage of orthogroups containing species (%) | 54.4 | 59.3 | 60.6 | 39.1 | 34.5 | 33.5 | 49.4 | 41.2 | 34.1 | **38.0** | 33.1 | 30.8 | 38.2 | 36.9 | 39.7 | 33.5 | 43.5 |
| Number of species-specific orthogroups | 769 | 1030 | 1212 | 152 | 9 | 4 | 1975 | 161 | 3 | **8** | 12 | 241 | 189 | 186 | 465 | 1 | 251 |
| Number of genes in species-specific orthogroups | 2947 | 4307 | 3963 | 567 | 33 | 8 | 8501 | 426 | 10 | **23** | 43 | 893 | 904 | 542 | 1744 | 2 | 958 |
| Percentage of genes in species-specific orthogroups (%) | 6.5 | 8.5 | 7.7 | 2.7 | 0.2 | 0.1 | 23.8 | 2.1 | 0.1 | **0.2** | 0.4 | 6.4 | 4.2 | 2.5 | 6.9 | 0.0 | 3.3 |

**Table S8:** Statistics on detoxification, heatshock protein (HSP), and sensing-related genes across *Bactrocera* insects and other insects.

| Gene family | *Bactrocera* | | | | | | | *Zeugodacus* | | *Ceratitis* |
| --- | --- | --- | --- | --- | --- | --- | --- | --- | --- | --- |
|  | ***Btsu*** | *Bmin* | *Bcor* | *Bdor* | *Blat* | *Btry* | *Bole* | *Zcuc* | *Ztau* | *Ccap* |
| OBP | **39** | 37 | 46 | 53 | 46 | 51 | 43 | 62 | 50 | 46 |
| OR | **68** | 62 | 95 | 101 | 64 | 85 | 80 | 112 | 75 | 66 |
| IR | **79** | 71 | 85 | 74 | 62 | 65 | 76 | 139 | 80 | 75 |
| GR | **62** | 65 | 79 | 74 | 53 | 67 | 80 | 142 | 72 | 66 |
| CSP | **7** | 9 | 12 | 15 | 12 | 12 | 10 | 9 | 9 | 13 |
| SNMP | **14** | 14 | 20 | 14 | 14 | 14 | 13 | 17 | 14 | 14 |
| CCE | **7** | 9 | 12 | 15 | 12 | 12 | 10 | 9 | 9 | 13 |
| UGT | **30** | 29 | 35 | 37 | 25 | 26 | 35 | 46 | 32 | 26 |
| GST | **40** | 106 | 37 | 43 | 37 | 36 | 36 | 40 | 38 | 38 |
| ABC | **65** | 335 | 77 | 54 | 53 | 52 | 50 | 65 | 53 | 49 |
| CYP450 | **119** | 103 | 123 | 122 | 104 | 115 | 103 | 137 | 111 | 103 |
| Hsp20 | **19** | 24 | 12 | 16 | 15 | 15 | 20 | 16 | 12 | 14 |
| Hsp40 | **41** | 45 | 36 | 36 | 36 | 33 | 34 | 41 | 37 | 35 |
| Hsp60 | **10** | 16 | 13 | 10 | 10 | 11 | 13 | 11 | 9 | 10 |
| Hsp70 | **18** | 28 | 14 | 18 | 12 | 13 | 6 | 16 | 12 | 11 |
| Hsp90 | **3** | 6 | 3 | 4 | 3 | 3 | 3 | 3 | 4 | 3 |

**Table S9:** GC-MS Analysis of VOCs in *Maoping Tangerine*.

| Component Area | Compound Name | CAS# |
| --- | --- | --- |
| 234341571.3 | 3-Carene | 13466-78-9 |
| 133602437.7 | Santolina triene | 2153-66-4 |
| 63499312.45 | Cyclohexene, 4-ethenyl-4-methyl-3-(1-methylethenyl)-1-(1-methylethyl)-, (3R-trans)- | 20307-84-0 |
| 49080619.44 | Germacrene D | 23986-74-5 |
| 45837603.81 | (+)-4-Carene | 29050-33-7 |
| 42383580.07 | Phenol, 2-methyl-5-(1-methylethyl)- | 499-75-2 |
| 41144763.21 | Thymol | 89-83-8 |
| 33182057.41 | Cyclohexane, 1-ethenyl-1-methyl-2,4-bis(1-methylethenyl)-, [1S-(1.alpha.,2.beta.,4.beta.)]- | 515-13-9 |
| 26940217.74 | Copaene | 3856-25-5 |
| 25727323.5 | Succinic acid, 3-chlorophenyl 4-methoxybenzyl ester | 1000389-69-3 |
| 25103135.16 | 2-[(Trimethylsilyl)oxy]-2-{4-[(trimethylsilyl)oxy]phenyl}ethanamine | 1000408-18-1 |
| 24973913.17 | 1,10-Decanediol | 112-47-0 |
| 21659969.44 | Benzene, 2-methoxy-4-methyl-1-(1-methylethyl)- | 1076-56-8 |
| 21538201.79 | 1,4,7,-Cycloundecatriene, 1,5,9,9-tetramethyl-, Z,Z,Z- | 1000062-61-9 |
| 18628245.07 | .gamma.-Muurolene | 30021-74-0 |
| 18523713.16 | 2,4,6-Octatriene, 2,6-dimethyl-, (E,Z)- | 7216-56-0 |
| 17779456.06 | (R)-(-)-(Z)-14-Methyl-8-hexadecen-1-ol | 30689-78-2 |
| 17690448.12 | 1,2-Bis(4-methoxyphenyl)ethane-1,2-diamine | 51208-43-6 |
| 17507859.37 | Aromandendrene | 489-39-4 |
| 16207392.52 | cis-.alpha.-Bergamotene | 18252-46-5 |
| 16023470.34 | Naphthalene, 1,2,3,4,4a,5,6,8a-octahydro-7-methyl-4-methylene-1-(1-methylethyl)-, (1.alpha.,4a.beta.,8a.alpha.)- | 39029-41-9 |
| 14562142.23 | L-.alpha.-Terpineol | 10482-56-1 |
| 13144008.92 | 1-Decanol | 112-30-1 |
| 12866891.89 | 1-Isopropyl-4,7-dimethyl-1,2,3,5,6,8a-hexahydronaphthalene | 16729-01-4 |
| 11293604.69 | 1,13-Tetradecadien-3-one | 58879-40-6 |
| 10740558.86 | Citronellol | 106-22-9 |
| 10698325.4 | .alpha.-Cubebene | 17699-14-8 |
| 9309095.81 | 1,5-Cyclodecadiene, 1,5-dimethyl-8-(1-methylethylidene)-, (E,E)- | 15423-57-1 |
| 8271337.195 | Ethyl (E)-hex-3-enyl carbonate | 1000373-83-8 |
| 7346963.006 | .alpha.-Guaiene | 3691-12-1 |
| 5499640.861 | Nonane, 3-methyl-5-propyl- | 31081-18-2 |
| 5365788.354 | p-Mentha-1,5,8-triene | 21195-59-5 |
| 4905264.062 | D-Alanine, N-(4-butylbenzoyl)-, hexyl ester | 1000354-10-1 |
| 4776203.043 | (1S,4S,4aS)-1-Isopropyl-4,7-dimethyl-1,2,3,4,4a,5-hexahydronaphthalene | 267665-20-3 |
| 4746430.685 | Cyclohexene, 3-(1,5-dimethyl-4-hexenyl)-6-methylene-, [S-(R*,S*)]- | 20307-83-9 |
| 4718670.751 | Terpinen-4-ol | 562-74-3 |
| 4635347.182 | 1-Cyclohexene-1-carboxaldehyde, 4-(1-methylethenyl)- | 2111-75-3 |
| 4148758.809 | 2,5-Cyclohexadiene, 1,4-diethyl-1,4-dimethyl- | 1000150-21-6 |
| 4083878.237 | isoledene | 95910-36-4 |
| 3017658.37 | Benzene, (1-methoxy-4-methyl-3-pentenyl)- | 68705-86-2 |
| 2698785.422 | Benzene, 1,3,5-tris(1-methylethyl)- | 717-74-8 |
| 2434674.347 | Acetic acid, octyl ester | 112-14-1 |
| 2053965.719 | Terbutaline, N-trifluoroacetyl-O,O,o-tris(trimethylsilyl)deriv. | 325836-92-8 |
| 1888981.109 | .alpha.-Muurolene | 10208-80-7 |
| 1808674.955 | 1H-Cycloprop[e]azulen-7-ol, decahydro-1,1,7-trimethyl-4-methylene-, [1ar-(1a.alpha.,4a.alpha.,7.beta.,7a.beta.,7b.alpha.)]- | 6750-60-3 |
| 1780292.55 | 2,6-Octadien-1-ol, 3,7-dimethyl-, acetate, (Z)- | 141-12-8 |
| 1669831.876 | 1,3,5,8-Undecatetraene | 50277-31-1 |
| 1626890.888 | 1,Z-5,E-7-Dodecatriene | 83085-83-0 |
| 1607799.272 | Cubenene | 29837-12-5 |
| 1605630.124 | 1-Penten-3-one, 1-(4-methoxyphenyl)-4-methyl- | 103-13-9 |
| 1603020.364 | Isospathulenol | 88395-46-4 |
| 1574398.926 | trans-.alpha.-Bergamotene | 13474-59-4 |
| 1440756.873 | Selina-3,7(11)-diene | 6813-21-4 |
| 1410196.226 | D-Carvone | 2244-16-8 |
| 1358342.674 | 3-Cyclohexene-1-ethanol, .beta.,4-dimethyl- | 18479-68-0 |
| 1330449.831 | Heptane, 4-ethyl-2,2,6,6-tetramethyl- | 62108-31-0 |
| 1265575.947 | 4-(2-Aminoethyl)pyridine | 13258-63-4 |
| 1199058.515 | 1,6,10-Dodecatrien-3-ol, 3,7,11-trimethyl-, (E)- | 40716-66-3 |
| 1192816.853 | Benzene, 4-ethenyl-1,2-dimethyl- | 27831-13-6 |
| 1187184.13 | Bicyclo[3.1.1]hept-3-en-2-ol, 4,6,6-trimethyl-, [1S-(1.alpha.,2.beta.,5.alpha.)]- | 18881-04-4 |
| 1184549.897 | 1,2-Benzenediol, O-(4-butylbenzoyl)-O'-(isobutoxycarbonyl)- | 1000329-73-6 |
| 1155556.174 | 2,6-Octadien-1-ol, 3,7-dimethyl-, (Z)- | 106-25-2 |
| 1041372.54 | (1R,5R)-2-Methyl-5-((R)-6-methylhept-5-en-2-yl)bicyclo[3.1.0]hex-2-ene | 58319-06-5 |
| 997019.3366 | Naphthalene, 1,2,4a,5,8,8a-hexahydro-4,7-dimethyl-1-(1-methylethyl)-, (1.alpha.,4a.beta.,8a.alpha.)-(.+/-.)- | 5951-61-1 |
| 929516.8033 | Spiro[2.4]heptane, 1,5-dimethyl-6-methylene- | 62238-24-8 |
| 835531.9218 | 1,3,8-p-Menthatriene | 18368-95-1 |
| 820394.8746 | (3Z,5E)-1,3,5-Undecatriene | 19883-27-3 |
| 791831.0832 | (1R,3aS,8aS)-7-Isopropyl-1,4-dimethyl-1,2,3,3a,6,8a-hexahydroazulene | 36577-33-0 |
| 782926.8619 | (6,6-Dimethylbicyclo[3.1.1]hept-2-en-2-yl)methyl ethyl carbonate | 1000373-80-4 |
| 739555.7538 | 5-Pentylcyclohexa-1,3-diene | 56318-84-4 |
| 736287.7724 | 2-Cyclohexen-1-ol, 3-methyl-6-(1-methylethyl)- | 491-04-3 |
| 694278.422 | m-Aminophenylacetylene | 54060-30-9 |
| 682659.9722 | .alpha.-Cadinol | 481-34-5 |
| 679431.371 | (2Z,4Z,6E)-2,4,6-Undecatriene | 85615-64-1 |
| 670584.5019 | n-Pentadecanol | 629-76-5 |
| 637818.1653 | 2-Cyclohexen-1-ol, 2-methyl-5-(1-methylethenyl)-, cis- | 1197-06-4 |
| 588603.1805 | Indole | 120-72-9 |
| 524936.6884 | Octane, 2,2,6-trimethyl- | 62016-28-8 |
| 505673.8234 | Cyclohexane, 1-methyl-4-(1-methylethenyl)-, cis- | 1879-07-8 |
| 496339.0319 | 2-Cyclohexen-1-one, 3-methyl-6-(1-methylethyl)- | 89-81-6 |
| 495406.62 | cis-9-Tetradecen-1-ol | 35153-15-2 |
| 428676.7102 | 2-Pyridineethanamine | 2706-56-1 |
| 417777.0413 | 8-Heptadecene | 2579-4-6 |
| 372396.8947 | Cyclohexene, 4-(1,5-dimethyl-1,4-hexadienyl)-1-methyl- | 17627-44-0 |
| 350101.5155 | cis-(-)-1,2-Epoxy-p-menth-8-ene | 32543-51-4 |
| 321816.4253 | Butanoic acid, 3-methyl-, hexyl ester | 10032-13-0 |
| 305423.3603 | (-)-Myrtenol | 19894-97-4 |
| 302262.614 | Ethanethiol | 1975-8-1 |
| 299393.9063 | 2-Naphthalenemethanol, 1,2,3,4,4a,8a-hexahydro-.alpha.,.alpha.,4a,8-tetramethyl-, [2R-(2.alpha.,4a.alpha.,8a.alpha.)]- | 29484-47-7 |
| 291313.6828 | 4a,5-Dimethyl-3-(prop-1-en-2-yl)-1,2,3,4,4a,5,6,7-octahydronaphthalen-1-ol | 61847-19-6 |
| 288182.4521 | 1-Dodecanol | 112-53-8 |
| 279758.4426 | .beta.-Phellandrene | 555-10-2 |
| 279504.9133 | 1,3-Cyclohexadiene, 1,3,5,5-tetramethyl- | 4724-89-4 |
| 278475.6954 | 2,4-Dihydroxybenzaldehyde, 2TMS derivative | 33617-38-8 |
| 229176.454 | Bicyclo[2.2.1]hept-2-ene, 1,7,7-trimethyl- | 464-17-5 |
| 225239.9197 | (-)-Aristolene | 6831-16-9 |
| 223262.0217 | Camphor | 76-22-2 |
| 222589.6395 | 2,6,9,11-Dodecatetraenal, 2,6,10-trimethyl-, (E,E,E)- | 17909-77-2 |
| 206227.6765 | 5-Isopropyl-2-methylbicyclo[3.1.0]hexan-2-ol | 546-79-2 |
| 173189.9686 | 1,16-Hexadecanediol | 7735-42-4 |
| 165264.6476 | Benzene, 1-methoxy-4-methyl-2-(1-methylethyl)- | 31574-44-4 |
| 164100.2693 | Benzene, tert-butyl- | 1998-6-6 |
| 145075.3091 | 2-Cyclohexen-1-ol, 1-methyl-4-(1-methylethenyl)-, trans- | 7212-40-0 |
| 119618.152 | 1,3,5-Cycloheptatriene, 3,7,7-trimethyl- | 3479-89-8 |
| 109675.7518 | Cyclohexane, (1,1-dimethylethyl)- | 3178-22-1 |
| 108104.636 | (3aS,4R,7R)-1,4,9,9-Tetramethyl-5,6,7,8-tetrahydro-4H-3a,7-methanoazulene | 50430-14-3 |
| 105611.0108 | 1,1,7,7a-Tetramethyl-1a,2,6,7,7a,7b-hexahydro-1H-cyclopropa[a]naphthalene | 154098-14-3 |
| 104521.83 | (Z)-Hex-3-enyl (E)-2-methylbut-2-enoate | 1000373-73-0 |
| 103730.1523 | 2-Ethylthiolane, S,S-dioxide | 10178-59-3 |
| 91502.57155 | Pentadecanal- | 2760-11-9 |
| 90492.6111 | 1,14-Tetradecanediol | 19812-64-7 |
| 85060.75262 | Ethanone, 1-(2,4,5-trimethylphenyl)- | 2040-7-5 |
| 63647.78384 | Naphthalene, 1,6-dimethyl-4-(1-methylethyl)- | 483-78-3 |

**Table S10:** Chemical compounds used in this study.

| Chemical compounds | Host fruit | CAS | Chemical Structure Depiction | Company reagent number |
| --- | --- | --- | --- | --- |
| Carvacrol | *Maoping Tangerine* | 499-75-2 | 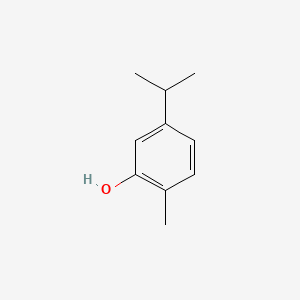 | MACKLIN–C804847 |
| 2-Isopropyl-5-methylanisole | *Maoping Tangerine* | 1076-56-8 | 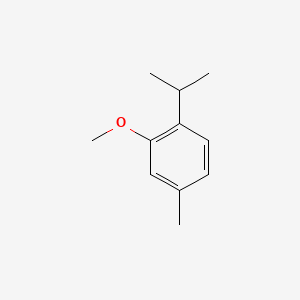 | MACKLIN–I862821 |
| (L)-alpha-terpineol | *Maoping Tangerine* | 10482-56-1 | 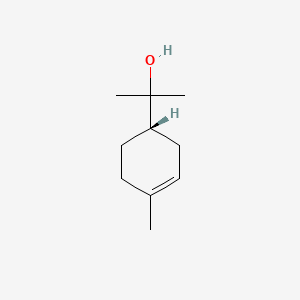 | MACKLIN–T819568 |
| Octyl acetate | *Maoping Tangerine* | 112-14-1 | 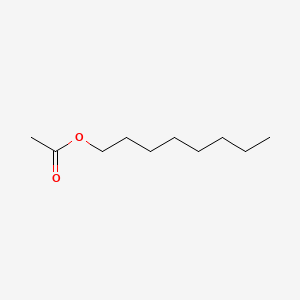 | MACKLIN–O815140 |
| Neryl acetate | *Maoping Tangerine* | 141-12-8 | 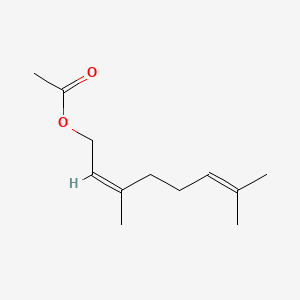 | MACKLIN–N814979 |
| d-Carvone | *Maoping Tangerine* | 2244-16-8 | 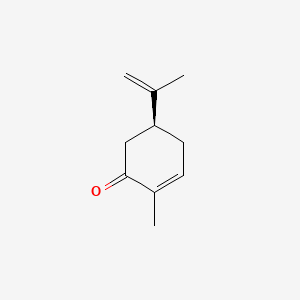 | MACKLIN–S804894 |
| *trans*-Nerolidol | *Maoping Tangerine* | 40716-66-3 | 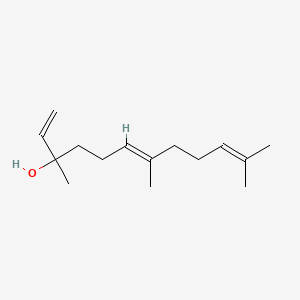 | MACKLIN–N879140 |
| Nerol | *Maoping Tangerine* | 106-25-2 | 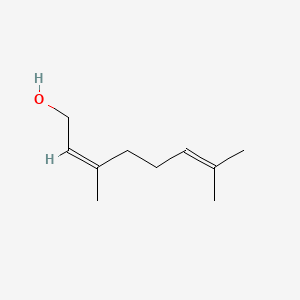 | MACKLIN–N814516 |
| 3-Ethynylaniline | *Maoping Tangerine* | 54060-30-9 | 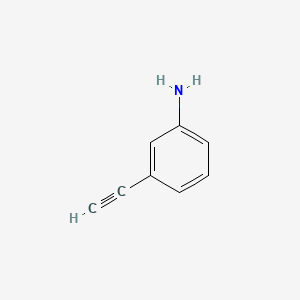 | MACKLIN–E808978 |
| Piperitone | *Maoping Tangerine* | 89-81-6 | 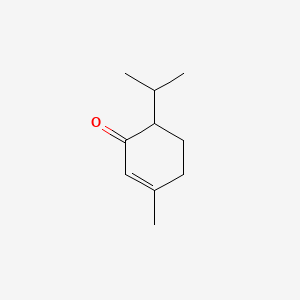 | MACKLIN–P832680 |
| Butyl 2-methylbutyrate | Apple | 15706-73-7 | 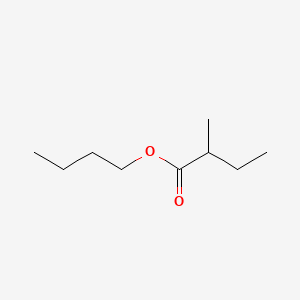 | MACKLIN–N858717 |
| Ethyl hexanoate | Guava, mango, apple | 123-66-0 | 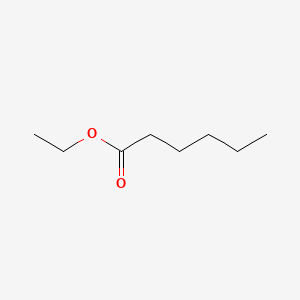 | MACKLIN–E808750 |
| Ethyl 2-methylbutyrate | Apple | 7452-79-1 | 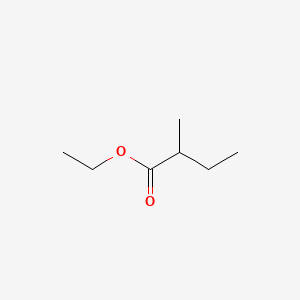 | MACKLIN–E761946 |
| Butyl butyrate | Mango, apple | 109-21-7 | 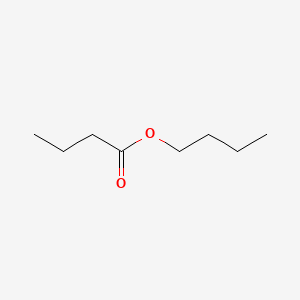 | MACKLIN–B802160 |
| *cis*-3-Hexenyl propionate | Apple | 33467-74-2 | 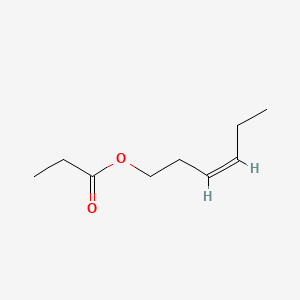 | MACKLIN–H811409 |
| *trans*-2-Hexenyl Butyrate | Apple | 53398-83-7 | 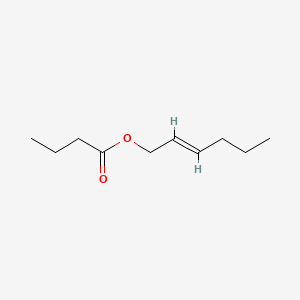 | MACKLIN–H868836 |
| Butyl propionate | Apple | 590-01-2 | 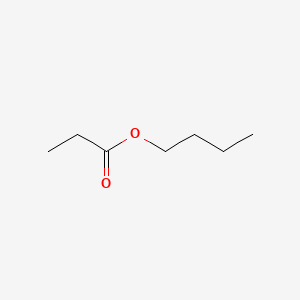 | MACKLIN–B830301 |
| Ethyl valerate | Apple | 539-82-2 | 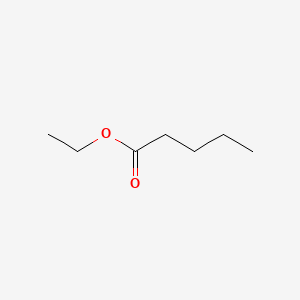 | MACKLIN–E808817 |
| *trans*-2-Hexenyl propionate | Apple | 53398-80-4 | 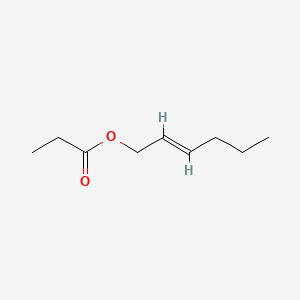 | MACKLIN–H867442 |
| Propyl Hexanoate | Apple | 626-77-7 | 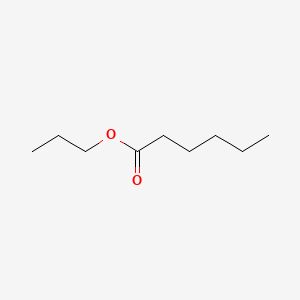 | MACKLIN–P834846 |
| Butyl caprylate | Apple | 589-75-3 | 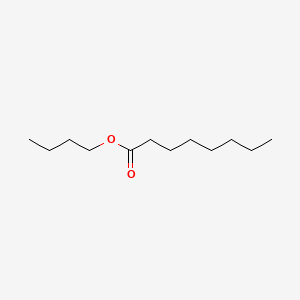 | MACKLIN–B864377 |
| Isoamyl butyrate | Apple | 106-27-4 | 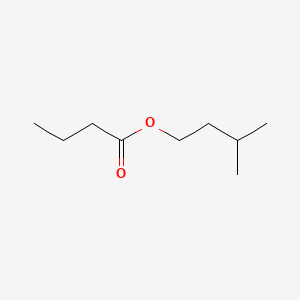 | MACKLIN–I811839 |
| Ethyl crotonate | Mango | 623-70-1 | 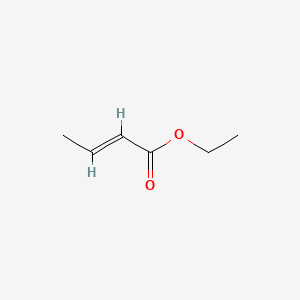 | MACKLIN–E830991 |
| Ethyl *trans*-2-octenoate | Mango | 7367-82-0 | 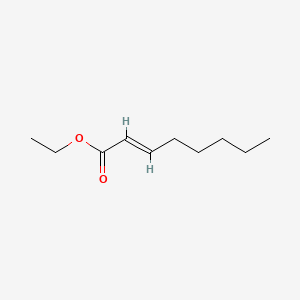 | MACKLIN–E859139 |
| Isopentyl alcohol | Guava, mango | 123-51-3 | 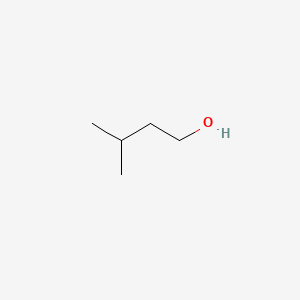 | MACKLIN–I813912 |
| (+)-delta-Cadinene | Mango | 483-76-1 | 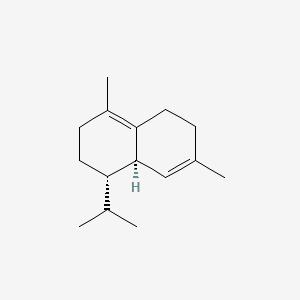 | MACKLIN–C984460 |
| 1-Methylcyclopentene | Guava | 693-89-0 | 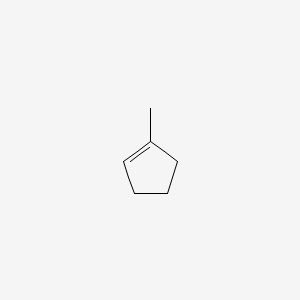 | MACKLIN–M837942 |
| Styrene | Guava | 100-42-5 | 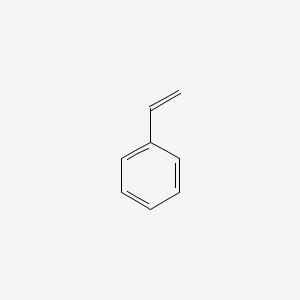 | MACKLIN–S817903 |
| Ethylbenzene | Guava | 100-41-4 | 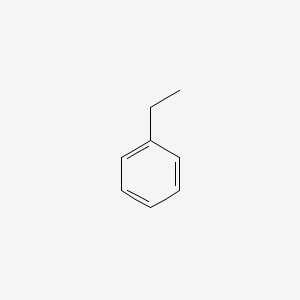 | MACKLIN–E821364 |
| 2-Ethylfuran | Guava | 3208-16-0 | 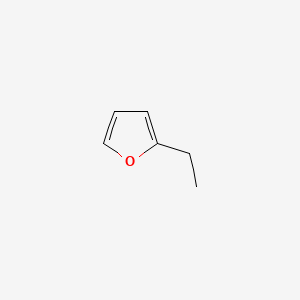 | MACKLIN–E808698 |
| Theaspirane | Guava | 36431-72-8 | 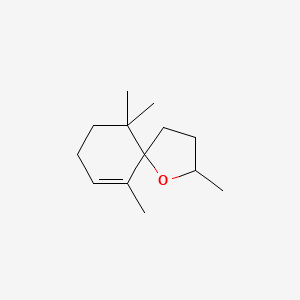 | MACKLIN–T818696 |
| Cumene | Guava | 98-82-8 | 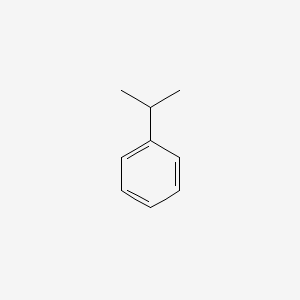 | MACKLIN–I811595 |
| alpha-Pinene | Guava | 80-56-8 | 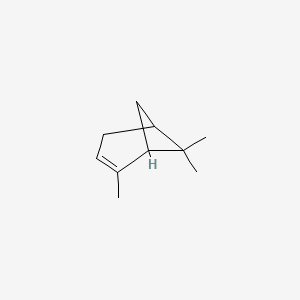 | MACKLIN–P823474 |
| Isocaryophyllene | Guava | 118-65-0 | 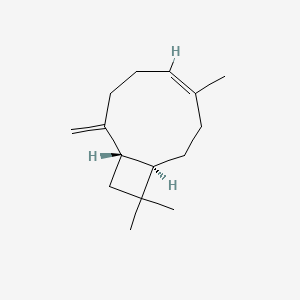 | MACKLIN–I922401 |
| Ethylacetate | Guava | 141-78-6 | 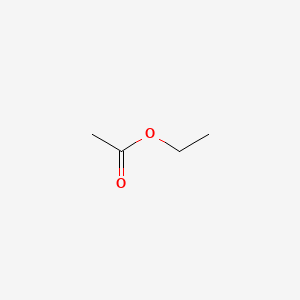 | MACKLIN–E809178 |
| Isoamyl acetate | Guava | 123-92-2 | 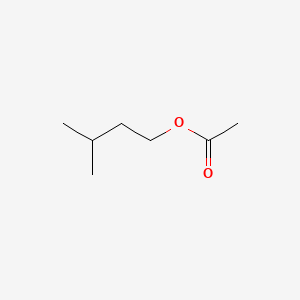 | MACKLIN–I811937 |
| 1-Octanol | Guava | 111-87-5 | 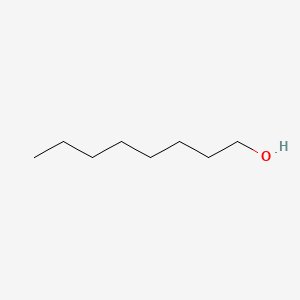 | MACKLIN–O815143 |
| Hexanal | Guava | 66-25-1 | 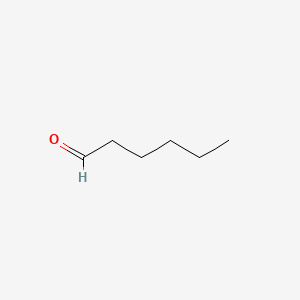 | MACKLIN–C874830 |
| 2-Hexenal | Guava | 6728-26-3 | 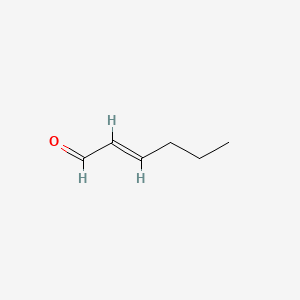 | MACKLIN–H811039 |
| *cis*-3-Hexen-1-ol | Guava | 928-96-1 | 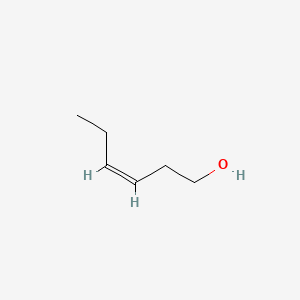 | MACKLIN–L812381 |
| 1-Hexanol | Guava | 111-27-3 | 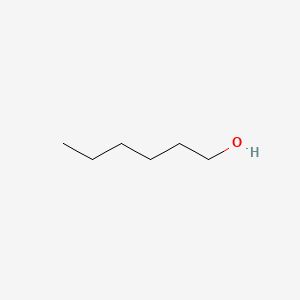 | MACKLIN–H810867 |
| 1-Fluorododecane | Apple | 334-68-9 | 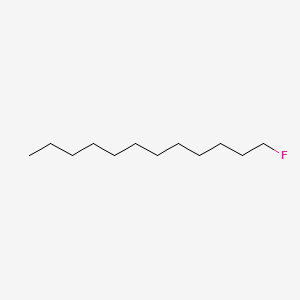 | MACKLIN–F924144 |
| beta-Caryophyllene | Guava | 87-44-5 | 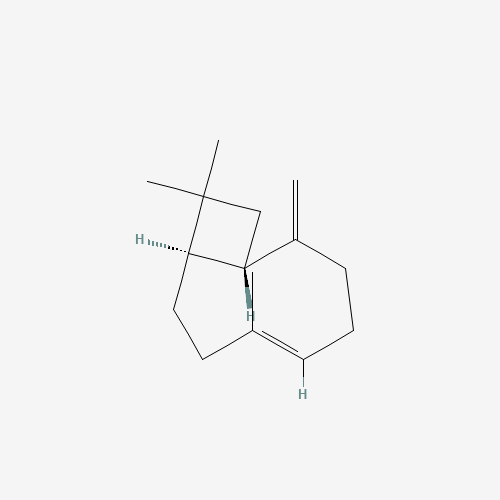 | ALADDIN–T334030 |

**Table S11:** Binding affinities of all tested ligands to BtsuOBPs.

| Ligands | Ki (μM) | |
| --- | --- | --- |
|  | OBP83a | OBP83b |
| Carvacrol | - | - |
| 2-Isopropyl-5-methylanisole | - | - |
| (L)-alpha-terpineol | - | - |
| Octyl acetate | - | - |
| Neryl acetate | - | - |
| d-Carvone | - | - |
| *trans*-Nerolidol | 9.36 | - |
| Nerol | - | - |
| 3-Ethynylaniline | - | - |
| Piperitone | 17.88 | - |
| Butyl 2-methylbutyrate | - | - |
| Ethyl hexanoate | - | - |
| Ethyl 2-methylbutyrate | - | - |
| Butyl butyrate | - | - |
| *cis*-3-Hexenyl propionate | - | - |
| *trans*-2-Hexenyl Butyrate | - | 28.48 |
| Butyl propionate | - | - |
| Ethyl valerate | - | - |
| *trans*-2-Hexenyl propionate | - | - |
| Propyl Hexanoate | - | - |
| Butyl caprylate | - | - |
| Isoamyl butyrate | - | - |
| Ethyl crotonate | - | - |
| Ethyl *trans*-2-octenoate | 46.29 | - |
| Isopentyl alcohol | - | 40.25 |
| (+)-delta-Cadinene | - | - |
| 1-Methylcyclopentene | - | - |
| Styrene | - | - |
| Ethylbenzene | - | - |
| 2-Ethylfuran | - | - |
| Theaspirane | - | - |
| Cumene | - | - |
| alpha-Pinene | 49.22 | - |
| Isocaryophyllene | - | - |
| Ethylacetate | - | 33.05 |
| Isoamyl acetate | - | 33.49 |
| 1-Octanol | 36.44 | 39.07 |
| Hexanal | 40.71 | 33.69 |
| 2-Hexenal | 42.66 | 31.92 |
| *cis*-3-Hexen-1-ol | 32.15 | 31.82 |
| 1-Hexanol | - | 31.81 |
| 1-Fluorododecane | - | - |
| beta-Caryophyllene | - | - |

Note: The Ki represents the relative dissociation constant. The BtsuOBP proteins were considered not to bind the tested ligands if the IC50 values exceeded 50 μM. In such cases, Ki values were not calculated and are represented as “–”.

**Table S12:** BtsuOrs with ligands interaction energy calculated by molecular docking.

| Mode | Affinity (kcal/mol) | |
| --- | --- | --- |
|  | *trans*-Nerolidol | Piperitone |
| BtsuOr85f | - | - |
| BtsuOr45a | - | - |
| BtsuOr35a-1 | - | - |
| BtsuOr24a | - | - |
| BtsuOr22c | - | -5.649 |
| BtsuOr94a-1 | -4.982 | -5.203 |
| BtsuOr94a-2 | - | - |
| BtsuOr30a-1 | - | - |
| BtsuOr30a-2 | - | - |
| BtsuOr33a-1 | - | - |
| BtsuOr33a-3 | - | - |
| BtsuOr33a-4 | - | -5.817 |
| BtsuOr33a-2 | - | - |
| BtsuOr42a-5 | - | - |
| BtsuOr42a-6 | - | - |
| BtsuOr2a | - | -6.223 |
| BtsuOr7a-3 | - | - |
| BtsuOr35a-2 | - | - |
| BtsuOr7a-2 | -6.113 | - |
| BtsuOr67c-2 | - | - |
| BtsuOr7a-6 | -7.381 | -6.288 |
| BtsuOr7a-7 | -5.458 | - |
| BtsuOr13a | - | - |
| BtsuOr85e | - | - |
| BtsuOr85c-1 | - | - |
| BtsuOr85c-2 | - | -4.683 |
| BtsuOr85d-1 | - | - |
| BtsuOr85d-2 | - | - |
| BtsuOr83a-1 | - | - |
| BtsuOr83a-2 | - | -4.852 |
| BtsuOr67-3 | - | - |
| BtsuOr67-4 | -7.075 | - |
| BtsuOr7a-8 | - | -5.888 |
| BtsuOr7a-1 | - | - |
| BtsuOr82a | - | -5.448 |
| BtsuOr7a-5 | - | - |
| BtsuOr10a | -6.399 | - |
| BtsuOr74a | - | - |
| BtsuOr46a | - | - |
| BtsuOr71a-1 | - | - |
| BtsuOr71a-2 | - | - |
| BtsuOr59a-2 | -5.697 | -5.724 |
| BtsuOr71a-3 | -6.563 | - |
| BtsuOr71a-4 | - | -5.013 |
| BtsuOr59a-3 | - | - |
| BtsuOr43a | - | - |
| BtsuOr88a | -5.899 | - |
| BtsuOr42a-1 | - | - |
| BtsuOr42a-2 | -5.937 | -5.211 |
| BtsuOr42a-3 | - | - |
| BtsuOr42a-4 | -5.994 | -5.128 |
| BtsuOr7a-4 | - | -6.904 |
| BtsuOr88a-2 | - | - |
| BtsuOr49b | - | - |
| BtsuOr59a-1 | -6.517 | -6.059 |
| BtsuOr47b | - | -5.881 |
| BtsuOr67c-1 | - | - |
| BtsuOr69a-1 | - | - |
| BtsuOr69a-2 | - | - |
| BtsuOr63a-3 | - | -5.817 |
| BtsuOr35a-3 | - | - |
| BtsuOr63a-1 | - | - |
| BtsuOr63a-2 | - | - |
| BtsuOr30a-3 | - | - |
| BtsuOr67d-1 | - | - |
| BtsuOr67d-2 | - | - |
| BtsuOr33b-1 | - | -5.748 |

Note: The lowest binding energy indicates the highest degree of stability between protein and ligand. If the ligand does not enter the binding pocket, the BcorOR protein is considered not to have bound to the tested ligand, represented as "–".
