# Supplementary material for: A high-quality chromosome-level genome assembly of the oligophagous fruit fly Bactrocera tsuneonis (Diptera: Tephritidae) and insights into its host specificity
Source: Gigascience. 2025 Nov 20;14:giaf143. doi: 10.1093/gigascience/giaf143 (PMC12723664; doi:10.1093/gigascience/giaf143)
Supplement: giaf143_GIGA-D-25-00162_Revision_2 [file giaf143_giga-d-25-00162_revision_2.pdf]

GigaScience

A high-quality chromosome-level genome assembly of the oligophagous fruit fly Bactrocera tsuneonis (Diptera: Tephritidae) and insights into its host specificity

--Manuscript Draft--

**Manuscript Number:**	GIGA-D-25-00162R2
**Full Title:**	A high-quality chromosome-level genome assembly of the oligophagous fruit fly Bactrocera tsuneonis (Diptera: Tephritidae) and insights into its host specificity
**Article Type:**	Research
**Funding Information:**	National Natural Science Foundation of China (32202288) — Dr. Yujia Qin<br>National Key Research and Development Program of China (2022YFC2601500) — Dr. Yujia Qin
**Abstract:**	Background<br><br>Bactrocera tsuneonis is a major pest of citrus, causing significant economic losses in fruit production. It exhibits a highly specialized host preference, primarily infesting citrus fruits. However, the genetic basis underlying its olfactory adaptation and host specificity remains largely unexplored. To elucidate the molecular mechanisms governing host selection in B. tsuneonis, we assembled a high-quality chromosome-level genome and performed comparative genomic, transcriptomic, and functional analyses of its chemosensory system.<br><br>Results<br><br>The genome of B. tsuneonis was assembled to a total size of 339 Mb, with a contig N50 of 11.21 Mb and a scaffold N50 of 59.93 Mb. Comparative genomic analysis revealed significant contractions in chemosensory-related gene families, particularly in odorant-binding proteins (OBPs) and odorant receptors (ORs), maybe suggesting an adaptation to a narrow host range. Transcriptome analysis demonstrated that BtsuOBP83a and BtsuOBP83b were highly expressed in the antennae, and most ORs were predominantly expressed in the antennae. Functional assays confirmed that BtsuOBP83a selectively binds to two citrus volatiles, trans-nerolidol and piperitone, with strong affinity. Molecular docking and molecular dynamics simulations further revealed that BtsuOr7a-6 and BtsuOr7a-4 specifically interact with these volatiles, suggesting their role in host odor recognition.<br><br>Conclusions<br><br>Our high-quality genome of B. tsuneonis provides a valuable resource for genomic research and offers valuable insights into the genetic basis of its olfactory adaptation and host specificity. The findings highlight key molecular mechanisms underlying host selection and provide potential targets for behavior-based pest management strategies.
**Corresponding Author:**	Yujia Qin<br>China Agricultural University<br>Beijing, CHINA
**Corresponding Author Secondary Information:**	
**Corresponding Author's Institution:**	China Agricultural University
**Corresponding Author's Secondary Institution:**	
**First Author:**	Tengda Guo
**First Author Secondary Information:**	

Powered by Editorial Manager® and ProduXion Manager® from Aries Systems Corporation

**Order of Authors:**	Tengda Guo
	Weisong Li
	Yuan Zhang
	Wenzhao Yang
	Zhihong Li
	Yujia Qin
**Order of Authors Secondary Information:**	
**Response to Reviewers:**	Revised version for "A high-quality chromosome-level genome assembly of the oligophagous fruit fly Bactrocera tsuneonis (Diptera: Tephritidae) and insights into its host specificity"<br><br>Dear editor,<br><br>We are honored to have the opportunity to resubmit our manuscript entitled "A high-quality chromosome-level genome assembly of the oligophagous fruit fly Bactrocera tsuneonis (Diptera: Tephritidae) and insights into its host specificity". We would like to express our sincere gratitude to you and the reviewers for your valuable time and insightful comments, which have significantly contributed to the improvement of our manuscript. We have carefully addressed all the comments and revised the manuscript accordingly.<br><br>We hereby confirm that the manuscript has not been published, accepted for publication, or is under consideration for publication elsewhere, either in whole or in part. This work is original, and all necessary acknowledgements have been made. The submission has been approved by all authors and affiliated institutions, and all authors have read and agreed to the final version of the manuscript. Please feel free to contact us if any further information is needed.<br>Yours sincerely,<br>Corresponding Author: Yujia Qin qinyujia@cau.edu.cn; Zhihong Li lizh@cau.edu.cn<br>First Author: Tengda Guo guotd7911@163.com<br><br><br>Reviewer #3<br>Comment 1: This assembly is really PacBio + HiC, the Illumina data was not used for assembly. Illumina data was used in kmer based analysis, but the HiFi data alone could also have been used for this same analysis.<br>Authors' reply: We have revised the text to clarify data usage. Illumina short reads were used only for genome survey and quality evaluation, while the final chromosome-level assembly was generated solely from PacBio HiFi and Hi-C data. Line 102-104: "In this study, we assembled a high-quality chromosome-level genome of B. tsuneonis using a combination of PacBio high-fidelity (HiFi) sequencing, and high-resolution chromosome conformation capture (Hi-C) technologies". Line 170-171: "The Illumina short reads were used solely for this purpose and were not included in the assembly process". Line 379-380: "A total of 24.91 Gb of Illumina short reads, 37.82 Gb of PacBio HiFi long reads, and 58.88 Gb of Hi-C data were generated".<br><br>Comment 2: in the methods, full length transcriptome sequencing was mentioned (essentially IsoSeq), but it was unclear how it was analyzed and how it was used for overall genome annotation, manual annotation of genes (particularly can be useful for chemosensory genes) or tissue specific analyses. How was this data used?<br>Authors' reply: We have revised the Methods section to clarify the use of Iso-Seq data in genome annotation. Line 200-203: "Simultaneously, full-length transcriptomic (Iso-Seq) data were incorporated into the annotation workflow to support transcriptome-based gene prediction. The polished Iso-Seq isoforms were aligned to the assembled genome and used to enhance gene model accuracy". Line 433-435: "Protein-coding genes in the B. tsuneonis genome were predicted using a combination of three approaches: ab initio prediction, homology-based prediction, and full-length transcriptome (Iso-Seq)-based prediction".<br><br>Comment 3: I was able to follow, but really a better description of how plant chemistry

Powered by Editorial Manager® and ProduXion Manager® from Aries Systems Corporation

	is being used in combination of protein structure prediction, modeling, and protein expression needs to be made. Really walk people through how the structure of the insect proteins is being compared to the compounds present in host fruit, etc etc.<br>Authors' reply: We have expanded the manuscript to better describe the integration of plant chemistry with protein modeling and molecular simulations. Line 92-101: "Molecular dynamics (MD) simulation techniques are widely used to predict the binding affinity, binding modes, and interaction mechanisms between proteins and their ligands [28]. In olfactory research, molecular docking is commonly applied to identify binding sites and interaction modes between ORs and their specific volatile ligands, whereas MD simulations further reveal the structural stability and conformational dynamics of receptor-ligand complexes [29]. This method provides advantages in analyzing the three-dimensional structures, charge distributions, and interaction mechanisms of proteins (including ORs), offering new insights into the molecular basis of odorant recognition [27,29]. With the rapid advancement of computational technology, MD simulations have become increasingly efficient, serving as a powerful tool for elucidating protein functions and exploring OR-ligand recognition mechanisms [30]". Line 276-277: "To identify the host-derived volatile compounds of host fruit (Maoping Tangerine) for subsequent GC-MS analysis, volatile chemicals were collected using solid-phase microextraction (SPME)". Line 358-360: "To further evaluate the binding stability and conformational dynamics of odorant receptors (BtsuOr7a-6 and BtsuOr7a-4) complexed with their respective ligands (trans-nerolidol and piperitone), MD simulations were performed". Line 563-566: "Based on the SPME-GC–MS analysis and fluorescence binding assays, trans-nerolidol and piperitone were identified as the two major host-derived volatiles that showed strong binding affinity to BtsuOBP83a. To further elucidate the molecular recognition mechanisms underlying these interactions, molecular docking was performed using the predicted OBP structure".<br><br>Comment 4: Take a closer look at chromosome structure in Tephritid fruit flies. You report changes in chromosome number, but a lot of that is related to chromosome naming. Please attempt to say "autosomes", X and Y or if unknown be clear that the autosomes can't be separated from sex chromosome. One application of Illumina data can be to characterize the chromosome as a sex chromosome based on baised coverage in females vs males (e.g. AD ratio). This could be used to better assign your chromosomes as autosomes (there are 5) and X, rather than reporting as having 6 chromosomes (which is misleading). You also can review classical cytogenetic experiments as evidence.<br>Authors' reply: We have revised the Results section (Table 1) to clarify chromosome naming and to distinguish between autosomes and sex chromosomes. The revised manuscript notes that B. tsuneonis likely possesses five autosomes and one X chromosome, with Chr2 showing collinearity to the D. melanogaster X chromosome. Line 412-413: "Together, these results indicate that B. tsuneonis likely possesses a karyotype of five autosomes and one X chromosome (5 + X)". While Illumina read coverage–based sex chromosome assignment (AD ratio) was not performed in this study, we acknowledge that such analyses, along with classical cytogenetic evidence, will be valuable for future work.<br><br>In addition, we have carefully reviewed all comments and annotations provided in the marked-up PDF from Reviewer #3. Detailed responses have been inserted directly after each comment within the annotated PDF, ensuring that every point has been addressed clearly and specifically. We sincerely appreciate these constructive suggestions, which have further improved the accuracy and clarity of the revised manuscript.
**Additional Information:**	
**Question**	**Response**
Are you submitting this manuscript to a special series or article collection?	No
**Experimental design and statistics**<br><br>Full details of the experimental design and	Yes

statistical methods used should be given in the Methods section, as detailed in our Minimum Standards Reporting Checklist. Information essential to interpreting the data presented should be made available in the figure legends.<br><br>Have you included all the information requested in your manuscript?	
**Resources**<br><br>A description of all resources used, including antibodies, cell lines, animals and software tools, with enough information to allow them to be uniquely identified, should be included in the Methods section. Authors are strongly encouraged to cite Research Resource Identifiers (RRIDs) for antibodies, model organisms and tools, where possible.<br><br>Have you included the information requested as detailed in our Minimum Standards Reporting Checklist?	Yes
**Availability of data and materials**<br><br>All datasets and code on which the conclusions of the paper rely must be either included in your submission or deposited in publicly available repositories (where available and ethically appropriate), referencing such data using a unique identifier in the references and in the "Availability of Data and Materials" section of your manuscript.<br><br>Have you have met the above requirement as detailed in our Minimum Standards Reporting Checklist?	Yes
GigaScience has policies and guidelines in place for the use of generative AI-writing tools such as ChatGPT. If you have used such writing tools to assist with writing the manuscript this must be	No

Powered by Editorial Manager® and ProduXion Manager® from Aries Systems Corporation

declared and cited in the text. Authors should not list AI-writing tools and other AI-assisted technologies as an author or co-author and should acknowledge that they are fully responsible for text generated or refined by AI-writing tools.<p><br><br>A summary of use (particularly in the introduction or among methods) needs to be included at the end of the paper, and the outputs should also be included as a supplementary file hosted in GigaDB or other open repositories. Please read our guidelines for more information. <p><br><br>By submitting to GigaScience, you are aware of the journal's AI-writing tools policy, and if you have declared use of such tools below, you have acknowledged this where appropriate in your manuscript and have made a summary of use and outputs available. <p><br><b>AI-assisted writing tools have been used in the preparation of this manuscript?	

Powered by Editorial Manager® and ProduXion Manager® from Aries Systems Corporation

Manuscript

Click here to access/download;Manuscript;Manuscript.docx

A high-quality chromosome-level genome assembly of the oligophagous fruit fly *Bactrocera tsuneonis* (Diptera: Tephritidae) and insights into its host specificity

Tengda Guo[1,2], Weisong Li[1], Yuan Zhang[1,3], Wenzhao Yang[1], Zhihong Li[1,*], Yujia Qin[1,*]

[1]State Key Laboratory of Agricultural and Forestry Biosecurity, MARA Key Laboratory of Surveillance and Management for Plant Quarantine Pests, College of Plant Protection, China Agricultural University, Beijing 100193, China

[2]Shenzhen Branch, Guangdong Laboratory of Lingnan Modern Agriculture, Key Laboratory of Synthetic Biology, Ministry of Agriculture and Rural Affairs, Agricultural Genomics Institute at Shenzhen Chinese Academy of Agricultural Sciences, Shenzhen 518120, China

[3]School of Ecology and State Key Laboratory of Biocontrol, Shenzhen Campus of Sun Yat-Sen University, Shenzhen 518107, China.

[*]Correspondence: Yujia Qin, E-mail: qinyujia@cau.edu.cn; Zhihong Li, E-mail: lizh@cau.edu.cn

ORCID

Tengda Guo [0009-0002-6040-0906];

Weisong Li [0009-0000-3531-6783];

Yuan Zhang [0000-0001-6027-9064];

Wenzhao Yang;

Zhihong Li [0000-0002-1281-2108];

Yujia Qin [0000-0002-0903-9089]

Abstract

Background: *Bactrocera tsuneonis* is a major pest of citrus, causing significant economic losses in fruit production. It exhibits a highly specialized host preference, primarily infesting citrus fruits. However, the genetic basis underlying its olfactory adaptation and host specificity remains largely unexplored. To elucidate the molecular mechanisms governing host selection in *B. tsuneonis*, we assembled a high-quality chromosome-level genome and performed comparative genomic, transcriptomic, and functional analyses of its chemosensory system.

Results: The genome of *B. tsuneonis* was assembled to a total size of 339 Mb, with a contig N50 of 11.21 Mb and a scaffold N50 of 59.93 Mb. Comparative genomic analysis revealed significant

contractions in chemosensory-related gene families, particularly in odorant-binding proteins (OBPs) and odorant receptors (ORs), maybe suggesting an adaptation to a narrow host range. Transcriptome analysis demonstrated that *BtsuOBP83a* and *BtsuOBP83b* were highly expressed in the antennae, and most ORs were predominantly expressed in the antennae. Functional assays confirmed that *BtsuOBP83a* selectively binds to two citrus volatiles, *trans*-nerolidol and piperitone, with strong affinity. Molecular docking and molecular dynamics simulations further revealed that *BtsuOr7a-6* and *BtsuOr7a-4* specifically interact with these volatiles, suggesting their role in host odor recognition.

Conclusions: Our high-quality genome of *B. tsuneonis* provides a valuable resource for genomic research and offers valuable insights into the genetic basis of its olfactory adaptation and host specificity. The findings highlight key molecular mechanisms underlying host selection and provide potential targets for behavior-based pest management strategies.

Keywords: *Bactrocera tsuneonis*, genome, comparative genomics, olfactory proteins, oligophagous

Introduction

Bactrocera tsuneonis (Miyake) (NCBI:txid104691), Japanese orange fly, belongs to Diptera, Tephritidae, and is one of the most serious pests affecting citrus crops [1]. Its distribution is mainly restricted to China and Japan, but it has the potential to spread beyond Asia [2]. *B. tsuneonis* uses its ovipositor to penetrate unripe citrus fruits for oviposition, and the larvae feed on the internal tissues of the host fruit, causing significant damage to fruit quality and yield [3]. On average, 10% to 20% of citrus yields may be lost due to infestation, and if not effectively controlled, losses could exceed 50% [4]. As citrus is one of the most widely cultivated and produced fruits globally [5], due to the damaging characteristics of *B. tsuneonis* and its recognition as a significant international quarantine pest, this species warrants global attention for strengthened prevention and control efforts. Despite its economic and environmental impact, comprehensive research on the genetic factors contributing to its adaptability and invasiveness remains lacking.

The advancement of genomic tools, particularly the availability of high-quality assembled genomes, has significantly facilitated the investigation of the genetic factors driving the global distribution and diversity of various organisms [6,7]. *Bactrocera* species are highly invasive and adaptable, with females ovipositing in host plants and larvae feeding on the fruit, leading to substantial agricultural losses [8,9]. Genome annotations have been published for eight species within the *Bactrocera* genus (*B. correcta*, *B. dorsalis*, *B. tryoni*, *B. latifrons*, *B. oleae*, *B. neohumeralis*, *B. minax*, and *B. zonata*)

in NCBI [10]. Additionally, *B. correcta*, *B. dorsalis*, *B. tryoni*, *B. oleae*, *B. neohumeralis*, and *B. zonata* have genome assemblies reported at the chromosome level [11]. Given the economic and ecological importance of *B. tsuneonis*, investigating its genetic foundation is essential for advancing research on its biology, ecology, and evolutionary adaptations, as well as for developing more effective pest management strategies.

The host range of *B. tsuneonis* is limited to Citrus species, exhibiting oligophagous, which differs from many polyphagous fruit flies, such as *B. dorsalis*, which can infest fruits from multiple plant families [12]. Understanding how *B. tsuneonis* selects its host to complete oviposition is essential for elucidating its ecological adaptability. In insects, the chemosensory system plays a pivotal role in host localization and recognition, with odorant-binding proteins (OBPs) and odorant receptors (ORs) being particularly crucial for detecting host volatiles [13,14]. OBPs are small, globular, water-soluble acidic proteins that are widely distributed in the lymphatic fluid. Their interaction with odorant molecules constitutes the initial biochemical step in external odor recognition [15]. These proteins typically consist of 120–160 amino acids, with a molecular weight of approximately 15–20 kDa. At the N-terminus, they contain a signal peptide of about 20 amino acids, which is cleaved during secretion to yield the mature protein. A distinctive characteristic of OBPs is the presence of six conserved cysteine residues that form three disulfide bonds (C1–C3, C2–C5, C4–C6), ensuring structural stability [16,17,18]. Functional characterization of OBPs typically involves expressing recombinant proteins in a prokaryotic system, followed by purification and ligand-binding analysis. Fluorescence-based competitive binding assays are commonly employed to identify specific odorant ligands for OBPs, providing insights into their role in olfactory perception [19,20,21].

When odorant molecules reach the membrane of olfactory neurons, they are released and activate ORs, converting chemical signals into electrical impulses that are subsequently processed and transmitted to the central nervous system [22]. ORs, located on the dendritic membranes of olfactory neurons, are key components of the peripheral olfactory system in insects [23]. The specific odorant detected by an OR is referred to as its ligand. Insect ORs are broadly categorized into two types: the atypical odorant receptor co-receptor (ORco) and conventional odorant receptors. ORco is a highly conserved protein across insect species and does not independently recognize odorants [24]. However, conventional ORs require ORco to function properly. Structurally, insect ORs are membrane proteins characterized by seven α-helical transmembrane domains. While they share some structural similarities with mammalian G protein-coupled receptors (GPCRs), insect ORs exhibit a distinct orientation, with the C-terminus located extracellularly and the N-terminus intracellularly [25,26]. Recent cryo-electron microscopy studies have revealed that insect ORs assemble into tetrameric complexes with ORco to facilitate signal transduction. This structural organization is essential for

odor recognition and plays a critical role in insect olfactory perception [27,28,29].

Molecular dynamics (MD) simulation techniques are widely used to predict the binding affinity, binding modes, and interaction mechanisms between proteins and their ligands [30]. In olfactory research, molecular docking is commonly applied to identify binding sites and interaction modes between ORs and their specific volatile ligands, whereas MD simulations further reveal the structural stability and conformational dynamics of receptor-ligand complexes [31]. This method provides advantages in analyzing the three-dimensional structures, charge distributions, and interaction mechanisms of proteins (including ORs), offering new insights into the molecular basis of odorant recognition [29,31]. With the rapid advancement of computational technology, MD simulations have become increasingly efficient, serving as a powerful tool for elucidating protein functions and exploring OR-ligand recognition mechanisms [32].

In this study, we assembled a high-quality chromosome-level genome of *B. tsuneonis* using a combination of PacBio high-fidelity (HiFi) sequencing, and high-resolution chromosome conformation capture (Hi-C) technologies. Through comparative genomic and gene family analyses, we explored the evolutionary dynamics of chemosensory gene families, particularly odorant-binding proteins (OBPs) and odorant receptors (ORs), in *B. tsuneonis* relative to polyphagous fruit flies. Furthermore, we examined the interactions between olfactory-related proteins and key host volatiles using gas chromatography-mass spectrometry (GC-MS), fluorescence competitive binding assay, structural modeling, and MD simulations to elucidate the molecular mechanisms underlying volatile recognition in *B. tsuneonis*. The findings from this study provide a foundational genetic resource for future research, contributing to a deeper understanding of host recognition mechanisms in oligophagous pests and offering theoretical support for the development of behavior-based pest management strategies targeting chemosensory pathways.

Methods

Sample preparation

The *B. tsuneonis* samples used in this study were collected from a natural wild population in Pingshan, Yibin, Sichuan Province. Larval samples were collected in September 2022 from infested fruits in orchards, while adult samples were obtained in January 2020 by excavating pupae of *B. tsuneonis* from infested orchards and bringing them back to the laboratory, where they were kept in a constant temperature and humidity artificial intelligence climate box until emergence. The parameters of the climate box are set at a constant temperature of 25◦C, a humidity level of 70%, and a light period of 10 h of daylight followed by 14 h of darkness. All samples underwent molecular identification prior to experiments, including DNA extraction, and RNA extraction, to confirm that they were *B.*

tsuneonis [33].

Genomic DNA and RNA sequencing

For Illumina sequencing, genomic DNA was extracted from a single male adult (with the abdomen removed) using the Wizard SV Genomic DNA Purification System Kit (Promega, USA). DNA quality and concentration were measured using a microvolume UV spectrophotometer. The genomic DNA was enzymatically fragmented to an average size of 180–250 bp, end-polished, A-tailed, and ligated to full-length adapters for sequencing. The libraries were hybridized with biotin-labeled probes using the SureSelectXT Human All Exon V6 kit (Agilent Technologies, USA), and captured fragments were enriched by PCR amplification and purified using the AMPure XP system (Beckman Coulter, USA). Library quality was verified by agarose gel electrophoresis and qPCR quantification. Paired-end sequencing (2 × 150 bp) was performed on the Illumina NovaSeq platform by Berry Genomics (Beijing, China).

For long-read sequencing, genomic DNA was extracted from a single male adult (with the abdomen removed). A HiFi SMRTbell library (15 kb insert size) was prepared using the SMRTbell Express Template Prep Kit v3.0 (Pacific Biosciences, USA) following the manufacturer's protocol. Briefly, genomic DNA was sheared to approximately 15 kb using the Megaruptor 3 system (Diagenode, USA), followed by DNA damage repair, end-repair, A-tailing, and ligation of SMRTbell adapters. The library was subsequently purified, subjected to nuclease treatment, and size-selected using the Pippin HT system (Sage Science, USA). After quality assessment with the Agilent Fragment Analyzer System (Agilent Technologies, USA), the final library was sequenced by Berry Genomics (Beijing, China) on the PacBio Sequel II platform, generating high-fidelity (HiFi) circular consensus sequence (CCS) reads.

For Hi-C sequencing, genomic DNA was extracted from a single larva after three days of starvation treatment. The samples were fixed with formaldehyde, and DNA was digested using the restriction enzyme DpnII, digesting it into approximately 400 bp fragments. DNA fragments containing interaction relationships were captured to construct the library. Sequencing was performed by Berry Genomics (Beijing, China) on the Illumina Novaseq/MGI-2000 platform.

For full-length transcriptome sequencing, total RNA was extracted from a single abdomen-removed adult using the SV Total RNA Isolation System Kit (Promega, USA). RNA quality was assessed using the Agilent 2100 RNA 6000 Nano Kit (Agilent Technologies, USA), and samples with an RIN > 7.5 were used for library construction. Complementary DNA (cDNA) synthesis and library preparation were performed using the Iso-Seq Express 2.0 Kit and Kinnex Full-Length RNA Kit (Pacific Biosciences, USA) according to the manufacturer's protocols. Briefly, poly(A)-selected RNA was reverse-transcribed into first-strand cDNA using Oligo(dT) primers, followed by template switching

and PCR amplification to obtain full-length cDNA. The amplified products were enzymatically treated, purified, and converted into a 1–10 kb SMRTbell library. After quality validation with the Agilent Fragment Analyzer System (Agilent Technologies, USA), the final library was sequenced by Berry Genomics (Beijing, China) on the PacBio Sequel II platform.

For transcriptome sequencing, different body parts, including 50 antennae, 5 heads (without antennae), 10 legs, and 5 ovipositors (female only), were dissected separately from field-collected female and male adults. Three biological replicates were prepared for each tissue type. Total RNA was extracted using standard protocols. RNA sequencing libraries were constructed using the Illumina TruSeq RNA Library Preparation Kit and sequenced on an Illumina platform by Personalbio (Shanghai, China), generating 150 bp paired-end reads.

Genome assembly and evaluation

To assess the genome characteristics of *B. tsuneonis*, including genome size, heterozygosity, repeat content, and GC composition, a genome survey was conducted using Illumina sequencing data. The Illumina short reads were used solely for this purpose and were not included in the assembly process. K-mer analysis was performed with Jellyfish version 2.2.1 [34] to generate the k-mer frequency distribution, followed by genome statistical evaluation using GenomeScope version 2.0 [35].

For genome assembly, Hifiasm v0.19.3 was used to assemble the PacBio HiFi reads into a high-quality draft genome [36]. For highly heterozygous genomes, the initial assembly may contain redundant haplotigs, resulting in an inflated genome size. To address this, the assembly was processed with Purge_dups v1.2.3 to remove redundant sequences and obtain a haploid representation of the genome [37].

To anchor the genome assembly to chromosome-scale linkage groups, Hi-C analysis was conducted. Following quality filtering of the Hi-C reads, the cleaned Hi-C reads were mapped to the draft genome using BWA version 0.7.17 [38]. Paired-end reads uniquely aligned to the draft genome were selected based on restriction sites identified from the Hi-C data. Using 3D-DNA version 180114 and Juicer version 1.6, reads were then clustered to build scaffolds [39,40]. Scaffold arrangement was validated by assessing interaction strengths between read pairs. The scaffold order and orientation were manually inspected and adjusted using Juicebox v1.11.08, ensuring accurate chromosomal placement [41].

Genome annotation

To identify and annotate repetitive elements in the *B. tsuneonis* genome, we employed RepeatMasker version 4.1.5 using Dfam release 3.8 and RepBase edition 20181026 as reference databases [42,43,44]. A de novo repeat library was constructed with RepeatModeler version 2.0.5 [45]. Long

terminal repeat (LTR) retrotransposons were identified using LTR Finder version 1.0726 and LTR Retriever version 2.9.028 [46,47]. Tandem repeats were annotated with Tandem Repeats Finder version 4.09.1 [48].

A comprehensive gene annotation pipeline integrating ab initio, homology-based, and transcriptome-based predictions was applied to establish a high-confidence gene set. First, Augustus version 3.3.3 [49] and GlimmerHMM version 3.0.4 [50] were used for ab initio prediction, leveraging species-specific training models and hidden Markov models (HMMs) to identify potential coding regions. Second, homology-based prediction was conducted using GeMoMa version 1.9 [51], incorporating protein sequences from *D. melanogaster*, *B. minax*, *B. correcta*, *B. dorsalis*, *B. oleae*, *B. latifrons*, and *B. tryoni* to improve gene model reliability. Simultaneously, full-length transcriptomic (Iso-Seq) data were incorporated into the annotation workflow to support transcriptome-based gene prediction. The polished Iso-Seq isoforms were aligned to the assembled genome and used to enhance gene model accuracy. Open reading frames (ORFs) and complete protein-coding sequences (CDSs) were identified using TransDecoder version 5.1.0 [52]. Subsequently, EVidenceModeler version 1.1.1 [53] was used to integrate the results from the three approaches, assigning different weights based on the confidence level of each data source to generate a comprehensive gene set. Finally, PASA version 2.5.2 [53] was applied to refine gene models by correcting exon boundaries, annotating untranslated regions (UTRs), and identifying novel transcripts, resulting in a high-quality genome annotation dataset.

Functional annotation of protein sequences was performed using multiple databases and tools: (i) Diamond version 2.1.8.162 [54] for the NCBI nr database; (ii) InterProScan version 5.63-95.0 [55] for annotating Gene Ontology (GO) terms, signal peptides (SignalP), and InterPro annotations; and (iii) eggNOG-mapper version 2.1.12 [56] to annotate Clusters of Orthologous Genes (COG) categories and KEGG pathways.

Chromosomal synteny analysis

To investigate the structural characteristics of the *B. tsuneonis* genome, a chromosomal synteny analysis was conducted using two reference species: the model organism *D. melanogaster* (NCBI: GCF_000001215.4) and the closely related *B. dorsalis* (NCBI: GCA_023373825.1). These chromosome-level genomes were selected from published Tephritidae genomes for comparative analysis. Synteny analysis was performed using the One Step MCScanX tool in TBtools-II [57]. Genome sequence files and corresponding GFF annotation files were provided as input to detect and analyze homologous gene blocks. The Dual Synteny Plot for MCScanX tool was subsequently used to generate visual representations of syntenic relationships between chromosomes based on the processed configuration files.

Orthology prediction and inference of phylogenetic relationships

To infer the phylogenetic relationships of *B. tsuneonis* with other insect species, we selected 16 additional species for comparative analysis (Fig. 2). The complete protein sequences of 17 insect species were used, with *D. melanogaster* designated as the outgroup. OrthoFinder version 2.5.4 [58] was employed to identify gene families across the selected species. Based on the OrthoFinder results, gene family clusters were categorized into four groups: single-copy genes, multiple-copy genes, species-specific (unique) genes, and unassigned genes. Functional annotation of gene families was performed using KinFin version 1.0 [59], which assigned dominant functional categories based on the most prevalent annotations among cluster members.

The phylogenetic tree was constructed based on the multiple sequence alignment of single-copy orthologous genes from each species. Multiple sequence alignments were filtered using TrimAl version 1.4.rev15 [60], and a maximum-likelihood phylogenetic tree was inferred using raxmlHPC-PTHREADS [61] based on the processed sequences.

Divergence times were estimated using the MCMCTree tool in PAML version 4.10.7 [62] based on the approximate likelihood method. Calibration points were determined from previous studies and three reference points from the TIMETREE database [63]: *Z. cucurbitae–Z. tau* (9.8 Mya) [64], *Zeugodacus–Bactrocera* (21.6–86.3 Mya) [64,65,66], Tephritidae–Drosophilidae (111.4–149 Mya) [64,67,68,69]. Visualization and analysis of phylogenetic trees were performed using tvBOT [70].

Gene family analysis

Gene family expansion and contraction among species were analyzed using CAFE version 5 [71], with OrthoFinder results and the phylogenetic tree, including divergence time estimates, as input data. The analysis accounted for phylogenetic tree topology and branch lengths when evaluating the significance of gene family size changes in each branch. Gene families with conditional P-values below 0.05 were considered to have undergone a significantly accelerated rate of expansion or contraction.

Manual annotation was performed for five detoxification-related gene families, including ATP-binding cassette (ABC) transporters, glutathione S-transferases (GSTs), cytochrome P450 monooxygenases (CYP450s), UDP-glucuronosyltransferases (UGTs), and carboxyl/cholinesterases (CCEs). Additionally, the heat shock protein (HSP) family and chemosensory-related gene families, including odorant-binding proteins (OBPs), odorant receptors (ORs), ionotropic receptors (IRs), gustatory receptors (GRs), chemosensory proteins (CSPs), and sensory neuron membrane proteins (SNMPs), were also manually annotated. HMMs for these gene families were retrieved from the Pfam database [72]. Reference protein sequences for each gene family in *D. melanogaster* were obtained from FlyBase [73] and the NCBI database.

To identify gene family members, both BLAST version 2.10.0 [74] and HMMER version 3.3.2 [75] were employed, with BITACORA version 1.3 [76] used to integrate results in protein mode, applying an e-value threshold of 1e-5. Protein sequences of annotated OR and OBP genes were aligned using MUSCLE version 3.8.1551 [77]. Phylogenetic trees were constructed with IQTREE version 2.2.3 [78] using the neighbor-joining method, with 1000 bootstrap replicates. Tree visualization and annotation were performed using tvBOT.

Transcriptomes of different tissues

Clean reads from transcriptome sequencing of different tissues were aligned to the assembled *B. tsuneonis* genome using Hisat2 version 2.2.1 [79]. Quantification analysis was conducted using Rsubread version 2.18.0 [80]. The expression levels of OBPs and ORs in various tissues were visualized as heatmaps generated using OmicShare tools [81]. To validate the reliability of transcriptome-based expression levels, qRT-PCR was performed using antennal RNA for five OBP genes (*BtsuOBP83a*, *BtsuOBP83b*, *BtsuOBP19a-1*, *BtsuOBP84a-1*, and *BtsuOBP28a-2*) and five OR (*BtsuORco*, *BtsuOR7a-4*, *BtsuOR67c-1*, *BtsuOR30a-3*, and *BtsuOR7a-6*) with the highest antenna-specific expression. Relative expression values were compared with RNA-seq data to assess correlation between the two methods. Primer sequences are provided in Supplementary Table S1.

Chemical extracts

To identify the host-derived volatile compounds of host fruit (*Maoping Tangerine*) for subsequent GC-MS analysis, volatile chemicals were collected using solid-phase microextraction (SPME). Approximately 5 g of fruit was placed in a 20 mL sample vial, which was then sealed with a silicone septum-containing cap. Headspace extraction was conducted using a manual sampler equipped with a preconditioned 50/30 μm DVB/CAR/PDMS fiber. The fiber was inserted into the vial and exposed to the headspace for 40 minutes at an extraction temperature of 30°C. Following extraction, the fiber was immediately transferred to the gas chromatography (GC) injection port for desorption for 5 minutes. To minimize background interference, a blank sample (empty vial) was processed using the same protocol at the beginning of each experimental group. The 50/30 μm DVB/CAR/PDMS fiber was conditioned at 270°C for 30 minutes before its first use [82]. Sample vials were cleaned sequentially with distilled water and anhydrous ethanol, air-dried, and baked at 200°C for 2 hours to eliminate potential contamination. The extraction was performed in three biological replicates.

Gas chromatography coupled with mass spectrometry (GC-MS) analysis

Volatile compounds from host fruits were analyzed using a 7890B/7200 Quadrupole Time-of-Flight GC/MS system (Agilent, USA). The GC inlet temperature was set to 260°C, and analyses were performed in splitless mode. High-purity helium gas (99.999%) was used as the carrier gas at a constant flow rate of 1.0 mL/min, with a column head pressure of 0.102 kg/cm^2 (1.45 psi). The column

temperature program was as follows: initial temperature: 40°C (held for 2 min), ramp rate: 8°C/min to 260°C, final temperature: 260°C (held for 1 min). The interface and ion source temperatures were maintained at 270°C. Mass spectra were acquired in electron ionization (EI) mode with an electron energy of 70 eV and an emission current of 25 μA. The scan range was set to 45–500 m/z at a scan rate of 5 scans per second. Identification of volatile compounds was performed by comparing GC-MS spectra against reference spectra in the NIST11.L mass spectral library using computerized searches. Additionally, compound identities were further verified by referencing published mass spectra. The relative content of each volatile component was quantified using the peak area normalization method.

Expression and purification of BtsuOBPs

Full-length sequences of *BtsuOBP83a* and *BtsuOBP83b* were amplified using specific primers (Supplementary Table S1). Purified PCR products were cloned into the pGEM-T vector (Promega, USA) for sequencing verification. The confirmed target fragments were then subcloned into the pET-30a(+) expression vector (Sangon, China) using restriction enzymes (Supplementary Table S2) and T4 DNA ligase (Takara, China). Recombinant plasmids were transformed into Escherichia coli BL21 (DE3) competent cells (Tiangen, China). Bacterial cultures were grown to an OD600 of 0.6–0.8, after which protein expression was induced by adding isopropyl-β-D-thiogalactopyranoside (IPTG) (Solarbio, China) to a final concentration of 1 mM at different temperatures. Cells were harvested by centrifugation (5000 × g, 15 min), lysed in PBS buffer via sonication, and subjected to SDS-PAGE analysis after heat treatment. Large-scale protein expression was performed under optimized induction conditions. Soluble proteins in the supernatant were purified twice using Ni-affinity chromatography (GE Healthcare, USA). His-tags were removed using enterokinase (Novoprotein, China). All purification steps were conducted at 4°C. The size and purity of OBP proteins were evaluated via SDS-PAGE, and protein concentrations were determined using the Bradford method [83].

Fluorescence competitive binding assays

The ligand-binding affinities of candidate OBPs were evaluated using fluorescence competitive binding assays performed on an F-380 Fluorescence Spectrophotometer (Tianfang, China), with modifications to the standard procedure. Purified OBP proteins were diluted to a final concentration of 2 μmol/L in 50 mmol/L Tris-HCl buffer (pH 7.4). The fluorescent probe N-phenyl-1-naphthylamine (1-NPN) and all candidate volatile ligands were dissolved in HPLC-grade methanol at an initial concentration of 1 mmol/L. The binding constant between OBPs and 1-NPN was determined by recording emission spectra from 350 to 470 nm at an excitation wavelength of 337 nm. To establish binding saturation, titrations were conducted by sequentially adding 1-NPN to the protein

solution, reaching final concentrations of 2–20 μmol/L in 2 μmol/L increments. Fluorescence intensity was measured after stabilization, and each measurement was performed in triplicate. To assess the binding affinities of OBPs for candidate volatile ligands, competition assays were performed by introducing increasing concentrations of each ligand (final concentration range: 4–32 μmol/L, in 4 μmol/L increments). Each experiment was conducted in triplicate. Dissociation constants (*Ki*) for the volatile ligands were calculated from the corresponding *IC50* values using the following equation:

$$K_i = \frac{[IC_{50}\ ]}{1 + \frac{[1 - NPN]}{K_{1-NPN}}}$$

where [*IC50*] is the concentration of the competitor that reduces the initial fluorescence intensity by half, [1-NPN] is the free concentration of 1-NPN, and is the dissociation constant of the protein/1-NPN complex.

Protein structure prediction and molecular docking

The three-dimensional (3D) structures of OBPs and ORs were predicted using AlphaFold2 [84], installed on a local server. The default AlphaFold2 pipeline was used for structural modeling. For ORs, we predicted the heteromeric structures of all ORs expressed in the antennae of *B. tsuneonis*, assuming a stoichiometry of two ORs and two ORco subunits. Given that these ORs can theoretically assemble in either adjacent or diagonal positions within the homotetrameric complex, we generated ten structural models for each OR-ORco complex to assess the most favorable configuration. Comparative analysis of these models indicated that the diagonal arrangement was more likely than the adjacent configuration.

The 3D structures of target ligands were downloaded from the PubChem database [85]. Binding pockets (active sites) in OBPs and ORs were predicted using DoGSite3 [86,87,88], and Grid Boxes were manually defined in PyMOL version 3.0 [89] to fully encompass the predicted binding cavities. Docking simulations were performed using AutoDock Vina version 1.2.x [90], with docking parameters optimized based on the structural characteristics of the proteins and their predicted active sites. The best docking models were selected based on binding affinity scores (kcal/mol). Protein-ligand interactions were visualized and analyzed using PyMOL. For OR models, considering the symmetrical nature of the homotetrameric OR-ORco complex, binding modes in the two OR pockets were assumed to be identical.

Molecular dynamics simulations and analysis

To further evaluate the binding stability and conformational dynamics of odorant receptors (BtsuOr7a-6 and BtsuOr7a-4) complexed with their respective ligands (trans-nerolidol and

piperitone), MD simulations were performed. All simulations were carried out using GROMACS v2022.3 to investigate the structural stability and molecular interactions within each receptor-ligand system [91,92]. During small molecule preprocessing, AmberTools22 was used to assign the GAFF force field, while Gaussian 16W handled hydrogenation and RESP potential calculations. The resulting potential parameters were integrated into the molecular system's topology file. Simulations were conducted under constant temperature (300 K) and atmospheric pressure (1 bar), employing the Amber99sb-ildn force field with TIP3P water molecules as the solvent. System neutrality was maintained by adding Na^+ ions. The simulation workflow consisted of three phases: first, energy minimization was performed using the steepest descent algorithm; second, equilibration simulations were carried out under isothermal-isovolumetric (NVT) and isothermal-isobaric (NPT) ensembles, each lasting 100 ps with a coupling constant of 0.1 ps and comprising 100,000 steps; finally, a 100 ns production simulation was conducted, consisting of 5,000,000 steps with a 2 fs time step. Post-simulation analysis was performed using built-in GROMACS tools to evaluate key dynamic properties, including root-mean-square deviation (RMSD), root-mean-square fluctuation (RMSF), and the radius of gyration. Additionally, molecular mechanics generalized Born surface area (MMGBSA) and free energy landscape analyses were conducted to further assess binding stability and conformational changes.

Results

Genome sequencing and assembly

A total of 24.91 Gb of Illumina short reads, 37.82 Gb of PacBio HiFi long reads, and 58.88 Gb of Hi-C data were generated (Supplementary Table S3). Based on k-mer analysis with k = 19, the estimated genome size of *B. tsuneonis* was approximately 324 Mb, with a heterozygosity rate of 1.61% and a repeat content of 15.1% (Fig. 1A).

At the contig level, the final draft genome assembly measured 342.91 Mb and consisted of 75 contigs with an N50 length of 11.21 Mb. This genome size aligns closely with the k-mer-based estimate (324 Mb) but is smaller than those of other Bactrocera species, such as *B. dorsalis* (530.3 Mb), *B. correcta* (702.7 Mb), *B. oleae* (468.8 Mb), *B. latifrons* (462.5 Mb), and *B. tryoni* (570.6 Mb), though slightly larger than *B. minax* (325.3 Mb) (Table 1). Notably, the *B. tsuneonis* genome exhibited a significantly higher contig N50 (11.21 Mb) than other *Bactrocera* species, including *B. minax* (27.4 kb), *B. dorsalis* (1.5 Mb), *B. correcta* (221.9 kb), *B. latifrons* (31.5 kb), and *B. tryoni* (350.9 kb) (Table 1). The GC content of *B. tsuneonis* (34.66%) was slightly lower than that of other *Bactrocera* species, which ranged from 34.5% to 36.5% (Table 1). BUSCO analysis confirmed the high completeness of the contig-level genome, with 99.5% of expected genes identified (Supplementary Table S4).

Using Hi-C to assist in genome assembly, we obtained a high-quality chromosome-level genome assembly of *B. tsuneonis* with a total size of 339 Mb (Table 1). The final assembly consisted of 24 scaffolds, with a scaffold N50 of 59.93 Mb. A total of 334.1 Mb (98.55%) of contigs were successfully anchored to six chromosomes (Fig. 1B and 1C). Chromosome lengths ranged from 14.95 Mb to 78.77 Mb (Fig. 1B and 1C). BUSCO analysis further validated the completeness and accuracy of the chromosome-level genome, with 99.1% of genes successfully identified, including 98.0% single-copy genes and 1.1% duplicated genes (Supplementary Table S4). These results collectively confirm the high quality of the *B. tsuneonis* genome, making it suitable for downstream analyses.

Chromosomal synteny analysis was performed to investigate the conservation of gene order and positional relationships between *B. tsuneonis* and related species. A total of 16,964 syntenic genes were identified between *B. tsuneonis* and *B. dorsalis*, representing 62.07% of the total gene count. In contrast, only 6,693 syntenic genes were detected in *D. melanogaster*, accounting for 23.57% of its total genes. These findings suggest extensive chromosomal synteny among the three species, with a notably higher degree of genomic similarity between *B. tsuneonis* and *B. dorsalis*. As shown in Figure 1D, *B. tsuneonis* and *B. dorsalis* exhibit a high level of gene collinearity, with a substantial number of homologous genes maintaining a conserved arrangement. Additionally, Chr02 of *B. tsuneonis* and Chr04 of *B. dorsalis* exhibit collinearity with the X chromosome of *D. melanogaster*, suggesting they may represent putative sex chromosomes in these species. However, no syntenic genes associated with the *D. melanogaster* Y chromosome were identified in the six assembled chromosomes or unanchored scaffolds of *B. tsuneonis*. Together, these results indicate that B. tsuneonis likely possesses a karyotype of five autosomes and one X chromosome (5 + X).

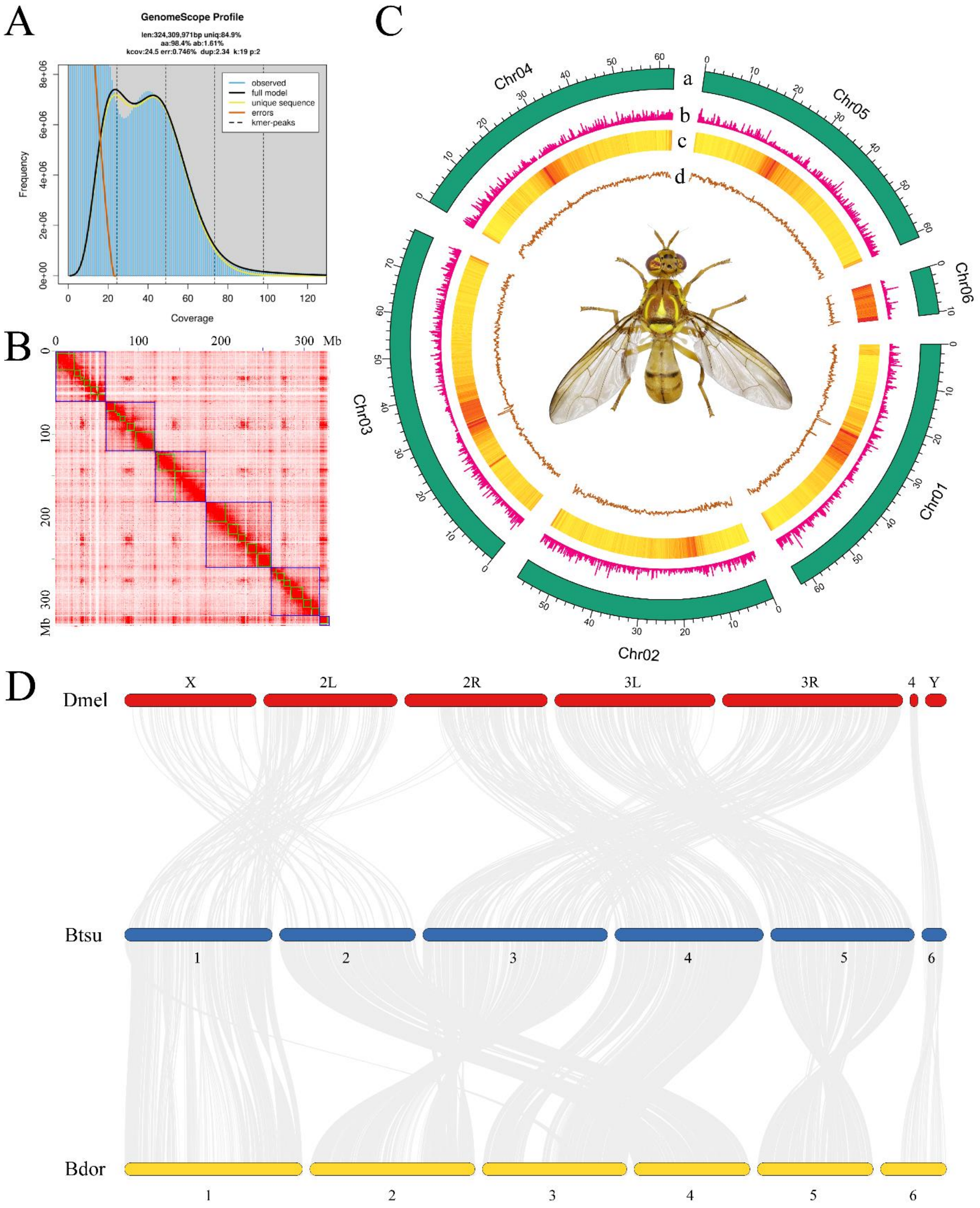

Figure 1: Genome description of *B. tsuneonis*. (A) GenomeScope estimation of genome size and heterogeneity using a k-mer of 19. (B) Hi-C interaction map produced by 3D-DNA. (C) Circular representation of the chromosomes. Tracks a–d represent the distribution of chromosome karyotypes, gene density, repeat sequences density, and GC density, respectively. (D) Synteny blocks among *B. tsuneonis* (Btsu), *D. melanogaster* (Dmel), and *B. dorsalis* (Bdor) genomes.

Table 1: Genome features of 7 *Bactrocera*

Feature	*Bactrocera tsuneonis*	*B. minax*	*B. dorsalis*	*B. correcta*	*B. oleae*	*B. latifrons*	*B. tryoni*
Assembly level	Chromosome	Scaffold	Chromosome	Chromosome	Chromosome	Scaffold	Chromosome
Genome size	339 Mb	325.3 Mb	530.3 Mb	702.7 Mb	468.8 Mb	462.5 Mb	570.6 Mb
Contig N50	11.21 Mb	27.4 kb	1.5 Mb	221.9 kb	-	31.5 kb	350.9 kb
Scaffold N50	59.93 Mb	97.4 kb	93.3 Mb	100.6 Mb	75.1 Mb	974.4 kb	81.9 Mb
Chromosomes	5+X	-	6*	6*	5+XY	-	5
BUSCO	99.2%	98.83%	99.0%	92.6%	99.4%	99.3%	99.2%
GC content	34.66%	35%	36.5%	35.5%	35%	36%	36.5%
Protein-coding genes	13,513	21,924	14,607	17,629	12,391	12,759	14,221
Repetitive elements	24.17%	26.33%	41.57%	58.22%	45.14%	33.16%	42.84%
GenBank	GCA_046562955.1	GCA_029783545.1	GCA_023373825.1	GCA_027475135.1	GCA_042242935.1	GCA_001853355.1	GCA_016617805.2

* The number of autosomes and sex chromosomes was not distinguished

Genome annotation

In the assembled *B. tsuneonis* genome (339 Mb), 24.17% of the sequences were identified as repetitive elements. This proportion is lower than that observed in other *Bactrocera* species, including *B. minax* (26.33%) and *B. latifrons* (33.16%), and substantially lower than *B. correcta* (58.22%), *B. oleae* (45.14%), *B. dorsalis* (41.57%), and *B. tryoni* (42.84%) (Table 1). Among the transposable elements, long interspersed nuclear elements (LINEs) accounted for 1.82%, long terminal repeat (LTR) elements comprised 1.63%, and DNA transposons constituted 4.63% of the genome. Additionally, 203,409 simple repeat elements were identified, representing 2.72% of the *B. tsuneonis* genome (Supplementary Table S5).

Protein-coding genes in the *B. tsuneonis* genome were predicted using a combination of three approaches: ab initio prediction, homology-based prediction, and full-length transcriptome (Iso-Seq)-based prediction. A total of 14,529 protein-coding genes were identified, supported by all three

methods. This number is lower than that of *B. dorsalis* (14,607), *B. correcta* (17,629), and *B. tryoni* (14,221) and is significantly reduced compared to the closely related species *B. minax* (21,924) (Table 1). Functional annotation of the predicted genes revealed that 12,969 (89.26%), 8,926 (60.31%), and 10,483 (72.15%) genes matched entries in the NR, SwissProt, and Pfam databases, respectively. Additionally, 9,073 genes (62.45%) were assigned GO terms, while 7,539 (51.89%) were mapped to KEGG pathways. Overall, 13,513 genes (93.01% of the total protein-coding genes) were successfully annotated across all databases (Supplementary Table S6).

Orthology prediction and inference of phylogenetic relationships

Orthologous gene analysis was conducted on *B. tsuneonis*, its closely related species *B. minax*, the model species *D. melanogaster*, and 14 additional species from the Tephritidae family (Fig. 2; Supplementary Table S7). Gene family clustering was categorized into four groups: single-copy genes, multiple-copy genes, species-specific genes (unique genes), and unassigned genes. OrthoFinder analysis clustered 367,296 genes from 17 species into 33,010 unique gene families (orthogroups). Phylogenetic reconstruction based on single-copy orthologous genes revealed that all *Bactrocera* species formed a distinct clade. For *B. tsuneonis*, 14,529 genes were assigned to 12,543 gene families, including 23 species-specific genes. Divergence time estimation using MCMCTree suggested that *B. tsuneonis* and *B. minax* diverged approximately 4.3 Mya. The split between *Bactrocera* and *Zeugodacus* was estimated at around 59.3 Mya (Fig. 2).

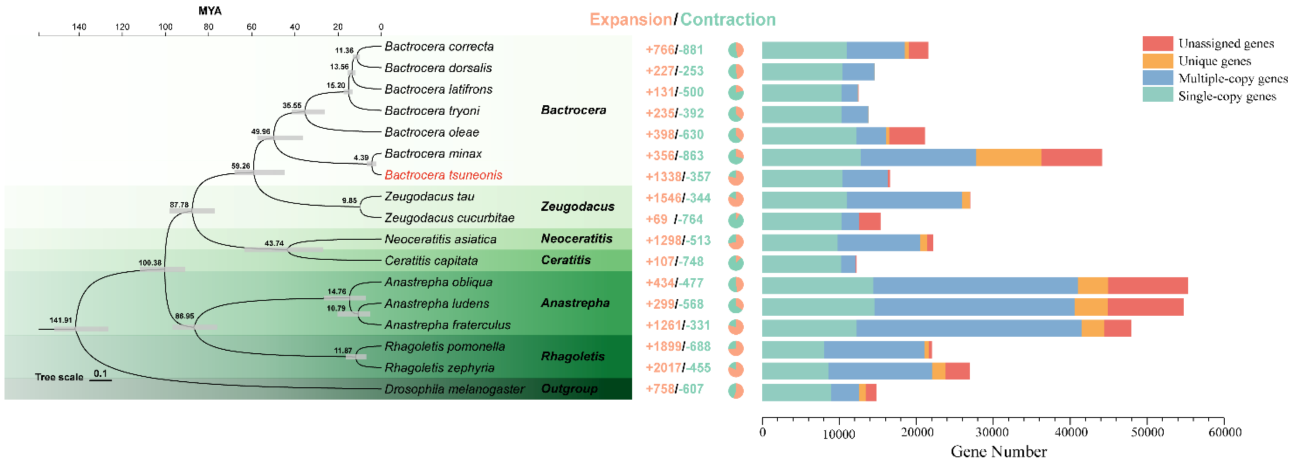

Figure 2: Phylogenetic tree with the dynamic evolution of gene families among *B. tsuneonis*, *B. minax*, and other species.

Gene families associated with adaptability and invasiveness

Using CAFE, we analyzed gene family expansion and contraction during the evolutionary process of *B. tsuneonis*. A total of 559 gene families were found to be expanded, while 1,166 gene families underwent contraction (Fig. 2). Functional annotation with KinFin revealed that chemosensory-related genes, including ORs, OBPs, and GRs, were predominantly found in the contracted gene

families. In contrast, genes associated with detoxification and metabolism, such as CYP450s and ABCs, were enriched in the expanded gene families. These evolutionary changes suggest that adaptations in these gene families have played a critical role in the invasion and ecological adaptation of *B. tsuneonis*. Based on these results, we further examined multiple gene families associated with environmental adaptability, including chemosensory-related genes (OBPs, ORs, IRs, GRs, and CSPs), heat shock proteins (HSPs), and detoxification-related genes (ABCs, GSTs, P450s, UGTs, and CCEs) (Fig. 3). The identification of these gene families provides insights into the genetic mechanisms underlying insect adaptation, which are essential for their survival and ecological success.

To mitigate the effects of harmful substances such as plant secondary metabolites and pesticides, insects have evolved a sophisticated detoxification system. The major detoxification enzymes include CYP450s and GSTs, alongside additional functional gene families such as CCEs, UGTs, and ABCs [93,94,95]. In the *B. tsuneonis* genome, we identified 119 CYP450s, 65 ABCs, 30 UGTs, 40 GSTs, and 7 CCEs (Fig. 3A; Supplementary Table S8). Compared to other *Bactrocera* species, *B. tsuneonis* has a similar number of GSTs and UGTs, while its CCEs and CYP450s counts are lower. However, the number of ABCs is slightly higher. Heat shock proteins play a crucial role in enabling insects to tolerate environmental stressors such as extreme temperatures, oxidative stress, and heavy metal exposure [96,97,98]. In *B. tsuneonis*, we identified five HSP subfamilies, comprising a total of 91 HSP genes: 19 HSP20s, 41 HSP40s, 10 HSP60s, 18 HSP70s, and 3 HSP90s (Fig. 3A; Supplementary Table S8). Compared to other *Bactrocera* species, *B. tsuneonis* exhibits a higher number of HSP genes.

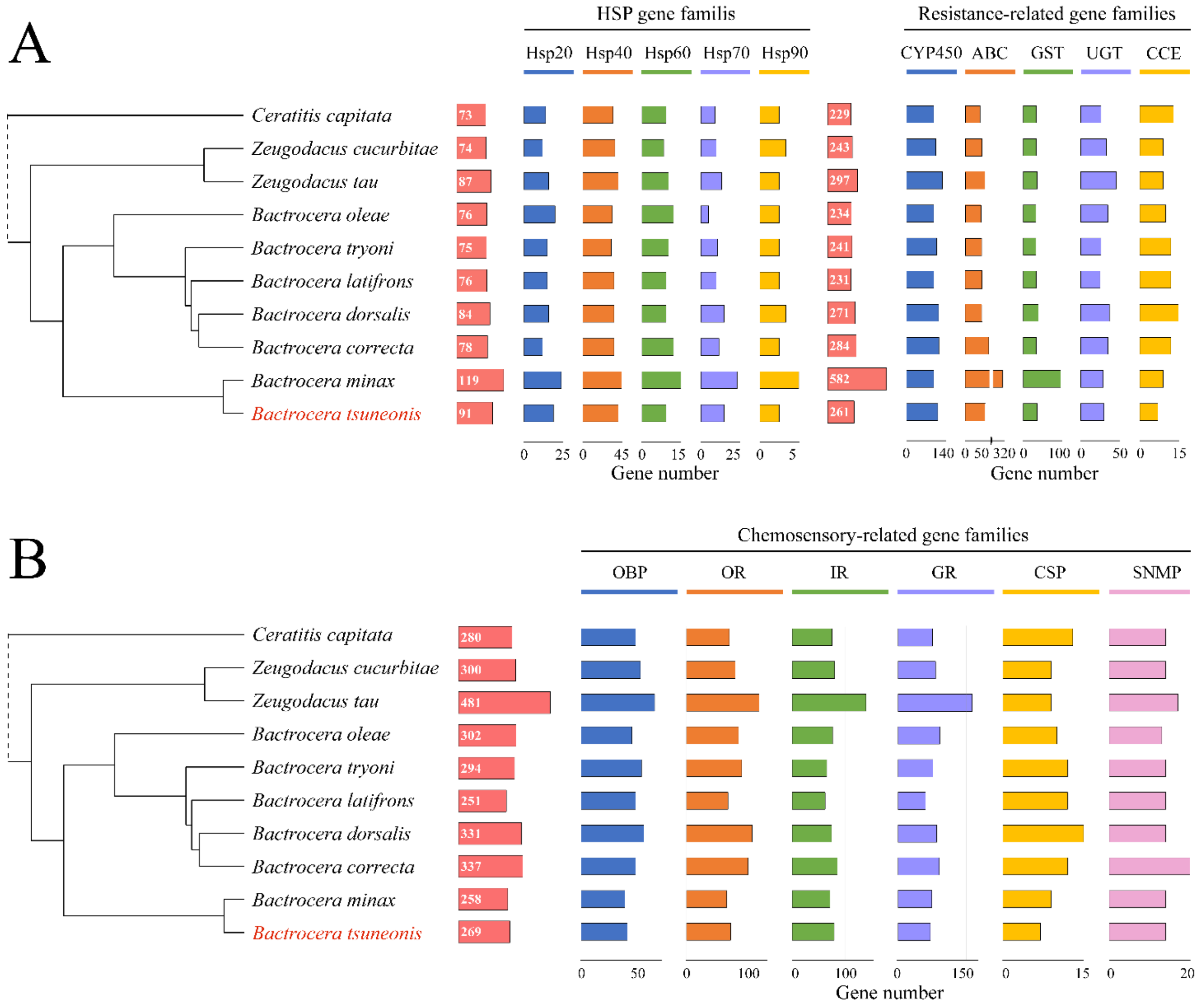

Figure 3: Comparison of gene numbers of (A) heat shock protein gene families, detoxification-related gene families and (B) chemosensory-related gene families in *B. tsuneonis*, and other species.

Genes associated with chemosensory systems

Chemosensory-related gene families in insects include OBPs, GRs, ORs, IRs, CSPs, and SNMPs. These gene families are essential for key behaviors such as feeding, mating, and predator avoidance [99,100,101]. In this study, we identified six chemosensory-related gene families in the *B. tsuneonis* genome, revealing a significant reduction in OBP, OR, GR, and CSP gene counts compared to other *Bactrocera* species, whereas IR and SNMP gene numbers remained relatively stable. Specifically, we identified 39 OBPs, 68 ORs, 79 IRs, 62 GRs, 7 CSPs, and 14 SNMPs in *B. tsuneonis* genome (Fig. 3B; Supplementary Table S8).

Phylogenetic analysis (Fig. 4A) of OBP genes in *B. tsuneonis*, *D. melanogaster*, and *B. correcta*. It is evident that the oligophagous *B. tsuneonis* has significantly fewer OBP genes than the polyphagous *B. correcta*. The OBPs of *B. correcta* are more closely related to those of *D. melanogaster*, such as Dmellush, a protein known to be involved in pheromone-binding activity [102], which was not

identified in *B. tsuneonis*. Additionally, OBP genes of the same species do not cluster into species-specific branches but instead cluster based on different subfamilies. Classical OBPs are distributed across different evolutionary clades. Similarly, phylogenetic analysis of OR genes (Fig. 4B) suggests that the OR gene family in *B. tsuneonis* has undergone large-scale contraction compared to *B. correcta*. Notably, in the OR-VI and OR-VII groups, we observed specific contractions in *B. tsuneonis* compared to *B. correcta*, particularly in OR7a and OR59a. These findings suggest that *B. tsuneonis* has experienced selective gene losses in key chemosensory gene families, potentially influencing its host plant specificity and ecological adaptations.

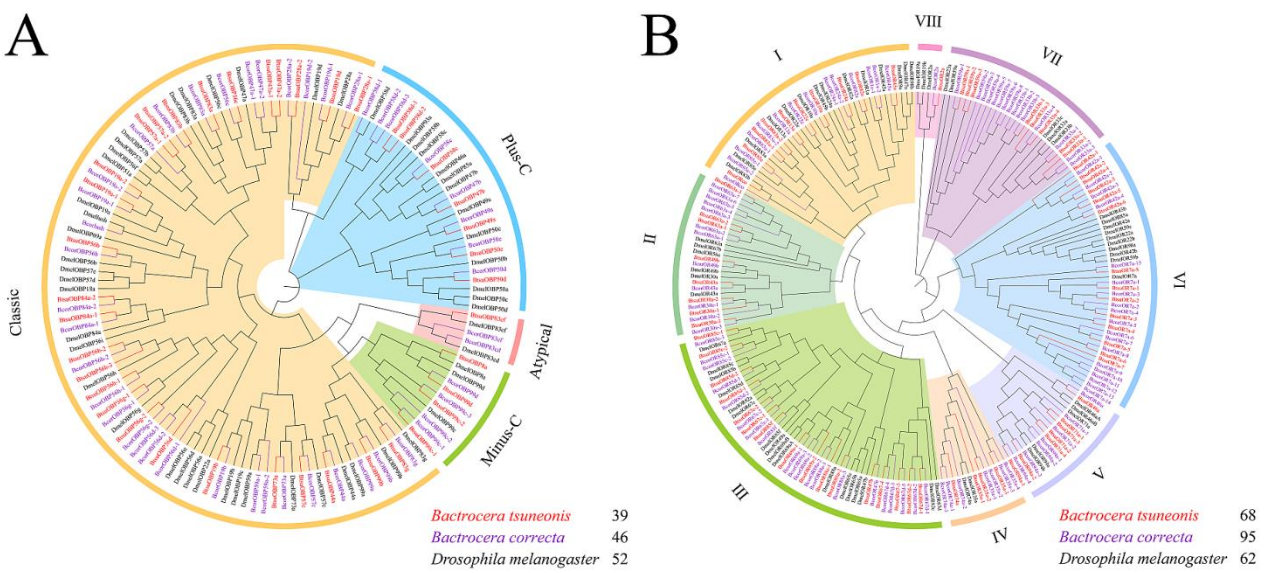

Figure 4: Phylogenetic relationships of *Bactrocera tsuneonis* (Btsu) (A) odorant-binding protein (OBP), (B) odorant receptor (OR) in comparison with *Bactrocera correcta* (Bcor) and *Drosophila melanogaster* (Dmel).

A total of 55.37 Gb of clean reads were obtained through sequencing and used for tissue-specific expression analysis. The expression profiles of *BtsuOBPs* across different body parts of male and female adult flies were visualized using a heatmap (Fig. 5A). The results revealed that *BtsuOBPs* exhibit broad expression patterns, indicating that many OBPs are not restricted to a single body part. Notably, similar expression trends were observed between males and females for the same *BtsuOBPs*. Among the identified OBPs, 13 *BtsuOBPs* displayed relatively high expression levels in the antennae, while five exhibited elevated expressions in the ovipositor. Several *BtsuOBPs*, including *BtsuObp99b*, *BtsuOBP28a-1*, and *BtsuOBP83g*, were also expressed in the legs. Notably, *BtsuOBP83a* and *BtsuOBP83b* demonstrated the highest expression levels in the antennae (Fig. 5C), suggesting their essential roles in olfactory perception.

Similarly, transcriptome analysis of *BtsuORs* revealed distinct expression patterns across various body parts in adult male and female flies. As shown in Figure 5B, most *BtsuORs* were predominantly

expressed in the antennae, while only a few were detected in the legs or ovipositor. These findings highlight the central role of ORs in antennal-mediated olfactory function in *B. tsuneonis*. To further verify the transcriptome-based expression profiles, qRT-PCR was performed using antennal RNA for five OBP genes and five OR genes that showed high antennal expression in RNA-seq data. The qRT-PCR results exhibited expression patterns consistent with the transcriptomic analysis, showing a strong correlation between the two datasets (Supplementary Fig. S1) and supporting the reliability of the RNA-seq–based gene expression profiles.

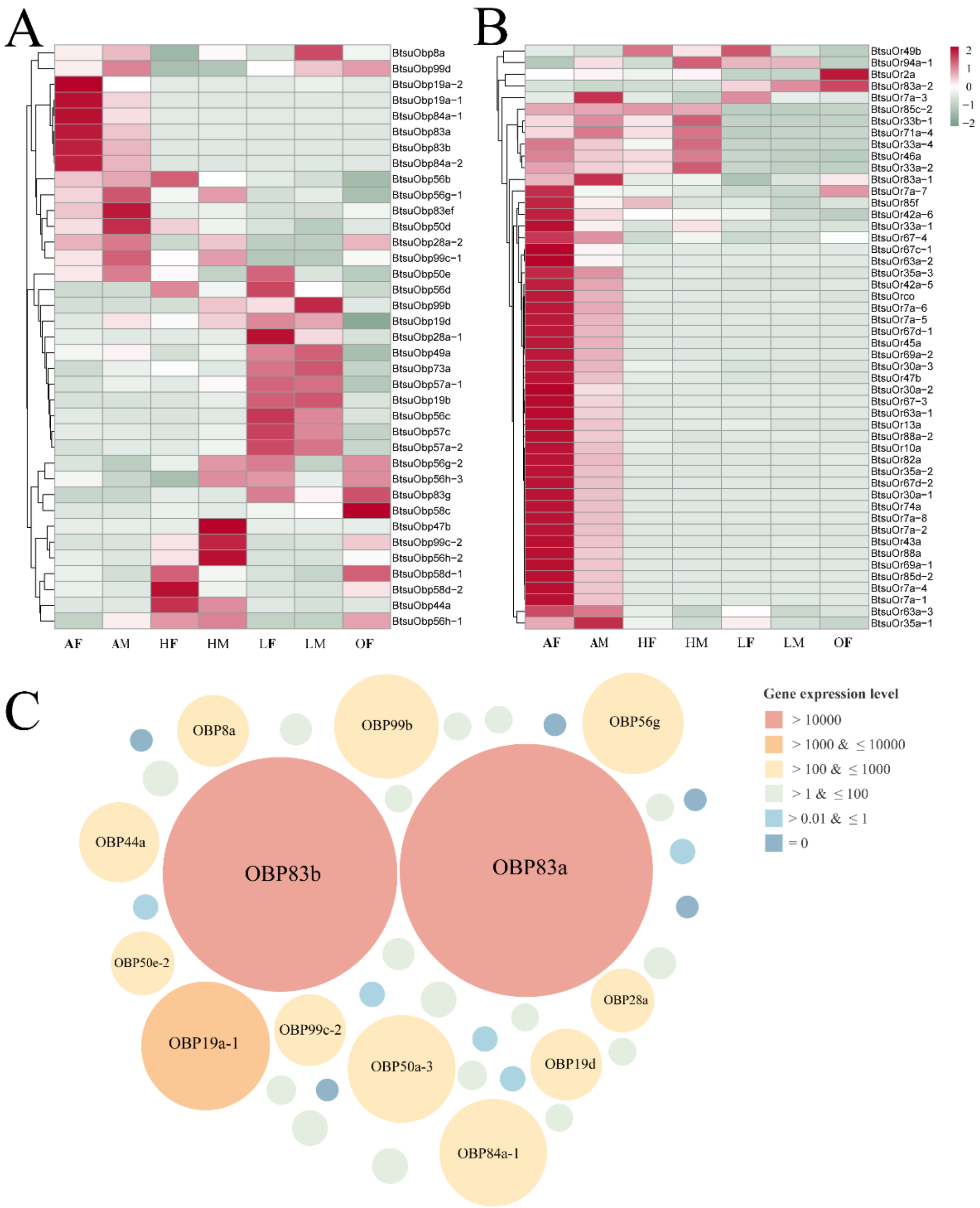

Figure 5: Expression pattern analysis of genes in different tissue (AF: Female adult antennae; AM: Male adult antennae; HF: Female adult head without antennae; HM: Male adult head without antennae; LF: Female adult leg; LM: Male adult leg; OF: Female adult ovipositor). (A) BtsuOBPs in different tissue, (B) BtsuOrs in different

tissue, (C) BtsuOBPs in antennae.

Ligand-binding properties of BtsuOBPs

HS-SPME-GC-MS was employed to identify and quantify volatile compounds emitted by *Maoping Tangerine*, the primary host of *B. tsuneonis*. This method enabled comprehensive profiling of both the composition and concentration of volatiles. A total of 113 volatile compounds were detected in *Maoping Tangerine* (Supplementary Table S9). In the previous study, we completed the identification of volatile compounds from non-host fruits of *B. tsuneonis*, including guava, mango, and apple (Supplementary Fig. S2). To further investigate potential olfactory cues, volatile compounds from *Maoping Tangerine* were compared with those of non-host fruits (Supplementary Fig. S2 and S3). This comparative analysis identified 43 candidate volatiles, including 10 from *Maoping Tangerine* and 33 from non-host plants, as potential ligands for further study. (Supplementary Table S10).

To explore the olfactory mechanisms of *B. tsuneonis*, BtsuOBP83a and BtsuOBP83b, which exhibited high expression levels in the antennae, were selected for functional analysis. Recombinant proteins for these two BtsuOBPs were successfully expressed in vitro, and their purity and molecular size were confirmed via SDS-PAGE (Supplementary Fig. S4). Competitive binding assays using 1-NPN as a fluorescent probe were conducted to assess the binding affinities of BtsuOBPs to 43 selected volatile compounds derived. First, the affinity constants of BtsuOBPs for 1-NPN were determined. Both proteins exhibited characteristic saturation binding curves with 1-NPN, and their Scatchard plots were linear (Supplementary Fig. S5). The dissociation constants (*Kd*) were calculated as 5.48 μM for BtsuOBP83a and 6.92 μM for BtsuOBP83b, confirming 1-NPN as a suitable fluorescent probe for these proteins. Among the 43 tested volatiles, BtsuOBP83a exhibited specific binding affinity to two host-derived volatiles while showing weak binding to non-host-derived volatiles. In contrast, BtsuOBP83b displayed weak binding across all tested compounds. (Fig. 6 and Supplementary Fig. S6; Supplementary Table S11). These findings suggest that BtsuOBP83a may play a crucial role in hosts volatile compounds binding in *B. tsuneonis*.

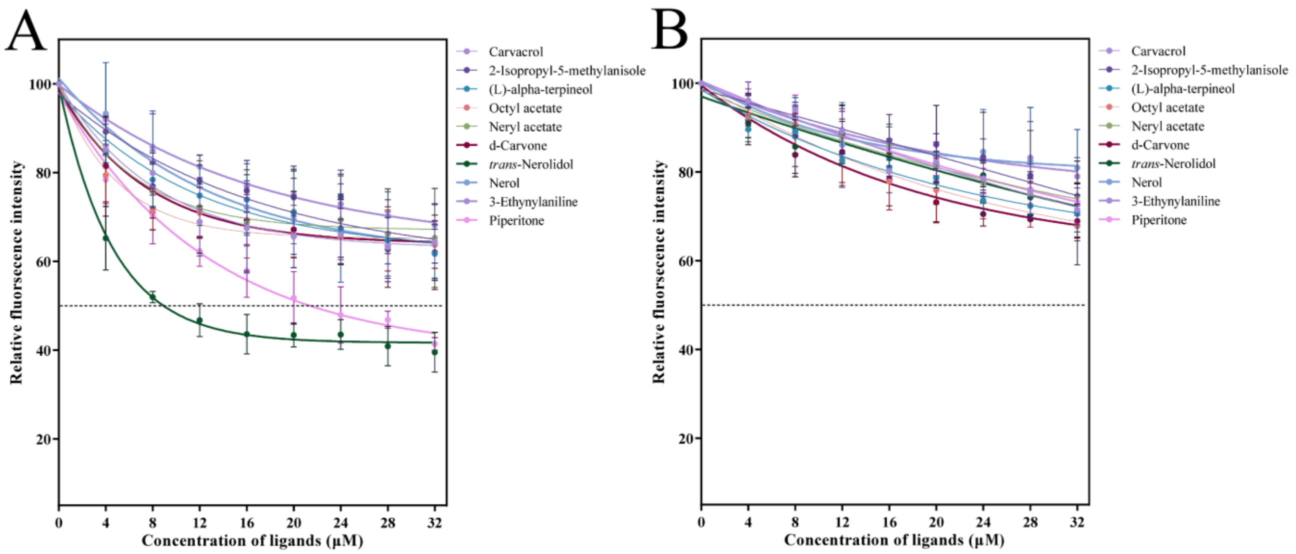

Figure 6: Comparison of binding properties of (A) BtsuOBP83a and (B) BtsuOBP83b with respect to *Maoping Tangerine*.

Structural prediction and molecular docking

The 3D structure of BtsuOBP83a was predicted using AlphaFold 2.0 (Fig. 7A), and the model was assessed as reliable. The predicted structure exhibited six characteristic α-helices and a hydrophobic binding cavity, consistent with the typical structural features of insect odorant-binding proteins (OBPs). Based on the SPME-GC–MS analysis and fluorescence binding assays, *trans*-nerolidol and piperitone were identified as the two major host-derived volatiles that showed strong binding affinity to BtsuOBP83a. To further elucidate the molecular recognition mechanisms underlying these interactions, molecular docking was performed using the predicted OBP structure. The results revealed that BtsuOBP83a possesses a binding cavity capable of interacting with both ligands through hydrophobic interactions and hydrogen bonding (Fig. 7B).

For *trans*-nerolidol, binding was facilitated by hydrophobic interactions with residues PHE22, LEU58, ILE62, LEU76, VAL84, LEU88, TRP114, PHE123, and PRO125, alongside hydrogen bonding with PHE123 (Fig. 7C). Piperitone exhibited hydrophobic interactions with residues LEU76, LEU88, TRP114, TYR122, and PHE123 (Fig. 7D). Molecular docking results indicated low binding energies for *trans*-nerolidol (-6.71 kcal/mol) and piperitone (-6.317 kcal/mol), suggesting strong interactions between BtsuOBP83a and these ligands. These findings highlight BtsuOBP83a as a key OBP involved in host volatile recognition in *B. tsuneonis*.

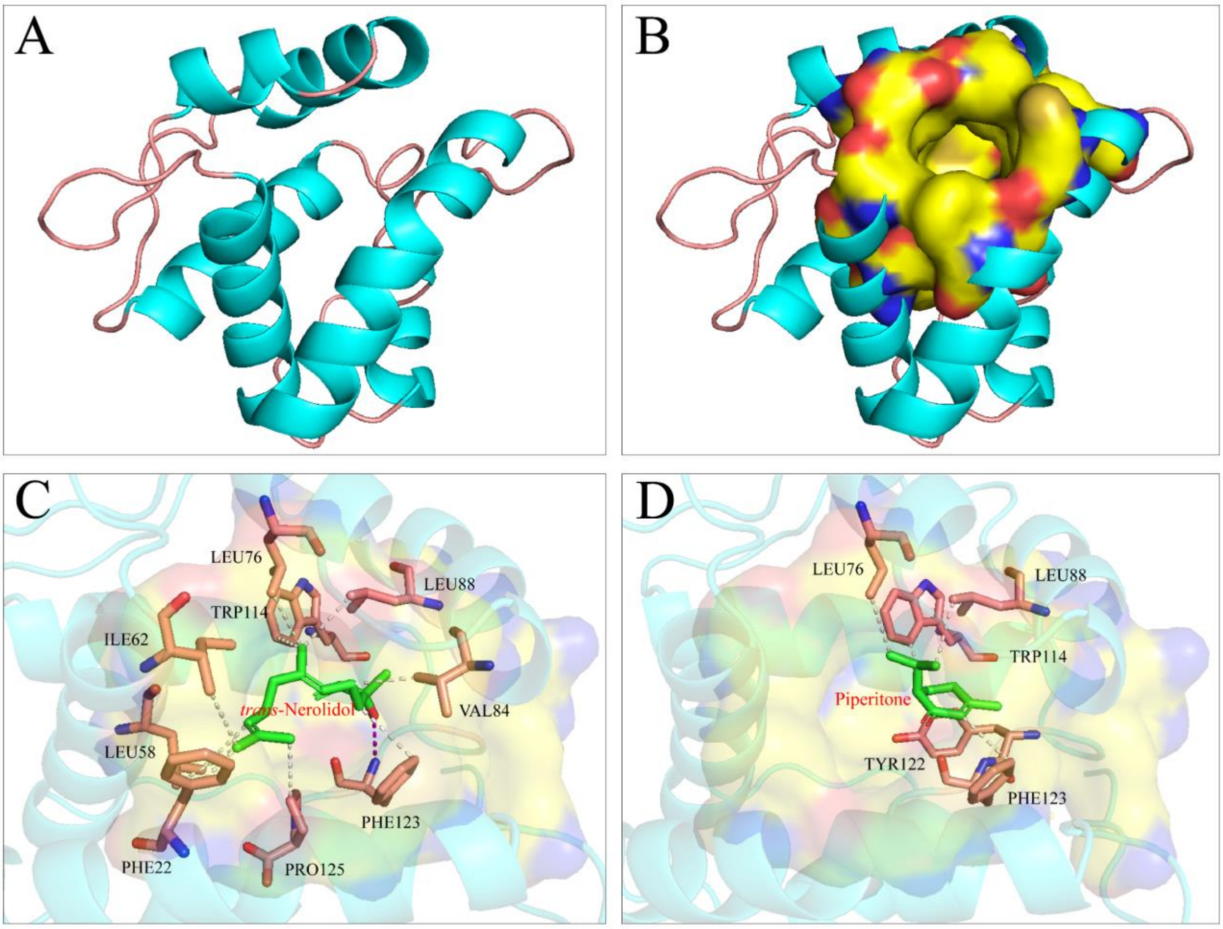

Figure 7: Molecular docking of BtsuOBP83a. (A) Predicted 3D structure, (B) the binding cavity, key residues with respect to (C) *trans*-nerolidol, and (D) piperitone.

To further explore the interactions between key ligands and odorant receptors (ORs) in *B. tsuneonis*, we utilized AlphaFold 2.0 to predict the heteromeric structures of OR-ORco complexes. Molecular docking analyses were performed using the AlphaFold-predicted diagonal heteromeric complex (Fig. 8A and 8B) to assess ligand binding interactions within OR-ORco heterotetramers (Supplementary Table S12). Among the 68 ORs analyzed, BtsuOr7a-6 exhibited the lowest binding energy with *trans*-nerolidol (-7.381 kcal/mol), while BtsuOr7a-4 showed the strongest affinity for piperitone (-6.904 kcal/mol) (Supplementary Fig. S7).

Molecular dynamics simulations

The interaction between *trans*-nerolidol and BtsuOr7a-6 demonstrated remarkable stability and specificity (Fig. 8 and Supplementary Fig. S8). Root mean square deviation (RMSD) analysis showed that the overall protein structure stabilized after 40 ns, with only minor fluctuations. The ligand RMSD remained steady at approximately 0.1 nm, indicating minimal movement within the binding pocket while maintaining a stable interaction. The RMSD of the protein-ligand complex stabilized at 0.55 nm, further supporting the conformational stability of the system. Root mean square fluctuation

(RMSF) analysis revealed minimal fluctuations in the binding site residues, suggesting limited dynamic behavior in the binding pocket, while non-binding regions exhibited greater fluctuations without affecting overall binding stability. MM/PBSA calculations estimated a binding free energy (ΔGMMGBSA) of -37.11 kcal/mol, indicating high thermodynamic stability. The interaction was primarily driven by van der Waals forces (ΔVDWAALS = -38.93 kcal/mol) and nonpolar solvation energy (ΔESURF = -5.61 kcal/mol). Solvent-accessible surface area (SASA) analysis showed a decrease from 900 nm^2 to below 800 nm^2 upon ligand binding, suggesting that *trans*-nerolidol was stably embedded within the binding site, further enhancing the specificity of the interaction.

The binding of piperitone to BtsuOr7a-4 exhibited high stability and adaptability (Fig. 8 and Supplementary Fig. S9). RMSD analysis indicated that the overall protein structure stabilized after 20 ns, with a slight increase in complex RMSD after 80 ns. The ligand RMSD remained consistently around 0.04 nm, reflecting a highly stable binding position. Dynamic evaluations of the binding site showed minimal fluctuations in the core binding residues, while non-binding regions exhibited greater flexibility without compromising overall binding stability. MM/PBSA calculations estimated a binding free energy (ΔGMMGBSA) of -23.56 kcal/mol, with van der Waals forces (ΔVDWAALS = -26.55 kcal/mol) as the primary driving force. SASA analysis revealed a decrease from 860 nm^2 to 750 nm^2, indicating that piperitone was securely embedded within the receptor.

Comprehensive analysis suggests that the binding of BtsuORs to *trans*-nerolidol and piperitone is primarily driven by hydrophobic interactions. Core binding pocket residues, such as VAL197 in BtsuOr7a-6 and LEU195 in BtsuOr7a-4, play crucial roles in stabilizing ligand interactions (Fig. 8). Among the two receptors, BtsuOr7a-6 exhibited the strongest binding affinity with *trans*-nerolidol (ΔGMMGBSA = -37.11 kcal/mol), primarily driven by van der Waals interactions. The larger binding pocket of BtsuOr7a-6 likely provides greater accommodation capacity, enhancing its adaptability. Conversely, BtsuOr7a-4 demonstrated the highest binding stability with piperitone, with ligand RMSD consistently below 0.05 nm and minimal fluctuations in the binding site, suggesting that piperitone may serve as a specific ligand for BtsuOr7a-4.

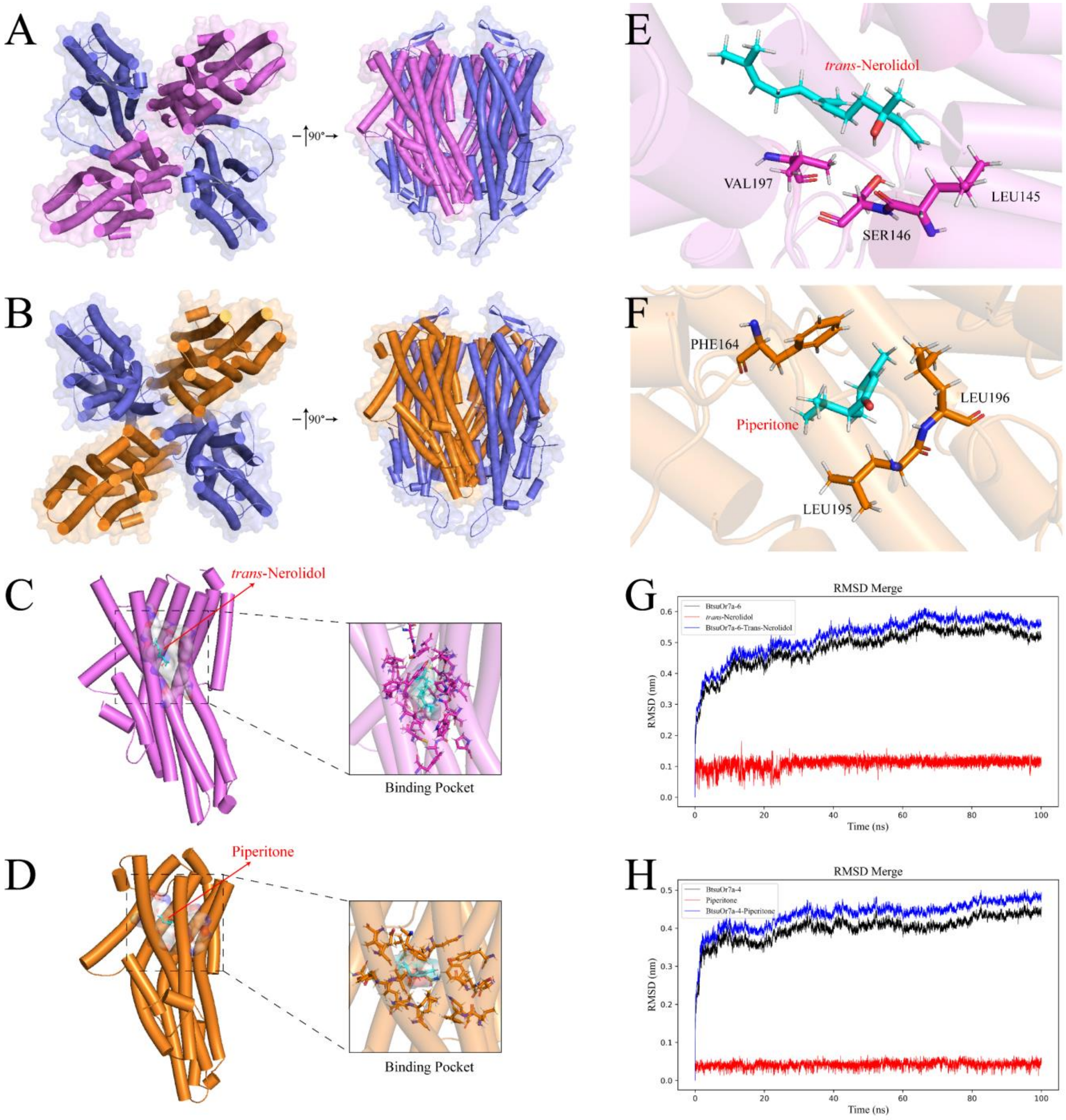

Figure 8: Molecular dynamics simulations of substrate-bound BtsuORs. Representative structure of the heteromeric structures of (A) BtsuOR7a-6, (B) BtsuOR7a-4 in complex with BtsuORco in a stoichiometry of two ORs and two ORcos predicted, Close-up view of the docking structure of BtsuORs in complex with (C) *trans*-nerolidol, (D) piperitone , Structural snapshots to show the interactions between BtsuORs and (E) *trans*-nerolidol, (F) piperitone, RMSD analysis of BtsuORs in complex with (G) *trans*-nerolidol, (H) piperitone.

Discussion

Genome offers a foundation for studying host specialization and adaptation

The chromosome-level genome assembly of *B. tsuneonis* provides a crucial foundation for understanding the genetic basis of its host specialization and ecological adaptation. Compared to other

Bactrocera species, *B. tsuneonis* exhibits a relatively small genome size (339 Mb) with a lower proportion of repetitive elements (24.17%). This is consistent with previous studies showing that genome size can be influenced by the proportion of transposable elements and repeat sequences, which vary across insect species depending on their evolutionary history and ecological adaptations [103,104,105]. The high contig N50 (11.21 Mb) and scaffold N50 (59.93 Mb) values indicate a well-assembled and contiguous genome, facilitating downstream functional and comparative genomic analyses.

A well-assembled genome is essential for understanding the genetic mechanisms underlying insect specialization. Previous studies on *B. dorsalis* and *B. correcta* have shown that genome structure plays a role in determining ecological plasticity and host adaptability [106,107]. The high completeness (99.2% BUSCO) of *B. tsuneonis* further supports the reliability of this assembly, and accurate gene annotation and functional analysis have been conducted. The availability of this reference genome serves as a valuable resource for investigating the molecular basis of host selection, ecological divergence, and evolutionary trajectories within *Bactrocera* species.

Gene contractions in olfaction highlight adaptation to host

Comparative genomic analysis revealed significant contractions in the chemosensory gene families of *B. tsuneonis,* particularly in OBPs and ORs. These gene families play a critical role in insect olfactory perception and host recognition, facilitating the detection of plant volatiles and pheromones [108,109]. In the genome of *B. tsuneonis*, these gene families have undergone a significant reduction compared to polyphagous *Bactrocera* species (Fig. 4), a trend that has also been observed in other oligophagous insects [110,111]. This contraction may enhance the ability of *B. tsuneonis* to accurately identify and select its host, thereby improving its ecological adaptability. We hypothesize that the contraction of OR genes may play a vital role in population establishment and the rapid spread of invasive species, facilitating efficient host localization and optimizing foraging and reproductive strategies.

Despite the reduction in chemosensory genes, detoxification-related gene families, including CYP450s, ABCs, and GSTs, exhibited expansion in *B. tsuneonis*. During our gene family analysis, an abnormal increase in the number of detoxification-related genes was observed in *B. minax*. Therefore, in this study, we did not include a comparison of detoxification-related genes between *B. minax* and *B. tsuneonis*. These gene families are essential for metabolizing plant secondary metabolites and insecticides [112]. The expansion of detoxification genes in *B. tsuneonis* may reflect an evolutionary adaptation to citrus-derived allelochemicals, providing enhanced metabolic resistance to host-specific phytotoxins.

Tissue-specific expression of OBPs and ORs highlights their roles in olfactory perception

Genomic studies have revealed that the olfactory system of *B. tsuneonis* consists of 39 OBPs and 68 ORs, highlighting the complexity of its chemosensory system [106,107]. To further investigate the roles of these genes in olfactory function and oviposition preference, this study analyzed their transcriptional levels across various tissues, including the antennae, head (excluding antennae), legs, and ovipositor. The results indicated distinct expression patterns of OBPs and ORs in different body parts of *B. tsuneonis*, emphasizing their specialized roles in olfactory perception (Fig. 3). OBPs exhibited broad expressions, with significant enrichment in the antennae, ovipositor, and legs. 13 *BtsuOBPs* were highly expressed in the antennae, reinforcing the antennae's role as the primary olfactory organ for detecting volatile compounds. These OBPs are likely involved in binding and transporting environmental volatiles to ORs, thereby initiating olfactory signal transduction cascades [113,114]. Additionally, several *BtsuOBPs* were expressed in the legs, suggesting potential roles beyond olfactory perception. For instance, *BdorOBP28a-2* expression in the legs has been implicated in *B. dorsalis* resistance to malathion [115].

In contrast, OR genes were predominantly expressed in the antennae, consistent with their function in olfactory signal transduction [116]. Previous studies have demonstrated that ORs are essential for recognizing specific host-associated volatiles and play a critical role in mediating insect behavior [14,117]. The limited expression of ORs in non-olfactory tissues suggests that their primary function is in odor recognition. Interestingly, both OBPs and ORs were detected in the ovipositor, suggesting a potential role in oviposition site selection. Olfactory genes are expressed in reproductive tissues, likely contributing to host recognition during oviposition [118]. The high expression levels of OBPs and ORs in the antennae particularly emphasize their importance in volatile perception, aiding fruit flies in recognizing host fruits and potentially detecting ecological risks or competitive pressures associated with specific odors.

BtsuOBP83a mediates host selection by binding key volatiles

Transcriptomic analysis revealed that *BtsuOBP83a* and *BtsuOBP83b* exhibited the highest expression levels in female antennae, underscoring their critical role in olfactory perception (Fig. 3C). To investigate their function in host volatile recognition, we assessed the binding ability of these two highly expressed OBPs to 10 host-derived volatile compounds and 33 non-host-derived volatile compounds. Fluorescence competitive binding assays demonstrated that *BtsuOBP83a* selectively binds to two host-specific volatiles, *trans*-nerolidol and piperitone, suggesting that these compounds serve as key olfactory cues for *B. tsuneonis*. OBPs play a crucial role in odorant transport and are essential for facilitating ligand-receptor interactions within the insect olfactory system [18,21]. The strong binding affinity observed in this study supports the hypothesis that *BtsuOBP83a* serves as a key mediator in host odor recognition for *B. tsuneonis*, playing an important ecological role in host

selection and reproductive behavior. Compared to polyphagous species, *B. tsuneonis* has fewer OBPs, which may indicate an evolutionary trade-off that prioritizes specificity in odor detection over diversity.

According to previous studies, host preference in both specialists and generalists is primarily influenced by visual and olfactory cues [110]. BtsuOBP83a exhibited specific binding affinity to two host-derived volatiles while showing weak binding to non-host-derived volatiles, further supporting the idea that specialist insects rely on an olfactory system to accurately detect and respond to host-specific chemical cues. However, the preference for *Maoping Tangerine* as the primary host may limit the adaptability of *B. tsuneonis*, making it more vulnerable to environmental fluctuations and changes in host availability. To further elucidate the molecular recognition mechanisms underlying these preferences, we conducted molecular docking analyses to examine the interactions between *BtsuOBP83a* and several host volatiles. The lower binding energies observed in these analyses indicate stronger ligand-protein interactions, supporting the fluorescence binding assay results and validating the structural basis of these interactions. Future behavioral assays, such as Y-tube olfactometer and oviposition preference tests, will be valuable for linking OBP – ligand binding profiles to actual host-seeking behaviors, and for confirming the ecological relevance of key volatiles identified in this study.

BtsuOr7a-6 and BtsuOr7a-4 mediate host recognition by detecting key volatiles

To further investigate the interactions between the 2 volatile compounds and ORs in *B. tsuneonis*, we utilized AlphaFold2 structural prediction, molecular docking, and MD simulations. Based on AlphaFold2 predictions, OR and ORco are hypothesized to assemble into a heterotetrameric structure, $(OR)_2$–$(ORco)_2$, with two possible structural arrangements: adjacent and diagonal configurations [119]. In this study, 10 models were generated for each configuration, with the majority adopting a diagonal arrangement, suggesting that this conformation may be more stable in fruit flies. Molecular docking experiments identified *BtsuOr7a-6* and *BtsuOr7a-4* as the key receptors with the lowest binding free energy for *trans*-nerolidol and piperitone, respectively, among the 68 OR candidates. These findings suggest that these ORs are the primary receptors involved in detecting these volatiles. Previous studies have demonstrated that the OR7a family is essential for the detection of chemical signals commonly recognized by *D. melanogaster* [120]. ORs are key determinants of odor coding, and their ligand specificity directly influences insect behavior [121,122]. The low binding energy values obtained from molecular docking indicate that these ORs have evolved to detect specific citrus volatiles with high sensitivity, reinforcing their role in host location.

MD simulations further validated the binding stability of the two volatiles with their respective ORs. Core residues within the binding pocket, such as VAL197 and LEU195, provided the primary driving

force through hydrophobic interactions. These results reveal the high adaptability of the OR binding pocket for hydrophobic volatiles and the crucial role of polar residues in recognizing complex ligands. The highly stable binding of *BtsuOr7a-6* to *trans*-nerolidol suggests that this receptor plays a central role in mediating host attraction, while the specificity of *BtsuOr7a-4* for piperitone indicates its involvement in detecting additional host-related cues. These findings support the idea that ORs drive host localization and reproductive behaviors in fruit flies by recognizing host-specific volatiles [123]. Additionally, the contraction of OR genes in *B. tsuneonis* reflects an adaptation toward detecting a narrow yet ecologically relevant set of host volatiles, further supporting its olfactory specialization. To further confirm the ligand-receptor relationships predicted here, future work will employ heterologous expression systems to functionally characterize these ORs and directly validate their roles in odorant perception.

Oligophagy and its evolutionary significance in insects

Oligophagy, or the specialization of insects on a narrow range of host plants, represents a distinct evolutionary strategy that contrasts with polyphagy, where insects feed on a broad spectrum of plant species. Oligophagous insects, such as *B. tsuneonis*, exhibit strong host specificity, often displaying precise adaptations in their chemoreception, detoxification mechanisms, and behavioral strategies. The evolutionary drivers and consequences of oligophagy have been widely studied in insect ecology and evolutionary biology, highlighting the trade-offs between host specialization and ecological flexibility [124,125]. One of the key adaptations associated with oligophagy is the reduction in the number of olfactory-related genes, as specialized herbivorous insects typically evolve a more selective olfactory repertoire. [110,111]. This mechanism provides an advantage in locating suitable host plants within complex environments but comes at the cost of reduced adaptability to new or alternative hosts. In this study, the contraction of OR genes and the functional analysis of OBPs in *B. tsuneonis* further support this pattern, as they prioritize sensitivity to a limited set of ecologically relevant chemical signals.

Despite the ecological advantages of host specialization, oligophagy imposes inherent constraints. Oligophagous insects are more vulnerable to fluctuations in host availability, environmental changes, and habitat disturbances, making them potentially more susceptible to population declines under unfavorable conditions [126,127]. Additionally, their evolutionary flexibility is restricted, as they are less capable of shifting to new hosts compared to polyphagous species. However, in stable environments where host plants are abundant, oligophagy can be a highly successful strategy, allowing insects to avoid interspecific competition and optimize feeding efficiency [128]. Overall, the evolution of oligophagy reflects a trade-off between ecological specialization and adaptability. Insect species that exhibit oligophagy, such as *B. tsuneonis*, have fine-tuned their olfactory and

detoxification systems to maximize efficiency in host detection and utilization. Future research should continue to explore the genetic and ecological mechanisms underpinning oligophagy, particularly in pest species, to improve our understanding of host-insect interactions and inform pest management strategies.

Conclusions

This study presents the first high-quality chromosome-level genome assembly of *B. tsuneonis*, revealing key genomic adaptations underlying its host specificity. Comparative genomic analysis identified a significant contraction in chemosensory gene families, particularly OBPs and ORs, consistent with its oligophagous nature. Functional assays confirmed that *BtsuOBP83a* binds strongly to host volatiles *trans*-nerolidol and piperitone, while *BtsuOr7a-6* and *BtsuOr7a-4* serve as key receptors for host odor recognition. These interactions, primarily driven by hydrophobic forces, reveal the structural basis of host recognition in *B. tsuneonis*. This study provides insights into the molecular mechanisms of host selection in *B. tsuneonis* and contributes valuable genomic evidence on olfactory adaptation in oligophagous insects. Future research should validate these key genes in vivo and explore behavior-based pest control strategies for more precise and sustainable management of fruit flies.

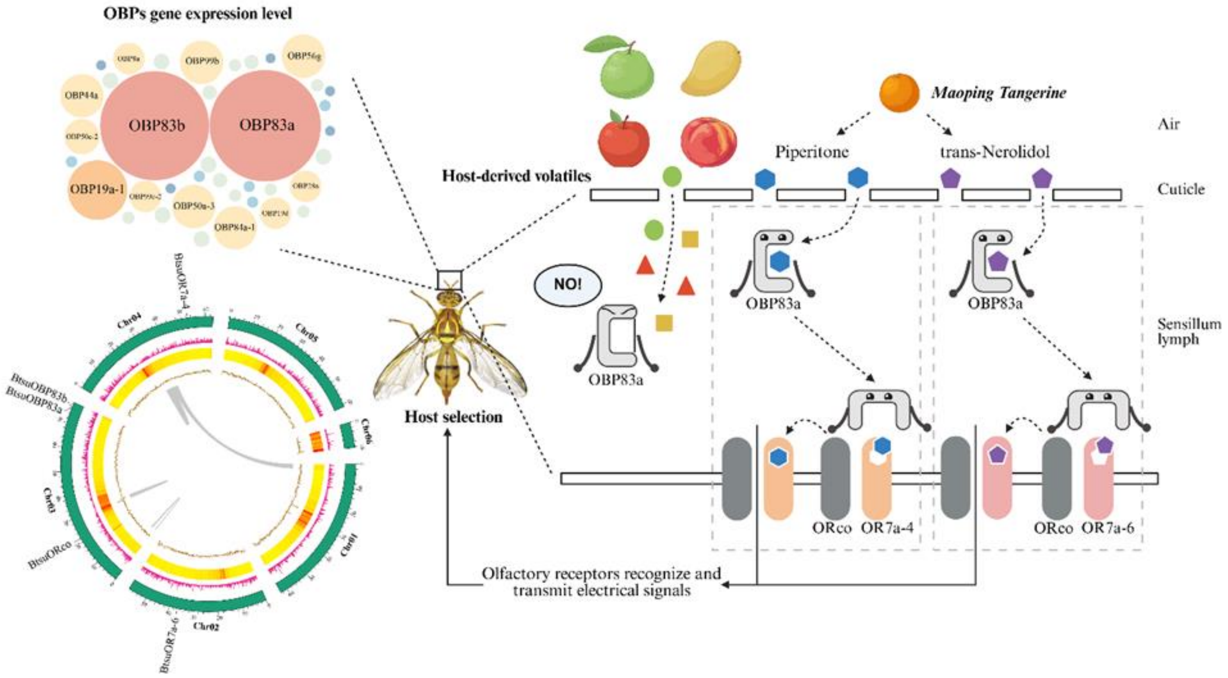

Figure 9: Schematic diagram of genome assembly, OBP expression level in antennae, and olfactory protein recognition mechanism of host volatiles in *B. tsuneonis*.

Additional Files

Supplementary Fig. S1. Correlation between gene expression levels from RNA-seq and RT-qPCR.

Supplementary Fig. S2. Gas chromatograph-mass spectrometry analyses (GC-MS) of host fruits (guava, mango, and apple from the previous study).

Supplementary Fig. S3. Gas chromatograph-mass spectrometry (GC-MS) analyses without fruit.

Supplementary Fig. S4. SDS-PAGE analysis of recombinant target proteins. (M: Molecular Weight Marker; A: Protein Sample After Enterokinase Cleavage; B: Target Protein After Enterokinase Cleavage; C: PBS Elution Fraction; D: 500 mM Imidazole Elution Fraction).

Supplementary Fig. S5. Binding curves and Scatchard plots of the fluorescence probe 1-NPN to BtsuOBPs.

Supplementary Fig. S6. Comparison of binding properties of (A) BtsuOBP83a and (B) BtsuOBP83b with respect to guava, mango, apple.

Supplementary Fig. S7. Key residues of BtsuORs with respect to trans-nerolidol and piperitone.

Supplementary Fig. S8. RMSF and SASA analysis of BtsuOR7a-6 in complex with trans-nerolidol.

Supplementary Fig. S9. RMSF and SASA analysis of BtsuOR7a-4 in complex with piperitone.

Supplementary Table S1. Primers used in this study.

Supplementary Table S2. Restriction enzymes used in this study.

Supplementary Table S3. Statistics of sequencing data of *Bactrocera tsuneonis* genome.

Supplementary Table S4. Completeness of *Bactrocera tsuneonis* genome assembly and annotation evaluated by BUSCO based on insecta_odb10 database.

Supplementary Table S5. Statistics for repeat elements in the genome of *Bactrocera tsuneonis*.

Supplementary Table S6. Functional annotation statistics of *Bactrocera tsuneonis* genome.

Supplementary Table S7. Information regarding gene family clustering in the 17 species used for comparative analyses.

Supplementary Table S8. Statistics on detoxification, heatshock protein (HSP), and sensing-related genes across *Bactrocera* insects and other insects.

Supplementary Table S9. GC-MS Analysis of VOCs in *Maoping Tangerine*.

Supplementary Table S10. Chemical compounds used in this study.

Supplementary Table S11. Binding affinities of all tested ligands to BtsuOBPs.

Supplementary Table S12. BtsuOrs with ligands interaction energy calculated by molecular docking.

Data Availability

The genome sequence had been deposited at the National Center for Biotechnology Information (NCBI), under the accession number of JBKBCS000000000. The NCBI BioProject accession number is PRJNA1182520. The transcriptome sequence had been deposited at the National Center for

Biotechnology Information (NCBI), under the BioProject accession number is PRJNA1246368. All additional supporting data are available in the GigaScience repository, GigaDB [129].

Abbreviations

1-NPN: N-phenyl-1-naphthylamine; ABC: ATP-binding cassette transporter; BLAST: Basic Local Alignment Search Tool; BUSCO: Benchmarking Universal Single-Copy Orthologs; CCE: Carboxyl/cholinesterase; CCS: Circular consensus sequence; CDS: Coding sequence; COG: Clusters of Orthologous Genes; CSP: Chemosensory protein; CYP450: Cytochrome P450 monooxygenase; EI: Electron ionization; GC-MS: Gas chromatography coupled with mass spectrometry; GFF: General feature format; GO: Gene Ontology; GR: Gustatory receptor; GST: Glutathione S-transferase; Hi-C: High-resolution chromosome conformation capture; HMM: Hidden Markov model; HSP: Heat shock protein; HS-SPME: Headspace Solid Phase Micro-extraction; IPTG: Isopropyl-β-D-thiogalactopyranoside; IR: Ionotropic receptor; KEGG: Kyoto Encyclopedia of Genes and Genomes; LINE: Long interspersed nuclear element; LTR: Long terminal repeat; MD: Molecular dynamics; MMGBSA: Molecular mechanics generalized Born surface area; Mya: Million years ago; NCBI: National Center for Biotechnology Information; OBP: Odorant-binding protein; OD: Optical density; OR: Odorant receptor; ORco: Odorant receptor co-receptor; ORF: Open reading frames; PCR: Polymerase Chain Reaction; RMSD: Root-mean-square deviation; RMSF: Root-mean-square fluctuation; RNA-seq: RNA sequencing; SASA: Solvent-accessible surface area; SDS-PAGE: Sodium dodecyl sulfate-polyacrylamide gel electrophoresis; SignalP: Signal peptides; SNMP: Sensory neuron membrane protein; UGT: UDP-glucuronosyltransferase; UTR: Untranslated regions; VDWAALS: Van der Waals forces.

Competing Interests

The authors declare no competing interests.

Funding

This work is supported by National Natural Science Foundation of China (32202288) and National Key R&D Program of China (2022YFC2601500).

Authors' Contributions

Tengda Guo, Yujia Qin and Zhihong Li conceived the project; Tengda Guo, Wenzhao Yang and Yuan Zhang performed the experiments; Tengda Guo and Weisong Li performed the bioinformatic analyses; Tengda Guo, Yujia Qin and Zhihong Li evaluated the results; Tengda Guo wrote the manuscript. Yujia Qin and Zhihong Li improved and revised the manuscript. All authors read and approved of the final manuscript.

Acknowledgments

We thank the 2115 Talent Development Program of China Agricultural University.

References

[1] Mochizuki M, Arai T, Mishiro K, Okazaki Y, Higashiura Y. Control of the Japanese orange fly, *Bactrocera tsuneonis* (Diptera: Tephritidae), through several preharvest management practices: establishment of a phytosanitary measure for citrus fruits for export. Appl Entomol Zool 2024;59:317–29. https://doi.org/10.1007/s13355-024-00881-w.

[2] Opadith P, Iwamoto S, Narahara M, Okazaki Y, Higashiura Y, Otake J, et al. Development of microsatellite markers for the Japanese orange fly, *Bactrocera tsuneonis* (Diptera: Tephritidae). Appl Entomol Zool 2022;57:283–8. https://doi.org/10.1007/s13355-022-00783-9.

[3] Ono H, Ota S, Kanno S, Nomura Y, Narahara M, Okazaki Y. Detection of environmental DNA of the Japanese orange fly, *Bactrocera tsuneonis* (Diptera: Tephritidae), from immature mandarin orange fruits. Appl Entomol Zoolog 2025;60:45–51. https://doi.org/10.1007/s13355-024-00890-9.

[4] Zhang Y, Feng S, Zeng Y, Ning H, Liu L, Zhao Z, et al. The first complete mitochondrial genome of *Bactrocera tsuneonis* (Miyake) (Diptera: Tephritidae) by next-generation sequencing and its phylogenetic implications. Int J Biol Macromol 2018;118:1229–37. https://doi.org/10.1016/j.ijbiomac.2018.06.099.

[5] Wu GA, Terol J, Ibanez V, López-García A, Pérez-Román E, Borredá C, et al. Genomics of the origin and evolution of Citrus. Nature 2018;554:311–6. https://doi.org/10.1038/nature25447.

[6] Li F, Zhao X, Li M, He K, Huang C, Zhou Y, et al. Insect genomes: progress and challenges. Insect Mol Biol 2019;28:739–58. https://doi.org/10.1111/imb.12599.

[7] Li F, Wang X, Zhou X. The Genomics Revolution Drives a New Era in Entomology. Annu Rev Entomol 2025;70:379–400. https://doi.org/10.1146/annurev-ento-013024-013420.

[8] Vargas RI, Pinero JC, Leblanc L. An Overview of Pest Species of *Bactrocera* Fruit Flies (Diptera: Tephritidae) and the Integration of Biopesticides with Other Biological Approaches for Their Management with a Focus on the Pacific Region. Insects 2015;6:297–318. https://doi.org/10.3390/insects6020297.

[9] Wu Z, Cui Y, Ma J, Qu M, Lin J. Analyses of chemosensory genes provide insight into the evolution of behavioral differences to phytochemicals in *Bactrocera* species. Mol Phylogenet Evol 2020;151:106858. https://doi.org/10.1016/j.ympev.2020.106858.

[10] NCBI. https://www.ncbi.nlm.nih.gov/datasets/genome. Accessed 1 April 2025.

[11] Djambazian H, Chen S, Bérubé P, Nikolouli K, Grigoriou M, Rallis D, et al. Genome assembly of five Tephritid species for the enhancement of the Sterile Insect Technique. bioRxiv 2025.

https://doi.org/10.1101/2025.09.15.670340
[12] Zhao Z, Carey JR, Li Z. The Global Epidemic of Bactrocera Pests: Mixed-Species Invasions and Risk Assessment. Annu Rev Entomol 2024;69:219–37. https://doi.org/10.1146/annurev-ento-012723-102658.
[13] Liu Z, Xie Q, Guo H, Xu W, Wang J. An odorant binding protein mediates *Bactrocera dorsalis* olfactory sensitivity to host plant volatiles and male attractant compounds. Int J Biol Macromol 2022;219:538–44. https://doi.org/10.1016/j.ijbiomac.2022.07.198.
[14] Zhang Y, Liu W, Luo Z, Yuan J, Wuyun Q, Zhang P, et al. Odorant Receptor BdorOR49b Mediates Oviposition and Attraction Behavior of *Bactrocera dorsalis* to Benzothiazole. J Agric Food Chem 2024;72:7784–93. https://doi.org/10.1021/acs.jafc.3c09791.
[15] Vogt RG, Rogers ME, Franco MD, Sun M. A comparative study of odorant binding protein genes:: differential expression of the PBP1-GOBP2 gene cluster in *Manduca sexta* (Lepidoptera) and the organization of OBP genes in *Drosophila melanogaster* (Diptera). J Exp Biol 2002;205:719–44. https://doi.org/10.1242/jeb.205.6.719.
[16] Pelosi P, Maida R. Odorant-Binding Proteins in Insects. Comp Biochem Physiol B-Biochem Molec Biol 1995;111:503–14. https://doi.org/10.1016/0305-0491(95)00019-5.
[17] Sandler BH, Nikonova L, Leal WS, Clardy J. Sexual attraction in the silkworm moth: structure of the pheromone-binding-protein-bombykol complex. Chem Biol 2000;7:143–51. https://doi.org/10.1016/S1074-5521(00)00078-8.
[18] Leal WS, Odorant Reception in Insects: Roles of Receptors, Binding Proteins, and Degrading Enzymes. Annu Rev Entomol 2013;58:373–91. https://doi.org/10.1146/annurev-ento-120811-153635.
[19] Pelosi P, Zhou J, Ban LP, Calvello M. Soluble proteins in insect chemical communication. Cell Mol Life Sci 2006;63:1658–76. https://doi.org/10.1007/s00018-005-5607-0.
[20] Zhou J, Robertson G, He X, Dufour S, Hooper AM, Pickett JA, et al. Characterisation of Bombyx mori Odorant-binding Proteins Reveals that a General Odorant-binding Protein Discriminates Between Sex Pheromone Components. J Mol Biol 2009;389:529–45. https://doi.org/10.1016/j.jmb.2009.04.015.
[21] Brito NF, Moreira MF, Melo ACA. A look inside odorant-binding proteins in insect chemoreception. J Insect Physiol 2016;95:51–65. https://doi.org/10.1016/j.jinsphys.2016.09.008.
[22] Sachse S, Krieger J. Olfaction in insects. e-Neuroforum 2011;2:49–60. https://doi.org/10.1007/s13295-011-0020-7
[23] Wicher D, Miazzi F. Functional properties of insect olfactory receptors: ionotropic receptors and

odorant receptors. Cell Tissue Res 2021;383:7–19. https://doi.org/10.1007/s00441-020-03363-x.
[24] Vosshall LB, Hansson BS. A Unified Nomenclature System for the Insect Olfactory Coreceptor. Chem Senses 2011;36:497–8. https://doi.org/10.1093/chemse/bjr022.
[25] Ha TS, Smith DP. Odorant and pheromone receptors in insects. Front Cell Neurosci 2009;3:10. https://doi.org/10.3389/neuro.03.010.2009.
[26] Reed RR. After the holy grail: Establishing a molecular basis for mammalian olfaction. Cell 2004;116:329–36. https://doi.org/10.1016/S0092-8674(04)00047-9.
[27] Butterwick JA, del Marmol J, Kim KH, Kahlson MA, Rogow JA, Walz T, et al. Cryo-EM structure of the insect olfactory receptor Orco. Nature 2018;560:447–52. https://doi.org/10.1038/s41586-018-0420-8.
[28] del Marmol J, Yedlin MA, Ruta V. The structural basis of odorant recognition in insect olfactory receptors. Nature 2021;597:126–31. https://doi.org/10.1038/s41586-021-03794-8.
[29] Wang Y, Qiu L, Wang B, Guan Z, Dong Z, Zhang J, et al. Structural basis for odorant recognition of the insect odorant receptor OR-Orco heterocomplex. Science 2024;384:1453–60. https://doi.org/10.1126/science.adn6881.
[30] Jin Z, Wei Z. Molecular simulation for food protein-ligand interactions: A comprehensive review on principles, current applications, and emerging trends, Compr. Rev. Food. Sci. Food Saf. 23 (2024) 1–29. https://doi.org/10.1111/1541-4337.13280.
[31] Karplus M. Molecular dynamics simulations of biomolecules, Acc. Chem. Res. 35 (2002) 321–323. https://doi.org/10.1021/ar020082r.
[32] Coleman JA, Green EM, Gouaux E. X-ray structures and mechanism of the human serotonin transporter, Nature 532 (2016) 334–339. https://doi.org/10.1038/nature17629.
[33] Zheng L, Zhang Y, Yang W, Zeng Y, Jiang F, Qin Y, et al. New Species-Specific Primers for Molecular Diagnosis of *Bactrocera minax* and *Bactrocera tsuneonis* (Diptera: Tephritidae) in China Based on DNA Barcodes. Insects 2019;10:447. https://doi.org/10.3390/insects10120447.
[34] Marcais G, Kingsford C. A fast, lock-free approach for efficient parallel counting of occurrences of k-mers. Bioinformatics 2011;27:764–70. https://doi.org/10.1093/bioinformatics/btr011.
[35] Vurture GW, Sedlazeck FJ, Nattestad M, Underwood CJ, Fang H, Gurtowski J, et al. GenomeScope: fast reference-free genome profiling from short reads. Bioinformatics 2017;33:2202–4. https://doi.org/10.1093/bioinformatics/btx153.
[36] Cheng H, Concepcion GT, Feng X, Zhang H, Li H. Haplotype-resolved de novo assembly using phased assembly graphs with hifiasm. Nat Methods 2021;18:170–5.

https://doi.org/10.1038/s41592-020-01056-5.
[37] Guan D, McCarthy SA, Wood J, Howe K, Wang Y, Durbin R. Identifying and removing haplotypic duplication in primary genome assemblies. Bioinformatics 2020;36:2896–8. https://doi.org/10.1093/bioinformatics/btaa025.
[38] Li H, Durbin R. Fast and accurate short read alignment with Burrows-Wheeler transform. Bioinformatics 2009;25:1754–60. https://doi.org/10.1093/bioinformatics/btp324.
[39] Dudchenko O, Batra SS, Omer AD, et al. De novo assembly of the *Aedes aegypti* genome using Hi-C yields chromosome-length scaffolds. Science 2017;356:92–5. https://doi.org/10.1126/science.aal3327.
[40] Durand NC, Shamim MS, Machol I, et al. Juicer provides a oneclick system for analyzing loop-resolution Hi-C experiments. Cell Syst 2016;3:95–8. https://doi.org/10.1016/j.cels.2016.07.002.
[41] Robinson JT, Turner D, Durand NC, Thorvaldsdóttir H, Mesirov JP, Aiden EL. Juicebox.js Provides a Cloud-Based Visualization System for Hi-C Data. Cell Systems 2018;6: 256-258. https://doi.org/10.1016/j.cels.2018.01.001
[42] Chen N. Using RepeatMasker to identify repetitive elements in genomic sequences. Curr Protoc Bioinformatics 2004;5:4–10.https://doi.org/10.1002/0471250953.bi0410s25.
[43] Storer J, Hubley R, Rosen J, Wheeler TJ, Smit AF. The Dfam community resource of transposable element families, sequence models, and genome annotations. Mob DNA 2021;12:2. https://doi.org/10.1186/s13100-020-00230-y.
[44] Jurka J, Kapitonov VV, Pavlicek A, et al. Repbase Update, a database of eukaryotic repetitive elements. Cytogenet Genome Res 2005;110:462–7. https://doi.org/10.1159/000084979.
[45] Flynn JM, Hubley R, Goubert C, et al. RepeatModeler2 for automated genomic discovery of transposable element families. Proc Natl Acad Sci USA 2020;117:9451–7. https://doi.org/10.1073/pnas.1921046117.
[46] Ou S, Jiang N. LTR_FINDER_parallel: parallelization of LTR_FINDER enabling rapid identification of long terminal repeat retrotransposons. Mobile DNA 2019;10:48. https://doi.org/10.1186/s13100-019-0193-0.
[47] Ou S, Jiang N. LTR_retriever: a highly accurate and sensitive program for identification of long terminal repeat retrotransposons. Plant Physiol 2018;176:1410–22. https://doi.org/10.1104/pp.17.01310.
[48] Benson G. Tandem repeats finder: a program to analyze DNA sequences. Nucleic Acids Res 1999;27:573–80. https://doi.org/10.1093/nar/27.2.573.
[49] Stanke M, Keller O Gunduz I Hayes A Waack S Morgenstern B. AUGUSTUS: ab initio prediction of alternative transcripts. Nucleic Acids Res 2006;34: 435–9.

https://doi.org/10.1093/nar/gkl200.
[50] Majoros WH, Pertea M, Salzberg SL. TigrScan and GlimmerHMM:: two open source ab initio eukaryotic gene-finders. Bioinformatics 2004;20:2878–9. https://doi.org/10.1093/bioinformatics/bth315.
[51] Keilwagen J, Wenk M, Erickson JL, Schattat MH, Grau J, Hartung F. Using intron position conservation for homology-based gene prediction. Nucleic Acids Res 2016;44:e89. https://doi.org/10.1093/nar/gkw092.
[52] TransDecoder. https://github.com/TransDecoder/TransDecoder. Accessed 5 May 2024.
[53] Haas BJ, Salzberg SL, Zhu W, Pertea M, Allen JE, Orvis J, et al. Automated eukaryotic gene structure annotation using EVidenceModeler and the program to assemble spliced alignments. Genome Biol 2008;9:R7. https://doi.org/10.1186/gb-2008-9-1-r7.
[54] Buchfink B, Xie C, Huson DH. Fast and sensitive protein alignment using DIAMOND. Nat Methods 2015;12:59–60. https://doi.org/10.1038/nmeth.3176.
[55] Jones P, Binns D, Chang H-Y, Fraser M, Li W, McAnulla C, et al. InterProScan 5: genome-scale protein function classification. Bioinformatics 2014;30:1236–40. https://doi.org/10.1093/bioinformatics/btu031.
[56] Huerta-Cepas J, Forslund K, Coelho LP, Szklarczyk D, Jensen LJ, von Mering C, et al. Fast Genome-Wide Functional Annotation through Orthology Assignment by eggNOG-Mapper. Mol Biol Evol 2017;34:2115–22. https://doi.org/10.1093/molbev/msx148.
[57] Chen C, Wu Y, Li J, Wang X, Zeng Z, Xu J, et al. TBtools-II: A “one for all, all for one”bioinformatics platform for biological big-data mining. Mol Plant 2023;16:1733–42. https://doi.org/10.1016/j.molp.2023.09.010.
[58] Emms DM, Kelly S. OrthoFinder: solving fundamental biases in whole genome comparisons dramatically improves orthogroup inference accuracy. Genome Biol 2015;16:157. https://doi.org/10.1186/s13059-015-0721-2.
[59] Laetsch DR, Blaxter ML. KinFin: Software for Taxon-Aware Analysis of Clustered Protein Sequences. G3-Genes Genomes Genet 2017;7:3349–57. https://doi.org/10.1534/g3.117.300233.
[60] Capella-Gutierrez S, Silla-Martinez JM, Gabaldon T. trimAl: atool for automated alignment trimming in large-scale phylogenetic analyses. Bioinformatics 2009;25:1972–3. https://doi.org/10.1093/bioinformatics/btp348.
[61] Stamatakis A. RAxML version 8: a tool for phylogenetic analysis and post-analysis of large phylogenies. Bioinformatics 2014;30:1312–3. https://doi.org/10.1093/bioinformatics/btu033.
[62] Yang Z. PAML 4: phylogenetic analysis by maximum likelihood. Mol Biol Evol 2007;24:1586–91. https://doi.org/10.1093/molbev/msm088.

[63] Kumar S, Suleski M, Craig JM, Kasprowicz AE, Sanderford M, Li M, et al. TimeTree 5: An Expanded Resource for Species Divergence Times. Mol Biol Evol 2022;39:msac174. https://doi.org/10.1093/molbev/msac174.

[64] Krosch MN, Schutze MK, Armstrong KF, Graham GC, Yeates DK, Clarke AR. A molecular phylogeny for the Tribe Dacini (Diptera: Tephritidae): Systematic and biogeographic implications. Mol Phylogenet Evol 2012;64:513–23. https://doi.org/10.1016/j.ympev.2012.05.006.

[65] Yaakop S, Ibrahim NJ, Shariff S, Zain BMM. Molecular clock analysis on five *Bactrocera* species flies (Diptera: Tephritidae) based on combination of COI and NADH sequences. Orient Insects 2015;49:150–64. https://doi.org/10.1080/00305316.2015.1081421.

[66] Zhao Z, Su T, Chesters D, Wang S, Ho SYW, Zhu C, et al. The Mitochondrial Genome of *Elodia flavipalpis* Aldrich (Diptera: Tachinidae) and the Evolutionary Timescale of Tachinid Flies. PLoS One 2013;8:e61814. https://doi.org/10.1371/journal.pone.0061814.

[67] Russo CAM, Mello B, Frazao A, Voloch CM. Phylogenetic analysis and a time tree for a large drosophilid data set (Diptera: Drosophilidae). Zool J Linn Soc 2013;169:765–75. https://doi.org/10.1111/zoj12062.

[68] Gaunt MW, Miles MA. An insect molecular clock dates the origin of the insects and accords with palaeontological and biogeographic landmarks. Mol Biol Evol 2002;19:748–61. https://doi.org/10.1093/oxfordjournals.molbev.a004133.

[69] Nardi F, Carapelli A, Boore JL, Roderick GK, Dallai R, Frati F. Domestication of olive fly through a multi-regional host shift to cultivated olives: Comparative dating using complete mitochondrial genomes. Mol Phylogenet Evol 2010;57:678–86. https://doi.org/10.1016/j.ympev.2010.08.008.

[70] Xie J, Chen Y, Cai G, Cai R, Hu Z, Wang H. Tree Visualization By One Table (tvBOT): a web application for visualizing, modifying and annotating phylogenetic trees. Nucleic Acids Res 2023;51:W587–92. https://doi.org/10.1093/nar/gkad359.

[71] De Bie T, Cristianini N, Demuth JP, et al. CAFE: a computational tool for the study of gene family evolution. Bioinformatics 2006;22:1269–71. https://doi.org/10.1093/bioinformatics/btl097.

[72] Finn RD, Bateman A, Clements J, Coggill P, Eberhardt RY, Eddy SR, et al. Pfam: the protein families database. Nucleic Acids Res 2014;42:222–30. https://doi.org/10.1093/nar/gkt1223.

[73] FlyBase. http://flybase.org. Accessed 10 October 2023.

[74] McGinnis S, Madden TL. BLAST: at the core of a powerful and diverse set of sequence analysis tools. Nucleic Acids Res 2004;32:20–5. https://doi.org/10.1093/nar/gkh435.

[75] Potter SC, Luciani A, Eddy SR, et al. HMMER web server: 2018 update. Nucleic Acids Res 2018;46:W200–4. https://doi.org/10.1093/nar/gky448.
[76] Vizueta J, Sanchez-Gracia A, Rozas J. bitacora: A comprehensive tool for the identification and annotation of gene families in genome assemblies. Mol Ecol Resour 2020;20:1445–52. https://doi.org/10.1111/1755-0998.13202.
[77] Edgar RC. MUSCLE: multiple sequence alignment with high accuracy and high throughput. Nucleic Acids Res 2004;32:1792–7. https://doi.org/10.1093/nar/gkh340.
[78] Minh BQ, Schmidt HA, Chernomor O, et al. IQ-TREE 2: new models and efficient methods for phylogenetic inference in the genomic era. Mol Biol Evol 2020;37:1530–4. https://doi.org/10.1093/molbev/msaa015.
[79] Kim D, Paggi JM, Park C, Bennett C, Salzberg SL. Graph-based genome alignment and genotyping with HISAT2 and HISAT-genotype. Nat Biotechnol 2019;37:907–15. https://doi.org/10.1038/s41587-019-0201-4.
[80] Liao Y, Smyth GK, Shi W. The R package Rsubread is easier, faster, cheaper and better for alignment and quantification of RNA sequencing reads. Nucleic Acids Res 2019;47:e47. https://doi.org/10.1093/nar/gkz114.
[81] Mu H, Chen J, Huang W, Huang G, Deng M, Hong S, et al. OmicShare tools: A zero-code interactive online platform for biological data analysis and visualization. iMeta 2024;3. https://doi.org/10.1002/imt2.228.
[82] Romeo JT. New SPME guidelines. J Chem Ecol 2009;35:1383. https://doi.org/10.1007/s10886-009-9733-2.
[83] Bradford M. Rapid and Sensitive Method for Quantitation of Microgram Quantities of Protein Utilizing Principle of Protein-Dye Binding. Anal Biochem 1976;72:248–54. https://doi.org/10.1016/0003-2697(76)90527-3.
[84] Jumper J, Evans R, Pritzel A, Green T, Figurnov M, Ronneberger O, et al. Highly accurate protein structure prediction with AlphaFold. Nature 2021;596:583–9. https://doi.org/10.1038/s41586-021-03819-2.
[85] PubChem. https://pubchem.ncbi.nlm.nih.gov. Accessed 10 September 2024.
[86] Graef J, Ehrt C, Rarey M. Binding Site Detection Remastered: Enabling Fast, Robust, and Reliable Binding Site Detection and Descriptor Calculation with DoGSite3. J Chem Inf Model 2023;63:1–10. https://doi.org/10.1021/acs.jcim.3c00336.
[87] Volkamer A, Griewel A, Grombacher T, Rarey M. Analyzing the Topology of Active Sites: On the Prediction of Pockets and Subpockets. J Chem Inf Model 2010;50:2041–52. https://doi.org/10.1021/ci100241y.

[88] Volkamer A, Kuhn D, Grombacher T, Rippmann F, Rarey M. Combining Global and Local Measures for Structure-Based Druggability Predictions. J Chem Inf Model 2012;52:360–72. https://doi.org/10.1021/ci200454v.
[89] PyMOL. http://www.pymol.org/pymol. Accessed 20 October 2024.
[90] Trott O, Olson AJ. Software News and Update AutoDock Vina: Improving the Speed and Accuracy of Docking with a New Scoring Function, Efficient Optimization, and Multithreading. J Comput Chem 2010;31:455–61. https://doi.org/10.1002/jcc.21334.
[91] Van der Spoel D, Lindahl E, Hess B, Groenhof G, Mark AE, Berendsen HJC. GROMACS: Fast, flexible, and free. J Comput Chem 2005;26:1701–18. https://doi.org/10.1002/jcc.20291.
[92] Abraham MJ, Murtola T, Schulz R, Páll S, Smith JC, Hess B, et al. GROMACS: High performance molecular simulations through multi-level parallelism from laptops to supercomputers. SoftwareX 2015;1–2:19–25. https://doi.org/10.1016/j.softx.2015.06.001.
[93] Li S, Zhu S, Jia Q, Yuan D, Ren C, Li K, et al. The genomic and functional landscapes of developmental plasticity in the American cockroach. Nat Commun 2018;9:1008. https://doi.org/10.1038/s41467-018-03281-1.
[94] Francis F, Vanhaelen N, Haubruge E. Glutathione S-transferases in the adaptation to plant secondary metabolites in the *Myzus persicae* aphid. Arch Insect Biochem Physiol 2005;58:166–74. https://doi.org/10.1002/arch.20049.
[95] Jin R, Mao K, Liao X, Xu P, Li Z, Ali E, et al. Overexpression of *CYP6ER1* associated with clothianidin resistance in *Nilaparvata lugens* (Stal). Pest Biochem Physiol 2019;154:39–45. https://doi.org/10.1016/j.pestbp.2018.12.008.
[96] Feder ME, Hofmann GE. Heat-shock proteins, molecular chaperones, and the stress response: Evolutionary and ecological physiology. Annu Rev Physiol 1999;61:243–82. https://doi.org/10.1146/annurev.physiol.61.1.243.
[97] García-Reina A, Rodríguez-García MJ, Ramis G, et al. Real-time cell analysis and heat shock protein gene expression in the TcA *Tribolium castaneum* cell line in response to environmental stress conditions: RTCA and hsps expression in the TcA cell line. Insect Sci 2017;24:358–70. https://doi.org/10.1111/1744-7917.12306.
[98] Lu K, Chen X, Liu W, et al. Characterization of heat shock protein 70 transcript from *Nilaparvata lugens* (Stål): its response to temperature and insecticide stresses. Pestic Biochem Physiol 2017;142:102–10. https://doi.org/10.1016/j.pestbp.2017.01.011.
[99] Eyun S, Soh HY, Posavi M, Munro JB, Hughes DST, Murali SC, et al. Evolutionary History of Chemosensory-Related Gene Families across the Arthropoda. Molecular Biology and Evolution 2017;34:1838–62. https://doi.org/10.1093/molbev/msx147.

[100] Robertson HM. Molecular Evolution of the Major Arthropod Chemoreceptor Gene Families. Annual Review of Entomology 2019;64:227–42. https://doi.org/10.1146/annurev-ento-020117-043322.

[101] Vogt RG, Miller NE, Litvack R, Fandino RA, Sparks J, Staples J, et al. The insect SNMP gene family. Insect Biochem Mol Biol 2009;39:448–56. https://doi.org/10.1016/j.ibmb.2009.03.007.

[102] Xu PX, Atkinson R, Jones DNM, Smith DP. *Drosophila* OBP LUSH is required for activity of pheromone-sensitive neurons. Neuron 2005;45:193–200. https://doi.org/10.1016/j.neuron.2004.12.031.

[103] Sessegolo C, Burlet N, Haudry A. Strong phylogenetic inertia on genome size and transposable element content among 26 species of flies. Biol Lett 2016;12:20160407. https://doi.org/10.1098/rsbl.2016.0407.

[104] Zhao L, Yuan H, Liu X, Chang H, Jing X, Nie Y, et al. Evolutionary dynamics of repetitive elements and their relationship with genome size in Acrididae. Genomics 2025;117:110971. https://doi.org/10.1016/j.ygeno.2024.110971.

[105] Cabral-de-Mello DC, Palacios-Gimenez OM. Repetitive DNAs: the “invisible” regulators of insect adaptation and speciation. Curr Opin Insect Sci 2025;67:101295. https://doi.org/10.1016/j.cois.2024.101295.

[106] Jiang F, Liang L, Wang J, Zhu S. Chromosome-level genome assembly of *Bactrocera dorsalis* reveals its adaptation and invasion mechanisms. Commun Biol 2022;5:25. https://doi.org/10.1038/s42003-021-02966-6.

[107] Guo T, Feng S, Zhang Y, Li W, Qin Y, Li Z. Chromosome-level genome assembly of *Bactrocera correcta* provides insights into its adaptation and invasion mechanisms. Genomics 2023;115:110736. https://doi.org/10.1016/j.ygeno.2023.110736.

[108] He X, Tzotzos G, Woodcock C, Pickett JA, Hooper T, Field LM, et al. Binding of the General Odorant Binding Protein of Bombyx mori BmorGOBP2 to the Moth Sex Pheromone Components. J Chem Ecol 2010;36:1293–305. https://doi.org/10.1007/s10886-010-9870-7.

[109] Liu Y, Gu S, Zhang Y, Guo Y, Wang G. Candidate Olfaction Genes Identified within the Helicoverpa armigera Antennal Transcriptome. PLoS One 2012;7:e48260. https://doi.org/10.1371/journal.pone.0048260.

[110] Wang Y, Fang G, Xu P, Gao B, Liu X, Qi X, et al. Behavioral and genomic divergence between a generalist and a specialist fly. Cell Reports 2022;41:111654. https://doi.org/10.1016/j.celrep.2022.111654.

[111] Fonseca PM, Robe LJ, Carvalho TL, Loreto ELS. Characterization of the chemoreceptor repertoire of a highly specialized fly with comparisons to other Drosophila species. Genet Mol

Biol 2024;47:e20220383. https://doi.org/10.1590/1678-4685-GMB-2022-0383.

[112] Nauen R, Bass C, Feyereisen R, et al. The role of cytochrome P450s in insect toxicology and resistance. Annu Rev Entomol 2022;67:105–24. https://doi.org/10.1146/annurev-ento-070621-061328.

[113] Duan S, Mao L, Sun S, Chen R, Abdelkhalek ST, Wang M. Key site residues of *Cnaphalocrocis medinalis* odorant-binding protein 13 CmedOBP13 involved in interacting with rice plant volatiles. Int J Biol Macromol 2025;290:139007. https://doi.org/10.1016/j.ijbiomac.2024.139007.

[114] Yang Y, Tan S, Wang Q, Wang F, Zhang Y. Key amino acids in odorant-binding protein OBP7 enable *Bradysia odoriphaga* to recognize host plant volatiles. Int J Biol Macromol 2025;284:138179. https://doi.org/10.1016/j.ijbiomac.2024.138179.

[115] Chen X, Lei Y, Liang C, Lei Q, Wang J, Jiang H. Odorant Binding Protein Expressed in Legs Enhances Malathion Tolerance in *Bactrocera dorsalis* (Hendel). J Agric Food Chem 2024;72:4376–83. https://doi.org/10.1021/acs.jafc.3c08458.

[116] Vosshall LB, Amrein H, Morozov PS, Rzhetsky A, Axel R. A spatial map of olfactory receptor expression in the *Drosophila* antenna. Cell 1999;96:725–36. https://doi.org/10.1016/S0092-8674(00)80582-6.

[117] Miyazaki H, Otake J, Mitsuno H, Ozaki K, Kanzaki R, Chieng AC-T, et al. Functional characterization of olfactory receptors in the Oriental fruit fly *Bactrocera dorsalis* that respond to plant volatiles. Insect Biochem Mol Biol 2018;101:32–46. https://doi.org/10.1016/j.ibmb.2018.07.002.

[118] Xu L, Jiang H-B, Yu J-L, Lei Q, Pan D, Chen Y, et al. An Odorant Receptor Expressed in Both Antennae and Ovipositors Regulates Benzothiazole-Induced Oviposition Behavior in *Bactrocera dorsalis*. J Agric Food Chem 2024;72:6954–63. https://doi.org/10.1021/acs.jafc.3c09557.

[119] Wang C, Cao S, Shi C, Guo M, Sun D, Liu Z, et al. The novel function of an orphan pheromone receptor reveals the sensory specializations of two potential distinct types of sex pheromones in noctuid moth. Cell Mol Life Sci 2024;81:259. https://doi.org/10.1007/s00018-024-05303-2.

[120] Lin C-C, Prokop-Prigge KA, Preti G, Potter CJ. Food odors trigger *Drosophila* males to deposit a pheromone that guides aggregation and female oviposition decisions. Elife 2015;4. https://doi.org/10.7554/eLife.08688.

[121] Miyazaki H, Otake J, Mitsuno H, Ozaki K, Kanzaki R, Chieng AC-T, et al. Functional characterization of olfactory receptors in the Oriental fruit fly *Bactrocera dorsalis* that respond to plant volatiles. Insect Biochem Mol Biol 2018;101:32–46.

https://doi.org/10.1016/j.ibmb.2018.07.002.

[122] Ono H. Functional characterization of an olfactory receptor in the Oriental fruit fly, *Bactrocera dorsalis*, that responds to eugenol and isoeugenol. Comp Biochem Physiol B-Biochem Mol Biol 2022;258:110696. https://doi.org/10.1016/j.cbpb.2021.110696.

[123] Fleischer J, Pregitzer P, Breer H, Krieger J. Access to the odor world: olfactory receptors and their role for signal transduction in insects. Cell Mol Life Sci 2018;75:485–508. https://doi.org/10.1007/s00018-017-2627-5.

[124] Xue J, Zhou X, Zhang C, Yu L, Fan H, Wang Z, et al. Genomes of the rice pest brown planthopper and its endosymbionts reveal complex complementary contributions for host adaptation. Genome Biol 2014;15:521. https://doi.org/10.1186/s13059-014-0521-0.

[125] Steffan-Dewenter I, Tscharntke T. Butterfly community structure in fragmented habitats. Ecol Lett 2000;3:449–56. https://doi.org/10.1111/j.1461-0248.2000.00175.x.

[126] Hafsi A, Facon B, Ravigné V, Chiroleu F, Quilici S, Chermiti B, et al. Host plant range of a fruit fly community (Diptera: Tephritidae): does fruit composition influence larval performance? BMC Ecology 2016;16:40. https://doi.org/10.1186/s12898-016-0094-8.

[127] Facon B, Hafsi A, Charlery de la Masselière M, Robin S, Massol F, Dubart M, et al. Joint species distributions reveal the combined effects of host plants, abiotic factors and species competition as drivers of species abundances in fruit flies. Ecology Letters 2021;24:1905–16. https://doi.org/10.1111/ele.13825.

[128] Charlery de la Masselière M, Facon B, Hafsi A, Duyck P-F. Diet breadth modulates preference - performance relationships in a phytophagous insect community. Sci Rep 2017;7:16934. https://doi.org/10.1038/s41598-017-17231-2.

[129] Guo T;Li W;Zhang Y;Yang W;Li Z;Qin Y.(2025):Supporting data for "A high-quality chromosome-level genome assembly of the oligophagous fruit fly Bactrocera tsuneonis (Diptera: Tephritidae) and insights into its host specificity".GigaScience database.https://doi.org/10.5524/102768

Click here to access/download
Supplementary Material
Supplementary information.docx

Revised version for "A high-quality chromosome-level genome assembly of the oligophagous fruit fly *Bactrocera tsuneonis* (Diptera: Tephritidae) and insights into its host specificity"

Dear editor,

We are honored to have the opportunity to resubmit our manuscript entitled "*A high-quality chromosome-level genome assembly of the oligophagous fruit fly Bactrocera tsuneonis (Diptera: Tephritidae) and insights into its host specificity*". We would like to express our sincere gratitude to you and the reviewers for your valuable time and insightful comments, which have significantly contributed to the improvement of our manuscript. We have carefully addressed all the comments and revised the manuscript accordingly.

We hereby confirm that the manuscript has not been published, accepted for publication, or is under consideration for publication elsewhere, either in whole or in part. This work is original, and all necessary acknowledgements have been made. The submission has been approved by all authors and affiliated institutions, and all authors have read and agreed to the final version of the manuscript. Please feel free to contact us if any further information is needed.

Yours sincerely,

Corresponding Author: Yujia Qin qinyujia@cau.edu.cn; Zhihong Li lizh@cau.edu.cn

First Author: Tengda Guo guotd7911@163.com

Reviewer #3

Comment 1: This assembly is really PacBio + HiC, the Illumina data was not used for assembly. Illumina data was used in kmer based analysis, but the HiFi data alone could also have been used for this same analysis.

Authors' reply: We have revised the text to clarify data usage. Illumina short reads were used only for genome survey and quality evaluation, while the final chromosome-level assembly was generated solely from PacBio HiFi and Hi-C data. Line 102-104: "In this study, we assembled a high-quality chromosome-level genome of *B. tsuneonis* using a combination of PacBio high-fidelity (HiFi) sequencing, and high-resolution chromosome conformation capture (Hi-C) technologies". Line 170-171: "The Illumina

short reads were used solely for this purpose and were not included in the assembly process". Line 379-380: "A total of 24.91 Gb of Illumina short reads, 37.82 Gb of PacBio HiFi long reads, and 58.88 Gb of Hi-C data were generated".

Comment 2: in the methods, full length transcriptome sequencing was mentioned (essentially IsoSeq), but it was unclear how it was analyzed and how it was used for overall genome annotation, manual annotation of genes (particularly can be useful for chemosensory genes) or tissue specific analyses. How was this data used?

Authors' reply: We have revised the Methods section to clarify the use of Iso-Seq data in genome annotation. Line 200-203: "Simultaneously, full-length transcriptomic (Iso-Seq) data were incorporated into the annotation workflow to support transcriptome-based gene prediction. The polished Iso-Seq isoforms were aligned to the assembled genome and used to enhance gene model accuracy". Line 433-435: "Protein-coding genes in the *B. tsuneonis* genome were predicted using a combination of three approaches: ab initio prediction, homology-based prediction, and full-length transcriptome (Iso-Seq)-based prediction".

Comment 3: I was able to follow, but really a better description of how plant chemistry is being used in combination of protein structure prediction, modeling, and protein expression needs to be made. Really walk people through how the structure of the insect proteins is being compared to the compounds present in host fruit, etc etc.

Authors' reply: We have expanded the manuscript to better describe the integration of plant chemistry with protein modeling and molecular simulations. Line 92-101: "Molecular dynamics (MD) simulation techniques are widely used to predict the binding affinity, binding modes, and interaction mechanisms between proteins and their ligands [28]. In olfactory research, molecular docking is commonly applied to identify binding sites and interaction modes between ORs and their specific volatile ligands, whereas MD simulations further reveal the structural stability and conformational dynamics of receptor-ligand complexes [29]. This method provides advantages in analyzing the three-dimensional structures, charge distributions, and interaction mechanisms of proteins (including ORs), offering new insights into the molecular basis of odorant recognition [27,29]. With the rapid advancement of computational technology, MD simulations have become increasingly efficient, serving as a powerful tool for elucidating protein functions and exploring OR-ligand recognition mechanisms

[30]". Line 276-277: "To identify the host-derived volatile compounds of host fruit (*Maoping Tangerine*) for subsequent GC-MS analysis, volatile chemicals were collected using solid-phase microextraction (SPME)". Line 358-360: "To further evaluate the binding stability and conformational dynamics of odorant receptors (BtsuOr7a-6 and BtsuOr7a-4) complexed with their respective ligands (trans-nerolidol and piperitone), MD simulations were performed". Line 563-566: "Based on the SPME-GC – MS analysis and fluorescence binding assays, trans-nerolidol and piperitone were identified as the two major host-derived volatiles that showed strong binding affinity to BtsuOBP83a. To further elucidate the molecular recognition mechanisms underlying these interactions, molecular docking was performed using the predicted OBP structure".

Comment 4: Take a closer look at chromosome structure in Tephritid fruit flies. You report changes in chromosome number, but a lot of that is related to chromosome naming. Please attempt to say "autosomes", X and Y or if unknown be clear that the autosomes can't be separated from sex chromosome. One application of Illumina data can be to characterize the chromosome as a sex chromosome based on baised coverage in females vs males (e.g. AD ratio). This could be used to better assign your chromosomes as autosomes (there are 5) and X, rather than reporting as having 6 chromosomes (which is misleading). You also can review classical cytogenetic experiments as evidence.

Authors' reply: We have revised the Results section (Table 1) to clarify chromosome naming and to distinguish between autosomes and sex chromosomes. The revised manuscript notes that *B. tsuneonis* likely possesses five autosomes and one X chromosome, with Chr2 showing collinearity to the *D. melanogaster* X chromosome. Line 412-413: "Together, these results indicate that *B. tsuneonis* likely possesses a karyotype of five autosomes and one X chromosome (5 + X)". While Illumina read coverage–based sex chromosome assignment (AD ratio) was not performed in this study, we acknowledge that such analyses, along with classical cytogenetic evidence, will be valuable for future work.

In addition, we have carefully reviewed all comments and annotations provided in the marked-up PDF from Reviewer #3. Detailed responses have been inserted directly after

each comment within the annotated PDF, ensuring that every point has been addressed clearly and specifically. We sincerely appreciate these constructive suggestions, which have further improved the accuracy and clarity of the revised manuscript.

Manuscript
Response to Decision Letter (transfer desk only) Click here to access/download;Manuscript;Manuscript.docx

A high-quality chromosome-level genome assembly of the oligophagous fruit fly *Bactrocera tsuneonis* (Diptera: Tephritidae) and insights into its host specificity

Tengda Guo[1,2], Weisong Li[1], Yuan Zhang[1,3], Wenzhao Yang[1], Zhihong Li[1,*], Yujia Qin[1,*]

[1]State Key Laboratory of Agricultural and Forestry Biosecurity, MARA Key Laboratory of Surveillance and Management for Plant Quarantine Pests, College of Plant Protection, China Agricultural University, Beijing 100193, China

[2]Shenzhen Branch, Guangdong Laboratory of Lingnan Modern Agriculture, Key Laboratory of Synthetic Biology, Ministry of Agriculture and Rural Affairs, Agricultural Genomics Institute at Shenzhen Chinese Academy of Agricultural Sciences, Shenzhen 518120, China

[3]School of Ecology and State Key Laboratory of Biocontrol, Shenzhen Campus of Sun Yat-Sen University, Shenzhen 518107, China.

*Correspondence: Yujia Qin, E-mail: qinyujia@cau.edu.cn; Zhihong Li, E-mail: lizh@cau.edu.cn

Abstract

Background: *Bactrocera tsuneonis* is a major pest of citrus, causing significant economic losses in fruit production. It exhibits a highly specialized host preference, primarily infesting citrus fruits. However, the genetic basis underlying its olfactory adaptation and host specificity remains largely unexplored. To elucidate the molecular mechanisms governing host selection in *B. tsuneonis*, we assembled a high-quality chromosome-level genome and performed comparative genomic, transcriptomic, and functional analyses of its chemosensory system.

Results: The genome of *B. tsuneonis* was assembled to a total size of 339 Mb, with a contig N50 of 11.21 Mb and a scaffold N50 of 59.93 Mb. Comparative genomic analysis revealed significant contractions in chemosensory-related gene families, particularly in odorant-binding proteins (OBPs) and odorant receptors (ORs), maybe suggesting an adaptation to a narrow host range. Transcriptome analysis demonstrated that *BtsuOBP83a* and *BtsuOBP83b* were highly expressed in the antennae, while most ORs were predominantly expressed in the antennae. Functional assays confirmed that *BtsuOBP83a* selectively binds to two citrus volatiles, *trans*-nerolidol and piperitone, with strong affinity. Molecular docking and molecular dynamics simulations further revealed that *BtsuOr7a-6* and *BtsuOr7a-4* specifically interact with these volatiles, suggesting their role in host odor recognition.

Conclusions: Our high-quality genome of *B. tsuneonis* provides a valuable resource for genomic research and offers valuable insights into the genetic basis of its olfactory adaptation and host specificity. The findings highlight key molecular mechanisms underlying host selection and provide potential targets for behavior-based pest management strategies.

Keywords: *Bactrocera tsuneonis*, genome, comparative genomics, olfactory proteins, oligophagous

Introduction

Bactrocera tsuneonis (Miyake), Japanese orange fly, belongs to Diptera, Tephritidae, and is one of the most serious pests affecting citrus crops [1]. Its distribution is mainly restricted to China and Japan, but it has the potential to spread beyond Asia [2]. *B. tsuneonis* uses its ovipositor to penetrate unripe citrus fruits for oviposition, and the larvae feed on the internal tissues of the host fruit, causing significant damage to fruit quality and yield [3]. ~~Statistical data indicate that,~~ on average, 10% to 20% of citrus yields may be lost due to infestation, and if not effectively controlled, losses could exceed 50% [4]. As citrus is one of the most widely cultivated and produced fruits globally [5], due to the damaging characteristics of *B. tsuneonis* and its recognition as a significant international quarantine pest, this species warrants global attention for strengthened prevention and control efforts. Despite its economic and environmental impact, comprehensive research on the genetic factors contributing to its adaptability and invasiveness remains lacking.

The advancement of genomic tools, particularly the availability of high-quality assembled genomes, has significantly facilitated the investigation of the genetic factors driving the global distribution and diversity of various organisms [6,7]. *Bactrocera* species are highly invasive and adaptable, with females ovipositing in host plants and larvae feeding on the fruit, leading to substantial agricultural losses [8,9]. While significant progress has been achieved in genomics research for other insects, genome annotations have been published for only 7 species within the *Bactrocera* genus (*B. correcta*, *B. dorsalis*, *B. tryoni*, *B. latifrons*, *B. oleae*, *B. neohumeralis* and *B. minax*) in NCBI (https://www.ncbi.nlm.nih.gov/datasets/genome/). Additionally, only *B. correcta*, *B. dorsalis*, *B. tryoni*, *B. oleae* and *B. neohumeralis* have reported genome assemblies at the chromosome level. Given the economic and ecological importance of *B. tsuneonis*, investigating its genetic foundation is essential for advancing research on its biology, ecology, and evolutionary adaptations, as well as for developing more effective pest management strategies.

The host range of *B. tsuneonis* is limited to Citrus species, exhibiting oligophagous, which differs from many polyphagous fruit flies, such as *B. dorsalis*, which can infest fruits from multiple plant

families [10]. Understanding how *B. tsuneonis* selects its host to complete oviposition is essential for elucidating its ecological adaptability. In insects, the chemosensory system plays a pivotal role in host localization and recognition, with odorant-binding proteins (OBPs) and odorant receptors (ORs) being particularly crucial for detecting host volatiles [11,12]. OBPs are small, globular, water-soluble acidic proteins that are widely distributed in the lymphatic fluid. Their interaction with odorant molecules constitutes the initial biochemical step in external odor recognition [13]. These proteins typically consist of 120–160 amino acids, with a molecular weight of approximately 15–20 kDa. At the N-terminus, they contain a signal peptide of about 20 amino acids, which is cleaved during secretion to yield the mature protein. A distinctive characteristic of OBPs is the presence of six conserved cysteine residues that form three disulfide bonds (C1–C3, C2–C5, C4–C6), ensuring structural stability [14,15,16]. Functional characterization of OBPs typically involves expressing recombinant proteins in a prokaryotic system, followed by purification and ligand-binding analysis. Fluorescence-based competitive binding assays are commonly employed to identify specific odorant ligands for OBPs, providing insights into their role in olfactory perception [17,18,19].

When odorant molecules reach the membrane of olfactory neurons, they are released and activate ORs, converting chemical signals into electrical impulses that are subsequently processed and transmitted to the central nervous system [20]. ORs, located on the dendritic membranes of olfactory neurons, are key components of the peripheral olfactory system in insects [21]. The specific odorant detected by an OR is referred to as its ligand. Insect ORs are broadly categorized into two types: the atypical odorant receptor co-receptor (ORco) and conventional odorant receptors. ORco is a highly conserved protein across insect species and does not independently recognize odorants [22]. However, conventional ORs require ORco to function properly. Structurally, insect ORs are membrane proteins characterized by seven α-helical transmembrane domains. While they share some structural similarities with mammalian G protein-coupled receptors (GPCRs), insect ORs exhibit a distinct orientation, with the C-terminus located extracellularly and the N-terminus intracellularly [23,24]. Recent cryo-electron microscopy studies have revealed that insect ORs assemble into tetrameric complexes with ORco to facilitate signal transduction. This structural organization is essential for odor recognition and plays a critical role in insect olfactory perception [25,26,27].

In this study, we assembled a high-quality chromosome-level genome of *B. tsuneonis* using a combination of Illumina short-read sequencing, PacBio high-fidelity (HiFi) sequencing, and high-resolution chromosome conformation capture (Hi-C) technologies. Through comparative genomic and gene family analyses, we explored the evolutionary dynamics of chemosensory gene families, particularly odorant-binding proteins (OBPs) and odorant receptors (ORs), in *B. tsuneonis* relative to polyphagous fruit flies. Furthermore, we examined the interactions between olfactory-related proteins

and key host volatiles using gas chromatography-mass spectrometry (GC-MS), fluorescence competitive binding assay, structural modeling, and molecular dynamics simulations to elucidate the molecular mechanisms underlying volatile recognition in *B. tsuneonis*. The findings from this study provide a foundational genetic resource for future research, contributing to a deeper understanding of host recognition mechanisms in oligophagous pests and offering theoretical support for the development of behavior-based pest management strategies targeting chemosensory pathways.

Methods

Sample preparation

The *B. tsuneonis* samples used in this study were collected from a natural wild population in Pingshan, Yibin, Sichuan Province. Larval samples were collected in September 2022 from infested fruits in orchards, while adult samples were obtained in January 2020 by excavating pupae of *B. tsuneonis* from infested orchards and bringing them back to the laboratory, where they were kept in a constant temperature and humidity artificial intelligence climate box until emergence. The parameters of the climate box are set at a constant temperature of 25°C, a humidity level of 70%, and a light period of 10 h of daylight followed by 14 h of darkness. All samples underwent molecular identification prior to experiments, including DNA extraction, and RNA extraction, to confirm that they were *B. tsuneonis* [28].

Genomic DNA and RNA sequencing

For Illumina sequencing, genomic DNA was extracted from a single male adult (with the abdomen removed) using the Wizard SV Genomic DNA Purification System Kit from Promega, and the quality and concentration of the DNA were measured using a microvolume UV spectrophotometer. ~~A second-generation sequencing library~~ with an average insert size of 350 bp was then constructed. The sequencing was performed by Berry Genomics (Beijing, China) on the Illumina Novaseq platform.

For long-read sequencing, genomic DNA was extracted from a single male adult (with the abdomen removed). A HiFi SMRTbell library with an insert size of 15 Kb was constructed, and the sequencing of the long-read library was carried out by Berry Genomics (Beijing, China) on the PacBio Sequel II platform, generating circular consensus sequence (CCS) reads.

For Hi-C sequencing, genomic DNA was extracted from larvae after three days of starvation treatment. The samples were fixed with formaldehyde, and DNA was digested using the restriction enzyme DpnII, ~~breaking it~~ into approximately 400 bp fragments. DNA fragments containing interaction relationships were captured to construct the library. Sequencing was performed by Berry Genomics (Beijing, China) on the Illumina Novaseq/MGI-2000 platform.

For full-length transcriptome sequencing, total RNA was extracted from the abdomen-removed fruit

fly using the SV Total RNA Isolation System Kit from Promega. The concentration was precisely quantified using the Agilent 2100 RNA 6000 Nano kit, and samples with an RIN greater than 7.5 were selected to construct a 1-10 Kb SMRTbell library. Sequencing was conducted by Berry Genomics (Beijing, China) on the PacBio Sequel II platform.

For transcriptome sequencing, different body parts, including 50 antennae,5 heads (without antennae),10 legs, and 5 ovipositors (female only), were dissected separately from field-collected female and male adults. Three biological replicates were prepared for each tissue type. Total RNA was extracted using standard protocols. RNA sequencing libraries were constructed using the Illumina TruSeq RNA Library Preparation Kit and sequenced on an Illumina platform by Personalbio (Shanghai, China), generating 150 bp paired-end reads.

Genome assembly and evaluation

To assess the genome characteristics of *B. tsuneonis*, including genome size, heterozygosity, repeat content, and GC composition, a genome survey was conducted using Illumina sequencing data. K-mer analysis was performed with Jellyfish version 2.2.1 [29] to generate the k-mer frequency distribution, followed by genome statistical evaluation using GenomeScope version 2.0 [30].

In genome assembly, Hifiasm version 0.19.3 is used for high-quality assembly of PacBio CCS data [31]. By loading all CCS reads into memory, Hifiasm performs an all-vs-all comparison and error correction, relying only on data from the same haplotype to avoid overcorrection. For highly heterozygous genomes, the initial assembly may assemble all heterozygous fragments, resulting in a genome size larger than expected. To address this, Purge_dups version 1.2.3 is used to remove redundant sequences [32].

To anchor the genome assembly to chromosome-scale linkage groups, Hi-C analysis was conducted. Following quality filtering of the Hi-C reads, the cleaned Hi-C reads were mapped to the draft genome using BWA version 0.7.17 [33]. Paired-end reads uniquely aligned to the draft genome were selected based on restriction sites identified from the Hi-C data. Using 3D-DNA version 180114 and Juicer version 1.6, reads were then clustered to build scaffolds [34,35]. Scaffold arrangement was validated by assessing interaction strengths between read pairs. The scaffold order underwent a detailed review and manual adjustments were made, with orientations assigned to each cluster group.

Genome annotation

To identify and annotate repetitive elements in the *B. tsuneonis* genome, we employed RepeatMasker version 4.1.5 using Dfam release 3.8 and RepBase edition 20181026 as reference databases [36,37,38]. A de novo repeat library was constructed with RepeatModeler version 2.0.5 [39]. Long terminal repeat (LTR) retrotransposons were identified using LTR Finder version 1.0726 and LTR Retriever version 2.9.028 [40,41]. Tandem repeats were annotated with Tandem Repeats Finder

version 4.09.1 [42].

A comprehensive gene annotation pipeline integrating ab initio, homology-based, and transcriptome-based predictions was applied to establish a high-confidence gene set. First, Augustus version 3.3.3 [43] and GlimmerHMM version 3.0.4 [44] were used for ab initio prediction, leveraging species-specific training models and hidden Markov models (HMMs) to identify potential coding regions. Second, homology-based prediction was conducted using GeMoMa version 1.9 [45], incorporating protein sequences from *D. melanogaster*, *B. minax*, *B. correcta*, *B. dorsalis*, *B. oleae*, *B. latifrons*, and *B. tryoni* to improve gene model reliability. Simultaneously, full-length transcriptomic data were utilized for transcriptome-based prediction, and TransDecoder version 5.1.0 (https://github.com/TransDecoder/TransDecoder) was employed to identify open reading frames (ORFs) and obtain complete protein-coding sequences (CDSs). Subsequently, EVidenceModeler version 1.1.1 [46] was used to integrate the results from the three approaches, assigning different weights based on the confidence level of each data source to generate a comprehensive gene set. Finally, PASA version 2.5.2 [46] was applied to refine gene models by correcting exon boundaries, annotating untranslated regions (UTRs), and identifying novel transcripts, resulting in a high-quality genome annotation dataset.

Functional annotation of protein sequences was performed using multiple databases and tools: (i) Diamond version 2.1.8.162 [47] for the NCBI nr database; (ii) InterProScan version 5.63-95.0 [48] for annotating Gene Ontology (GO) terms, signal peptides (SignalP), and InterPro annotations; and (iii) eggNOG-mapper version 2.1.12 [49] to annotate Clusters of Orthologous Genes (COG) categories and KEGG pathways.

Chromosomal synteny analysis

To investigate the structural characteristics of the *B. tsuneonis* genome, a chromosomal synteny analysis was conducted using two reference species: the model organism *D. melanogaster* (NCBI: GCF_000001215.4) and the closely related *B. dorsalis* (NCBI: GCA_023373825.1). These chromosome-level genomes were selected from published Tephritidae genomes for comparative analysis. Synteny analysis was performed using the One Step MCScanX tool in TBtools-II [50]. Genome sequence files and corresponding GFF annotation files were provided as input to detect and analyze homologous gene blocks. The Dual Synteny Plot for MCScanX tool was subsequently used to generate visual representations of syntenic relationships between chromosomes based on the processed configuration files.

Orthology prediction and inference of phylogenetic relationships

To infer the phylogenetic relationships of *B. tsuneonis* with other insect species, we selected 16 additional species for comparative analysis (Fig. 2). The complete protein sequences of 17 insect

species were used, with *D. melanogaster* designated as the outgroup. OrthoFinder version 2.5.4 [51] was employed to identify gene families across the selected species. Based on the OrthoFinder results, gene family clusters were categorized into four groups: single-copy genes, multiple-copy genes, species-specific (unique) genes, and unassigned genes. Functional annotation of gene families was performed using KinFin version 1.0 [52], which assigned dominant functional categories based on the most prevalent annotations among cluster members.

The phylogenetic tree was constructed based on the multiple sequence alignment of single-copy orthologous genes from each species. Multiple sequence alignments were filtered using TrimAl version 1.4.rev15 [53], and a maximum-likelihood phylogenetic tree was inferred using raxmlHPC-PTHREADS [54] based on the processed sequences.

Divergence times were estimated using the MCMCTree tool in PAML version 4.10.7 [55] based on the approximate likelihood method. Calibration points were determined from previous studies and three reference points from the TIMETREE database [56]: *Z. cucurbitae*–*Z. tau* (9.8 Mya) [57], *Zeugodacus*–*Bactrocera* (21.6–86.3 Mya) [57,58,59], Tephritidae–Drosophilidae (111.4–149 Mya) [57,60,61,62]. Visualization and analysis of phylogenetic trees were performed using tvBOT [63].

Gene family analysis

Gene family expansion and contraction among species were analyzed using CAFE version 5 [64], with OrthoFinder results and the phylogenetic tree, including divergence time estimates, as input data. The analysis accounted for phylogenetic tree topology and branch lengths when evaluating the significance of gene family size changes in each branch. Gene families with conditional P-values below 0.05 were considered to have undergone a significantly accelerated rate of expansion or contraction.

Manual annotation was performed for five detoxification-related gene families, including ATP-binding cassette (ABC) transporters, glutathione S-transferases (GSTs), cytochrome P450 monooxygenases (CYP450s), UDP-glucuronosyltransferases (UGTs), and carboxyl/cholinesterases (CCEs). Additionally, the heat shock protein (HSP) family and chemosensory-related gene families, including odorant-binding proteins (OBPs), odorant receptors (ORs), ionotropic receptors (IRs), gustatory receptors (GRs), chemosensory proteins (CSPs), and sensory neuron membrane proteins (SNMPs), were also manually annotated. HMMs for these gene families were retrieved from the Pfam database [65]. Reference protein sequences for each gene family in *D. melanogaster* were obtained from FlyBase (http://flybase.org/) and the NCBI database.

To identify gene family members, both BLAST version 2.10.0 [66] and HMMER version 3.3.2 [67] were employed, with BITACORA version 1.3 [68] used to integrate results in protein mode, applying an e-value threshold of 1e-5. Protein sequences of annotated OR and OBP genes were aligned using

MUSCLE version 3.8.1551 [69]. Phylogenetic trees were constructed with IQTREE version 2.2.3 [70] using the neighbor-joining method, with 1000 bootstrap replicates. Tree visualization and annotation were performed using tvBOT.

Transcriptomes of different tissues

Clean reads from transcriptome sequencing of different tissues were aligned to the assembled *B. tsuneonis* genome using Hisat2 version 2.2.1 [71]. Quantification analysis was conducted using Rsubread version 2.18.0 [72]. The expression levels of OBPs and ORs in various tissues were visualized as heatmaps generated using OmicShare tools [73]. To validate the reliability of transcriptome-based expression levels, qRT-PCR was performed using antennal RNA for five OBP genes (*BtsuOBP83a*, *BtsuOBP83b*, *BtsuOBP19a-1*, *BtsuOBP84a-1*, and *BtsuOBP28a-2*) and five OR (*BtsuORco*, *BtsuOR7a-4*, *BtsuOR67c-1*, *BtsuOR30a-3*, and *BtsuOR7a-6*) with the highest antenna-specific expression. Relative expression values were compared with RNA-seq data to assess correlation between the two methods. Primer sequences are provided in Supplementary Table S1.

Chemical extracts

Approximately 5 g of host fruit (*Maoping Tangerine*) was placed in a 20 mL sample vial, which was then sealed with a silicone septum-containing cap. Headspace extraction was conducted using a manual sampler equipped with a preconditioned 50/30 μm DVB/CAR/PDMS fiber. The fiber was inserted into the vial and exposed to the headspace for 40 minutes at an extraction temperature of 30°C. Following extraction, the fiber was immediately transferred to the gas chromatography (GC) injection port for desorption for 5 minutes. To minimize background interference, a blank sample (empty vial) was processed using the same protocol at the beginning of each experimental group. The 50/30 μm DVB/CAR/PDMS fiber was conditioned at 270°C for 30 minutes before its first use [74]. Sample vials were cleaned sequentially with distilled water and anhydrous ethanol, air-dried, and baked at 200°C for 2 hours to eliminate potential contamination. Each host fruit sample was analyzed in three biological replicates.

Gas chromatography coupled with mass spectrometry (GC-MS) analysis

Volatile compounds from host fruits were analyzed using a 7890B/7200 Quadrupole Time-of-Flight GC/MS system (Agilent, USA). The GC inlet temperature was set to 260°C, and analyses were performed in splitless mode. High-purity helium gas (99.999%) was used as the carrier gas at a constant flow rate of 1.0 mL/min, with a column head pressure of 0.102 kg/cm^2 (1.45 psi). The column temperature program was as follows: initial temperature: 40°C (held for 2 min), ramp rate: 8°C/min to 260°C, final temperature: 260°C (held for 1 min). The interface and ion source temperatures were maintained at 270°C. Mass spectra were acquired in electron ionization (EI) mode with an electron energy of 70 eV and an emission current of 25 μA. The scan range was set to 45–500 m/z at a scan

rate of 5 scans per second. Identification of volatile compounds was performed by comparing GC-MS spectra against reference spectra in the NIST11.L mass spectral library using computerized searches. Additionally, compound identities were further verified by referencing published mass spectra. The relative content of each volatile component was quantified using the peak area normalization method.

Expression and purification of BtsuOBPs

Full-length sequences of *BtsuOBP83a* and *BtsuOBP83b* were amplified using specific primers (Supplementary Table S1). Purified PCR products were cloned into the pGEM-T vector (Promega, USA) for sequencing verification. The confirmed target fragments were then subcloned into the pET-30a(+) expression vector (Sangon, China) using restriction enzymes (Supplementary Table S2) and T4 DNA ligase (Takara, China). Recombinant plasmids were transformed into Escherichia coli BL21 (DE3) competent cells (Tiangen, China). Bacterial cultures were grown to an OD600 of 0.6–0.8, after which protein expression was induced by adding isopropyl-β-D-thiogalactopyranoside (IPTG) (Solarbio, China) to a final concentration of 1 mM at different temperatures. Cells were harvested by centrifugation (5000 × g, 15 min), lysed in PBS buffer via sonication, and subjected to SDS-PAGE analysis after heat treatment. Large-scale protein expression was performed under optimized induction conditions. Soluble proteins in the supernatant were purified twice using Ni-affinity chromatography (GE Healthcare, USA). His-tags were removed using enterokinase (Novoprotein, China). All purification steps were conducted at 4°C. The size and purity of OBP proteins were evaluated via SDS-PAGE, and protein concentrations were determined using the bradford method [75].

Fluorescence competitive binding assays

The ligand-binding affinities of candidate OBPs were evaluated using fluorescence competitive binding assays performed on an F-380 Fluorescence Spectrophotometer (Tianfang, China), with modifications to the standard procedure. Purified OBP proteins were diluted to a final concentration of 2 μmol/L in 50 mmol/L Tris-HCl buffer (pH 7.4). The fluorescent probe N-phenyl-1-naphthylamine (1-NPN) and all candidate volatile ligands were dissolved in HPLC-grade methanol at an initial concentration of 1 mmol/L. The binding constant between OBPs and 1-NPN was determined by recording emission spectra from 350 to 470 nm at an excitation wavelength of 337 nm. To establish binding saturation, titrations were conducted by sequentially adding 1-NPN to the protein solution, reaching final concentrations of 2–20 μmol/L in 2 μmol/L increments. Fluorescence intensity was measured after stabilization, and each measurement was performed in triplicate. To assess the binding affinities of OBPs for candidate volatile ligands, competition assays were performed by introducing increasing concentrations of each ligand (final concentration range: 4–32

μmol/L, in 4 μmol/L increments). Each experiment was conducted in triplicate. Dissociation constants (*Ki*) for the volatile ligands were calculated from the corresponding *IC50* values using the following equation:

$$K_i = \frac{[IC_{50}\ ]}{1 + \frac{[1 - NPN]}{K_{1-NPN}}}$$

where [*IC50*] is the concentration of the competitor that reduces the initial fluorescence intensity by half, [1-NPN] is the free concentration of 1-NPN, and is the dissociation constant of the protein/1-NPN complex.

Protein structure prediction and molecular docking

The three-dimensional (3D) structures of OBPs and ORs were predicted using AlphaFold2 (github.com/google-deepmind/alphafold) [76], installed on a local server. The default AlphaFold2 pipeline was used for structural modeling. For ORs, we predicted the heteromeric structures of all ORs expressed in the antennae of *B. tsuneonis*, assuming a stoichiometry of two ORs and two ORco subunits. Given that these ORs can theoretically assemble in either adjacent or diagonal positions within the homotetrameric complex, we generated ten structural models for each OR-ORco complex to assess the most favorable configuration. Comparative analysis of these models indicated that the diagonal arrangement was more likely than the adjacent configuration.

The 3D structures of target ligands were downloaded from the PubChem database (https://pubchem.ncbi.nlm.nih.gov/). Binding pockets (active sites) in OBPs and ORs were predicted using DoGSite3 [77,78,79], and Grid Boxes were manually defined in PyMOL version 3.0 (http://www.pymol.org/pymol) to fully encompass the predicted binding cavities. Docking simulations were performed using AutoDock Vina version 1.2.x [80], with docking parameters optimized based on the structural characteristics of the proteins and their predicted active sites. The best docking models were selected based on binding affinity scores (kcal/mol). Protein-ligand interactions were visualized and analyzed using PyMOL. For OR models, considering the symmetrical nature of the homotetrameric OR-ORco complex, binding modes in the two OR pockets were assumed to be identical.

Molecular dynamics simulations and analysis

Molecular dynamics (MD) simulations were performed using GROMACS version 2022.3 to investigate the structural stability and molecular interactions within the system [81,82]. During small molecule preprocessing, AmberTools22 was used to assign the GAFF force field, while Gaussian 16W handled hydrogenation and RESP potential calculations. The resulting potential parameters were integrated into the molecular system's topology file. Simulations were conducted under constant

temperature (300 K) and atmospheric pressure (1 bar), employing the Amber99sb-ildn force field with TIP3P water molecules as the solvent. System neutrality was maintained by adding Na^+ ions. The simulation workflow consisted of three phases: first, energy minimization was performed using the steepest descent algorithm; second, equilibration simulations were carried out under isothermal-isovolumetric (NVT) and isothermal-isobaric (NPT) ensembles, each lasting 100 ps with a coupling constant of 0.1 ps and comprising 100,000 steps; finally, a 100 ns production simulation was conducted, consisting of 5,000,000 steps with a 2 fs time step. Post-simulation analysis was performed using built-in GROMACS tools to evaluate key dynamic properties, including root-mean-square deviation (RMSD), root-mean-square fluctuation (RMSF), and the radius of gyration. Additionally, molecular mechanics generalized Born surface area (MMGBSA) and free energy landscape analyses were conducted to further assess binding stability and conformational changes.

Results

Genome sequencing and assembly

A total of ~~24.91 Gb of Illumina short reads,~~ 37.82 Gb of clean long reads, and 58.88 Gb of raw Hi-C data were obtained for genome assembly (Supplementary Table S3). Based on k-mer analysis with k = 19, the estimated genome size of *B. tsuneonis* was approximately 324 Mb, with a heterozygosity rate of 1.61% and a repeat content of 15.1% (Fig. 1A).

At the contig level, the final draft genome assembly measured 342.91 Mb and consisted of 75 contigs with an N50 length of 11.21 Mb. This genome size aligns closely with the k-mer-based estimate (324 Mb) but is smaller than those of other Bactrocera species, such as *B. dorsalis* (530.3 Mb), *B. correcta* (702.7 Mb), *B. oleae* (468.8 Mb), *B. latifrons* (462.5 Mb), and *B. tryoni* (570.6 Mb), though slightly larger than B. *minax* (325.3 Mb) (Table 1). Notably, the *B. tsuneonis* genome exhibited a significantly higher contig N50 (11.21 Mb) than other *Bactrocera* species, including *B. minax* (27.4 kb), *B. dorsalis* (1.5 Mb), *B. correcta* (221.9 kb), *B. latifrons* (31.5 kb), and *B. tryoni* (350.9 kb) (Table 1). The GC content of B. *tsuneonis* (34.66%) was slightly lower than that of other *Bactrocera* species, which ranged from 34.5% to 36.5% (Table 1). BUSCO analysis confirmed the high completeness of the contig-level genome, with 99.5% of expected genes identified (Supplementary Table S4).

Using Hi-C to assist in genome assembly, we obtained a high-quality chromosome-level genome assembly of *B. tsuneonis* with a total size of 339 Mb (Table 1). The final assembly consisted of 24 scaffolds, with a scaffold N50 of 59.93 Mb. A total of 334.1 Mb (98.55%) of contigs were successfully anchored to six chromosomes (Fig. 1B and 1C). The chromosome count in *B. tsuneonis* (six chromosomes) is consistent with most *Bactrocera* species but differs from *B. oleae* (seven chromosomes) and *B. tryoni* (five chromosomes) (Table 1). Chromosome lengths ranged from 14.95

Mb to 78.77 Mb (Fig. 1B and 1C). BUSCO analysis further validated the completeness and accuracy of the chromosome-level genome, with 99.1% of genes successfully identified, including 98.0% single-copy genes and 1.1% duplicated genes (Supplementary Table S4). These results collectively confirm the high quality of the *B. tsuneonis* genome, making it suitable for downstream analyses.

Chromosomal synteny analysis was performed to investigate the conservation of gene order and positional relationships between *B. tsuneonis* and related species. A total of 16,964 syntenic genes were identified between *B. tsuneonis* and *B. dorsalis*, representing 62.07% of the total gene count. In contrast, only 6,693 syntenic genes were detected in *D. melanogaster*, accounting for 23.57% of its total genes. These findings suggest extensive chromosomal synteny among the three species, with a notably higher degree of genomic similarity between *B. tsuneonis* and *B. dorsalis*. As shown in Figure 1D, *B. tsuneonis* and *B. dorsalis* exhibit a high level of gene collinearity, with a substantial number of homologous genes maintaining a conserved arrangement. Additionally, Chr02 of *B. tsuneonis* and Chr04 of *B. dorsalis* exhibit collinearity with the X chromosome of *D. melanogaster*, suggesting they may represent putative sex chromosomes in these species. However, no syntenic genes associated with the *D. melanogaster* Y chromosome were identified in the six assembled chromosomes or unanchored scaffolds of *B. tsuneonis*.

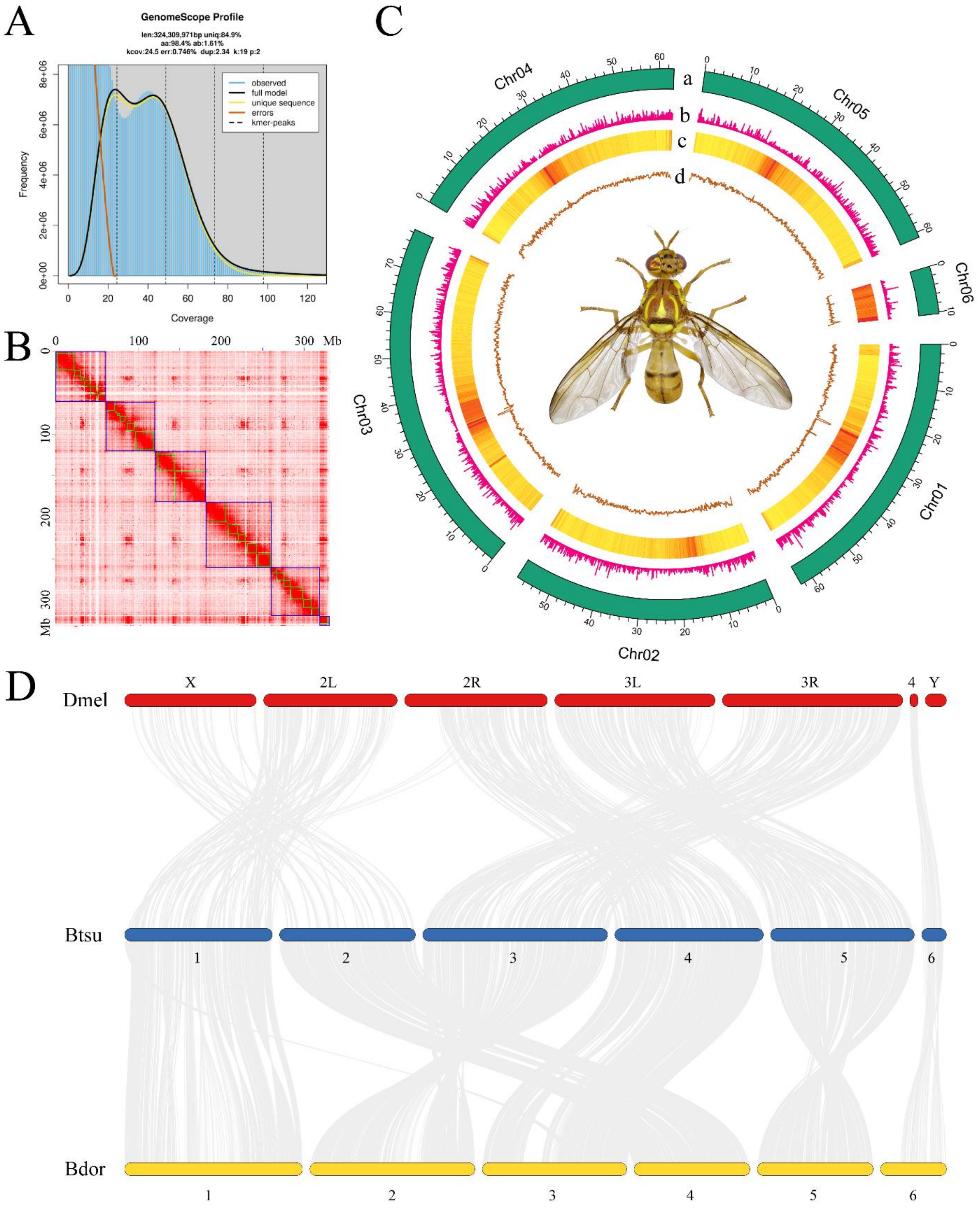

Figure 1: Genome description of *B. tsuneonis*. (A) GenomeScope estimation of genome size and heterogeneity using a k-mer of 19. (B) Hi-C interaction map produced by 3D-DNA. (C) Circular representation of the chromosomes. Tracks a–d represent the distribution of chromosome karyotypes, gene density, repeat sequences density, and GC density, respectively. (D) Synteny blocks among *B. tsuneonis* (Btsu), *D. melanogaster* (Dmel), and *B. dorsalis* (Bdor) genomes.

Table 1: Genome features of 7 *Bactrocera*

Feature	*Bactrocera tsuneonis*	*B. minax*	*B. dorsalis*	*B. correcta*	*B. oleae*	*B. latifrons*	*B. tryoni*
Assembly level	Chromosome	Scaffold	Chromosome	Chromosome	Chromosome	Scaffold	Chromosome
Genome size	339 Mb	325.3 Mb	530.3 Mb	702.7 Mb	468.8 Mb	462.5 Mb	570.6 Mb
Contig N50	11.21 Mb	27.4 kb	1.5 Mb	221.9 kb	-	31.5 kb	350.9 kb
Scaffold N50	59.93 Mb	97.4 kb	93.3 Mb	100.6 Mb	75.1 Mb	974.4 kb	81.9 Mb
Chromosomes	6	-	6	6	7	-	5
BUSCO	99.2%	98.83%	99.0%	92.6%	99.4%	99.3%	99.2%
GC content	34.66%	35%	36.5%	35.5%	35%	36%	36.5%
Protein-coding genes	13,513	21,924	14,607	17,629	12,391	12,759	14,221
Repetitive elements	24.17%	26.33%	41.57%	58.22%	45.14%	33.16%	42.84%
GenBank	GCA_046562955.1	GCA_029783545.1	GCA_023373825.1	GCA_027475135.1	GCA_042242935.1	GCA_001853355.1	GCA_016617805.2

Genome annotation

In the assembled *B. tsuneonis* genome (339 Mb), 24.17% of the sequences were identified as repetitive elements. This proportion is lower than that observed in other *Bactrocera* species, including *B. minax* (26.33%) and *B. latifrons* (33.16%), and substantially lower than *B. correcta* (58.22%), *B. oleae* (45.14%), *B. dorsalis* (41.57%), and *B. tryoni* (42.84%) (Table 1). Among the transposable elements, long interspersed nuclear elements (LINEs) accounted for 1.82%, long terminal repeat (LTR) elements comprised 1.63%, and DNA transposons constituted 4.63% of the genome. Additionally, 203,409 simple repeat elements were identified, representing 2.72% of the *B. tsuneonis* genome (Supplementary Table S5).

Protein-coding genes in the *B. tsuneonis* genome were predicted using a combination of three approaches: de novo prediction, homology-based prediction, and RNA-seq-supported annotation. A total of 14,529 protein-coding genes were identified, supported by all three methods. This number is

lower than that of *B. dorsalis* (14,607), *B. correcta* (17,629), and *B. tryoni* (14,221) and is significantly reduced compared to the closely related species *B. minax* (21,924) (Table 1). Functional annotation of the predicted genes revealed that 12,969 (89.26%), 8,926 (60.31%), and 10,483 (72.15%) genes matched entries in the NR, SwissProt, and Pfam databases, respectively. Additionally, 9,073 genes (62.45%) were assigned GO terms, while 7,539 (51.89%) were mapped to KEGG pathways. Overall, 13,513 genes (93.01% of the total protein-coding genes) were successfully annotated across all databases (Supplementary Table S6).

Orthology prediction and inference of phylogenetic relationships

Orthologous gene analysis was conducted on *B. tsuneonis*, its closely related species *B. minax*, the model species *D. melanogaster*, and 14 additional species from the Tephritidae family (Fig. 2; Supplementary Table S7). Gene family clustering was categorized into four groups: single-copy genes, multiple-copy genes, species-specific genes (unique genes), and unassigned genes. OrthoFinder analysis clustered 367,296 genes from 17 species into 33,010 unique gene families (orthogroups). Phylogenetic reconstruction based on single-copy orthologous genes revealed that all *Bactrocera* species formed a distinct clade. For *B. tsuneonis*, 14,529 genes were assigned to 12,543 gene families, including 23 species-specific genes. Divergence time estimation using MCMCTree suggested that *B. tsuneonis* and *B. minax* diverged approximately 4.3 Mya. The split between *Bactrocera* and *Zeugodacus* was estimated at around 59.3 Mya (Fig. 2).

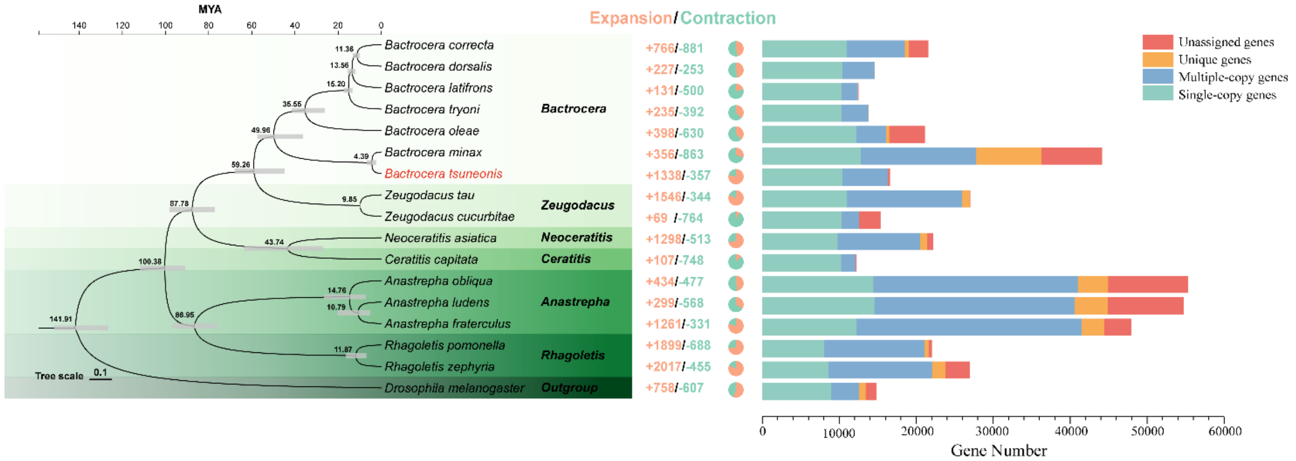

Figure 2: Phylogenetic tree with the dynamic evolution of gene families among *B. tsuneonis*, *B. minax*, and other species.

Gene families associated with adaptability and invasiveness

Using CAFE, we analyzed gene family expansion and contraction during the evolutionary process of *B. tsuneonis*. A total of 559 gene families were found to be expanded, while 1,166 gene families underwent contraction (Fig. 2). Functional annotation with KinFin revealed that chemosensory-related genes, including ORs, OBPs, and GRs, were predominantly found in the contracted gene

families. In contrast, genes associated with detoxification and metabolism, such as CYP450s and ABCs, were enriched in the expanded gene families. These evolutionary changes suggest that adaptations in these gene families have played a critical role in the invasion and ecological adaptation of *B. tsuneonis*. Based on these results, we further examined multiple gene families associated with environmental adaptability, including chemosensory-related genes (OBPs, ORs, IRs, GRs, and CSPs), heat shock proteins (HSPs), and detoxification-related genes (ABCs, GSTs, P450s, UGTs, and CCEs) (Fig. 3). The identification of these gene families provides insights into the genetic mechanisms underlying insect adaptation, which are essential for their survival and ecological success.

To mitigate the effects of harmful substances such as plant secondary metabolites and pesticides, insects have evolved a sophisticated detoxification system. The major detoxification enzymes include CYP450s and GSTs, alongside additional functional gene families such as CCEs, UGTs, and ABCs [83,84,85]. In the *B. tsuneonis* genome, we identified 119 CYP450s, 65 ABCs, 30 UGTs, 40 GSTs, and 7 CCEs (Fig. 3A; Supplementary Table S8). Compared to other *Bactrocera* species, *B. tsuneonis* has a similar number of GSTs and UGTs, while its CCEs and CYP450s counts are lower. However, the number of ABCs is slightly higher. Heat shock proteins play a crucial role in enabling insects to tolerate environmental stressors such as extreme temperatures, oxidative stress, and heavy metal exposure [86,87,88]. In *B. tsuneonis*, we identified five HSP subfamilies, comprising a total of 91 HSP genes: 19 HSP20s, 41 HSP40s, 10 HSP60s, 18 HSP70s, and 3 HSP90s (Fig. 3A; Supplementary Table S8). Compared to other *Bactrocera* species, *B. tsuneonis* exhibits a higher number of HSP genes.

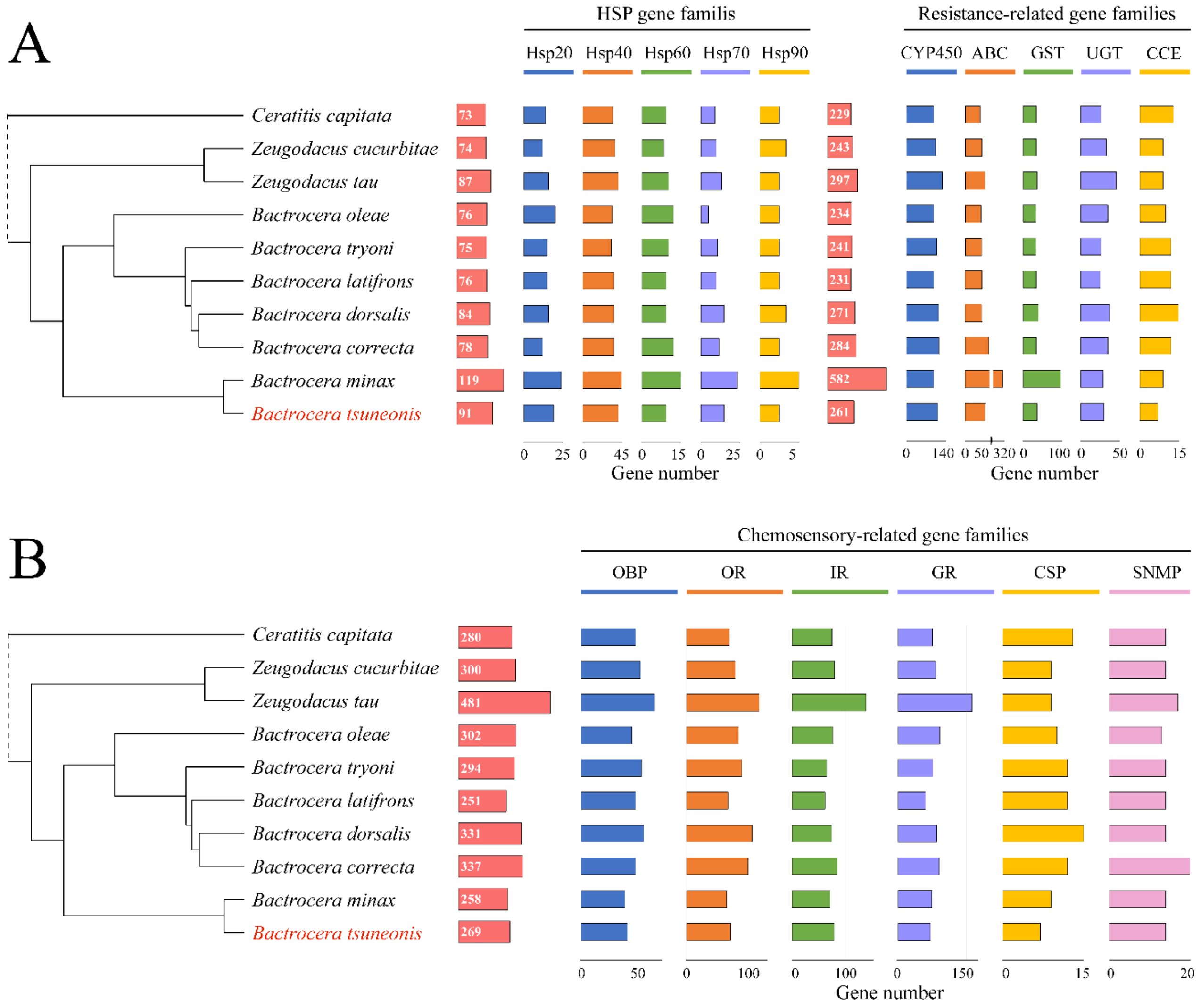

Figure 3: Comparison of gene numbers of (A) heat shock protein gene families, detoxification-related gene families and (B) chemosensory-related gene families in *B. tsuneonis*, and other species.

Genes associated with chemosensory systems

Chemosensory-related gene families in insects include OBPs, GRs, ORs, IRs, CSPs, and SNMPs. These gene families are essential for key behaviors such as feeding, mating, and predator avoidance [89,90,91]. In this study, we identified six chemosensory-related gene families in the *B. tsuneonis* genome, revealing a significant reduction in OBP, OR, GR, and CSP gene counts compared to other *Bactrocera* species, whereas IR and SNMP gene numbers remained relatively stable. Specifically, we identified 39 OBPs, 68 ORs, 79 IRs, 62 GRs, 7 CSPs, and 14 SNMPs in *B. tsuneonis* genome (Fig. 3B; Supplementary Table S8).

Phylogenetic analysis (Fig. 4A) of OBP genes in *B. tsuneonis*, *D. melanogaster*, and *B. correcta*. It is evident that the oligophagous *B. tsuneonis* has significantly fewer OBP genes than the polyphagous *B. correcta*. The OBPs of *B. correcta* are more closely related to those of *D. melanogaster*, such as Dmellush, a protein known to be involved in pheromone-binding activity [92], which was not

identified in *B. tsuneonis*. Additionally, OBP genes of the same species do not cluster into species-specific branches but instead cluster based on different subfamilies. Classical OBPs are distributed across different evolutionary clades. Similarly, phylogenetic analysis of OR genes (Fig. 4B) suggests that the OR gene family in *B. tsuneonis* has undergone large-scale contraction compared to *B. correcta*. Notably, in the OR-VI and OR-VII groups, we observed specific contractions in *B. tsuneonis* compared to *B. correcta*, particularly in OR7a and OR59a. These findings suggest that *B. tsuneonis* has experienced selective gene losses in key chemosensory gene families, potentially influencing its host plant specificity and ecological adaptations.

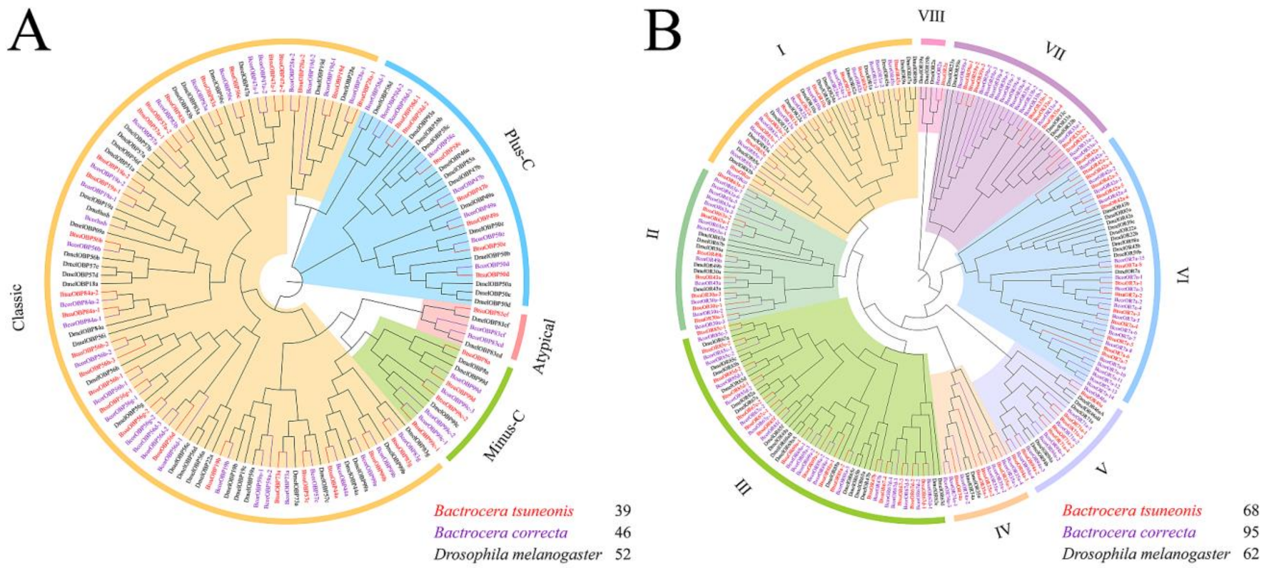

Figure 4: Phylogenetic relationships of *Bactrocera tsuneonis* (Btsu) (A) odorant-binding protein (OBP), (B) odorant receptor (OR) in comparison with *Bactrocera correcta* (Bcor) and *Drosophila melanogaster* (Dmel).

A total of 55.37 Gb of clean reads were obtained through sequencing and used for tissue-specific expression analysis. The expression profiles of *BtsuOBPs* across different body parts of male and female adult flies were visualized using a heatmap (Fig. 5A). The results revealed that *BtsuOBPs* exhibit broad expression patterns, indicating that many OBPs are not restricted to a single body part. Notably, similar expression trends were observed between males and females for the same *BtsuOBPs*. Among the identified OBPs, 13 *BtsuOBPs* displayed relatively high expression levels in the antennae, while five exhibited elevated expressions in the ovipositor. Several *BtsuOBPs*, including *BtsuObp99b*, *BtsuOBP28a-1*, and *BtsuOBP83g*, were also expressed in the legs. Notably, *BtsuOBP83a* and *BtsuOBP83b* demonstrated the highest expression levels in the antennae (Fig. 5C), suggesting their essential roles in olfactory perception.

Similarly, transcriptome analysis of *BtsuORs* revealed distinct expression patterns across various body parts in adult male and female flies. As shown in Figure 5B, most *BtsuORs* were predominantly

expressed in the antennae, while only a few were detected in the legs or ovipositor. These findings highlight the central role of ORs in antennal-mediated olfactory function in *B. tsuneonis*. To further verify the transcriptome-based expression profiles, qRT-PCR was performed using antennal RNA for five OBP genes and five OR genes that showed high antennal expression in RNA-seq data. The qRT-PCR results exhibited expression patterns consistent with the transcriptomic analysis, showing a strong correlation between the two datasets (Supplementary Fig. S1) and supporting the reliability of the RNA-seq–based gene expression profiles.

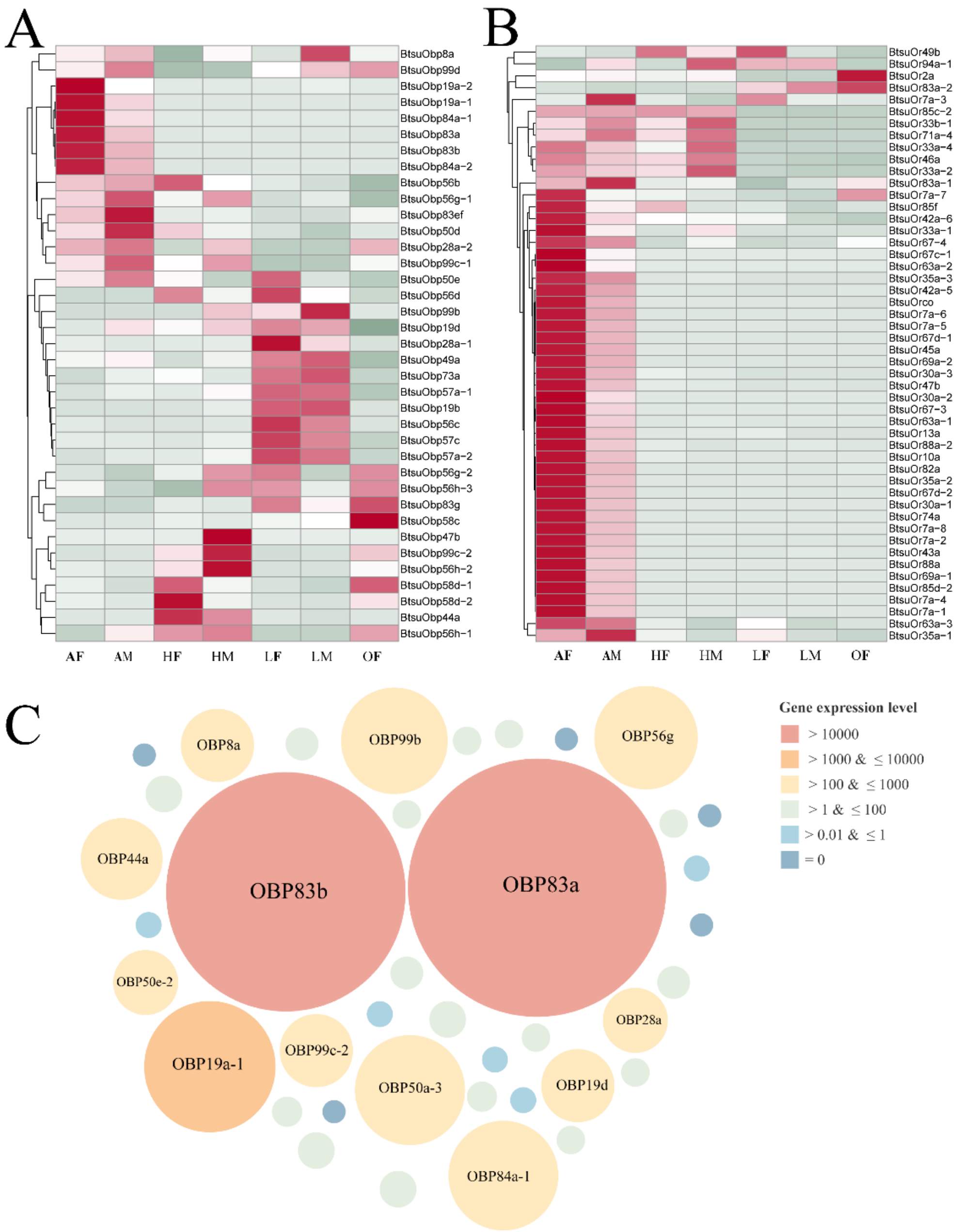

Figure 5: Expression pattern analysis of genes in different tissue (AF: Female adult antennae; AM: Male adult antennae; HF: Female adult head without antennae; HM: Male adult head without antennae; LF: Female adult leg; LM: Male adult leg; OF: Female adult ovipositor). (A) BtsuOBPs in different tissue, (B) BtsuOrs in different

tissue, (C) BtsuOBPs in antennae.

Ligand-binding properties of BtsuOBPs

HS-SPME-GC-MS was employed to identify and quantify volatile compounds emitted by *Maoping Tangerine*, the primary host of *B. tsuneonis*. This method enabled comprehensive profiling of both the composition and concentration of volatiles. A total of 113 volatile compounds were detected in *Maoping Tangerine* (Supplementary Table S9). In the previous study, we completed the identification of volatile compounds from non-host fruits of *B. tsuneonis*, including guava, mango, and apple (Supplementary Fig. S2). To further investigate potential olfactory cues, volatile compounds from *Maoping Tangerine* were compared with those of non-host fruits (Supplementary Fig. S2 and S3). This comparative analysis identified 43 candidate volatiles, including 10 from *Maoping Tangerine* and 33 from non-host plants, as potential ligands for further study. (Supplementary Table S10).

To explore the olfactory mechanisms of *B. tsuneonis*, BtsuOBP83a and BtsuOBP83b, which exhibited high expression levels in the antennae, were selected for functional analysis. Recombinant proteins for these two BtsuOBPs were successfully expressed in vitro, and their purity and molecular size were confirmed via SDS-PAGE (Supplementary Fig. S4). Competitive binding assays using 1-NPN as a fluorescent probe were conducted to assess the binding affinities of BtsuOBPs to 43 selected volatile compounds derived. First, the affinity constants of BtsuOBPs for 1-NPN were determined. Both proteins exhibited characteristic saturation binding curves with 1-NPN, and their Scatchard plots were linear (Supplementary Fig. S5). The dissociation constants (*Kd*) were calculated as 5.48 μM for BtsuOBP83a and 6.92 μM for BtsuOBP83b, confirming 1-NPN as a suitable fluorescent probe for these proteins. Among the 43 tested volatiles, BtsuOBP83a exhibited specific binding affinity to two host-derived volatiles while showing weak binding to non-host-derived volatiles. In contrast, BtsuOBP83b displayed weak binding across all tested compounds. (Fig. 6 and Supplementary Fig. S6; Supplementary Table S11). These findings suggest that BtsuOBP83a may play a crucial role in hosts volatile compounds binding in *B. tsuneonis*.

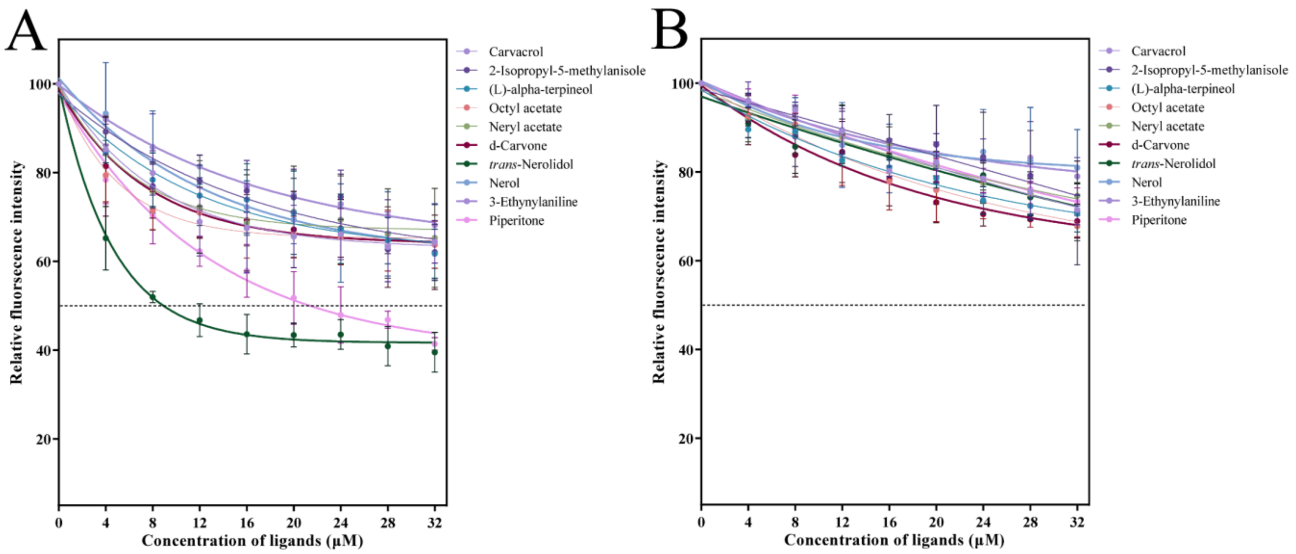

Figure 6: Comparison of binding properties of (A) BtsuOBP83a and (B) BtsuOBP83b with respect to *Maoping Tangerine*.

Structural prediction and molecular docking

The 3D structure of BtsuOBP83a was predicted using AlphaFold 2.0 (Fig. 7A), and the model was assessed as reliable. The predicted structure exhibited six characteristic α-helices and a hydrophobic binding cavity, consistent with the typical structural features of insect odorant-binding proteins (OBPs). Structural analysis combined with molecular docking revealed that BtsuOBP83a possesses a binding cavity capable of interacting with two ligands—*trans*-nerolidol and piperitone—through a combination of hydrophobic interactions and hydrogen bonding (Fig. 7B).

For *trans*-nerolidol, binding was facilitated by hydrophobic interactions with residues PHE22, LEU58, ILE62, LEU76, VAL84, LEU88, TRP114, PHE123, and PRO125, alongside hydrogen bonding with PHE123 (Fig. 7C). Piperitone exhibited hydrophobic interactions with residues LEU76, LEU88, TRP114, TYR122, and PHE123 (Fig. 7D). Molecular docking results indicated low binding energies for *trans*-nerolidol (-6.71 kcal/mol) and piperitone (-6.317 kcal/mol), suggesting strong interactions between BtsuOBP83a and these ligands. These findings highlight BtsuOBP83a as a key OBP involved in host volatile recognition in *B. tsuneonis*.

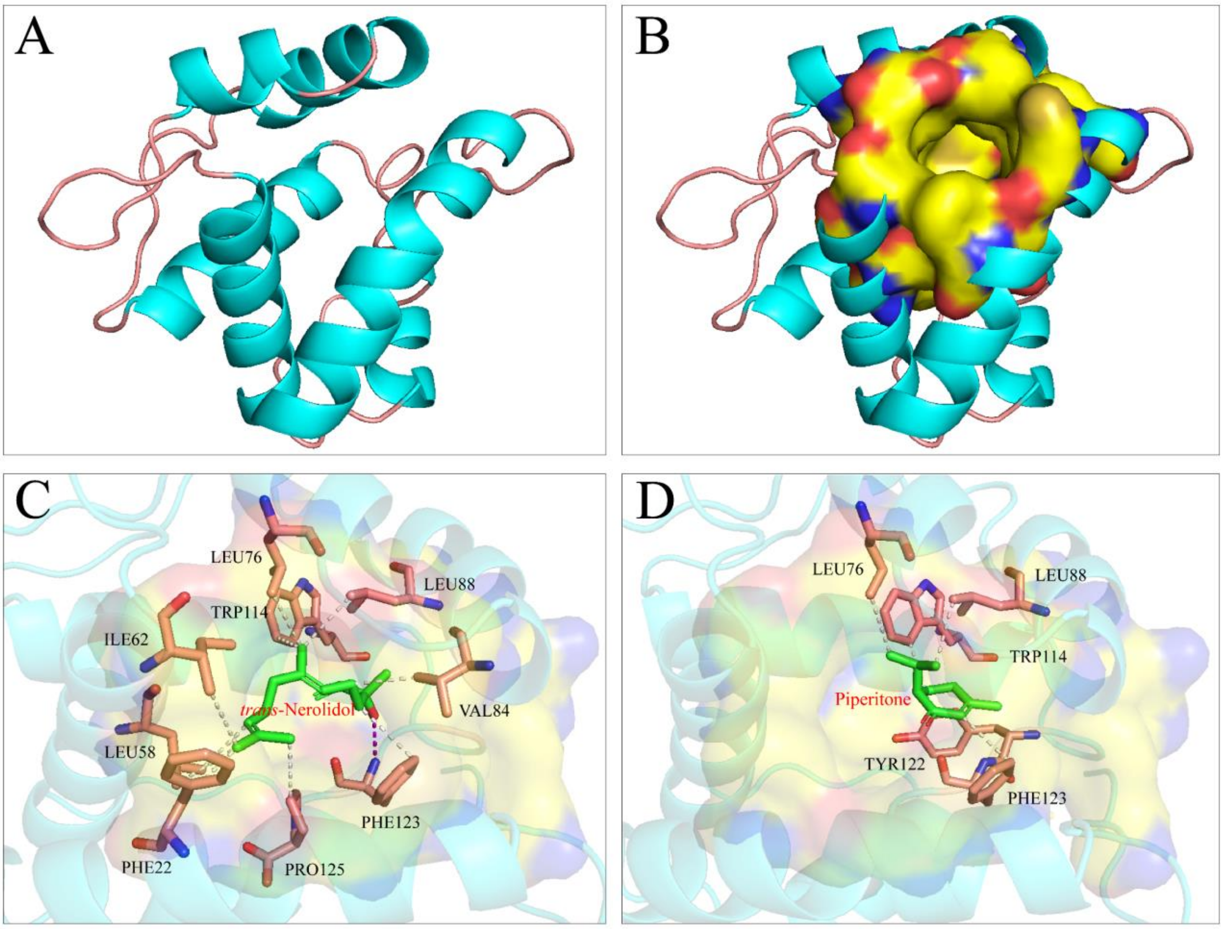

Figure 7: Molecular docking of BtsuOBP83a. (A) Predicted 3D structure, (B) the binding cavity, key residues with respect to (C) *trans*-nerolidol, and (D) piperitone.

To further explore the interactions between key ligands and odorant receptors (ORs) in *B. tsuneonis*, we utilized AlphaFold 2.0 to predict the heteromeric structures of OR-ORco complexes. Molecular docking analyses were performed using the AlphaFold-predicted diagonal heteromeric complex (Fig. 8A and 8B) to assess ligand binding interactions within OR-ORco heterotetramers (Supplementary Table S12). Among the 68 ORs analyzed, BtsuOr7a-6 exhibited the lowest binding energy with *trans*-nerolidol (-7.381 kcal/mol), while BtsuOr7a-4 showed the strongest affinity for piperitone (-6.904 kcal/mol) (Supplementary Fig. S7).

Molecular dynamics simulations

The interaction between *trans*-nerolidol and BtsuOr7a-6 demonstrated remarkable stability and specificity (Fig. 8 and Supplementary Fig. S8). Root mean square deviation (RMSD) analysis showed that the overall protein structure stabilized after 40 ns, with only minor fluctuations. The ligand RMSD remained steady at approximately 0.1 nm, indicating minimal movement within the binding pocket while maintaining a stable interaction. The RMSD of the protein-ligand complex stabilized at 0.55 nm, further supporting the conformational stability of the system. Root mean square fluctuation

(RMSF) analysis revealed minimal fluctuations in the binding site residues, suggesting limited dynamic behavior in the binding pocket, while non-binding regions exhibited greater fluctuations without affecting overall binding stability. MM/PBSA calculations estimated a binding free energy (ΔGMMGBSA) of -37.11 kcal/mol, indicating high thermodynamic stability. The interaction was primarily driven by van der Waals forces (ΔVDWAALS = -38.93 kcal/mol) and nonpolar solvation energy (ΔESURF = -5.61 kcal/mol). Solvent-accessible surface area (SASA) analysis showed a decrease from 900 nm^2 to below 800 nm^2 upon ligand binding, suggesting that *trans*-nerolidol was stably embedded within the binding site, further enhancing the specificity of the interaction.

The binding of piperitone to BtsuOr7a-4 exhibited high stability and adaptability (Fig. 8 and Supplementary Fig. S9). RMSD analysis indicated that the overall protein structure stabilized after 20 ns, with a slight increase in complex RMSD after 80 ns. The ligand RMSD remained consistently around 0.04 nm, reflecting a highly stable binding position. Dynamic evaluations of the binding site showed minimal fluctuations in the core binding residues, while non-binding regions exhibited greater flexibility without compromising overall binding stability. MM/PBSA calculations estimated a binding free energy (ΔGMMGBSA) of -23.56 kcal/mol, with van der Waals forces (ΔVDWAALS = -26.55 kcal/mol) as the primary driving force. SASA analysis revealed a decrease from 860 nm^2 to 750 nm^2, indicating that piperitone was securely embedded within the receptor.

Comprehensive analysis suggests that the binding of BtsuORs to *trans*-nerolidol and piperitone is primarily driven by hydrophobic interactions. Core binding pocket residues, such as VAL197 in BtsuOr7a-6 and LEU195 in BtsuOr7a-4, play crucial roles in stabilizing ligand interactions (Fig. 8). Among the two receptors, BtsuOr7a-6 exhibited the strongest binding affinity with *trans*-nerolidol (ΔGMMGBSA = -37.11 kcal/mol), primarily driven by van der Waals interactions. The larger binding pocket of BtsuOr7a-6 likely provides greater accommodation capacity, enhancing its adaptability. Conversely, BtsuOr7a-4 demonstrated the highest binding stability with piperitone, with ligand RMSD consistently below 0.05 nm and minimal fluctuations in the binding site, suggesting that piperitone may serve as a specific ligand for BtsuOr7a-4.

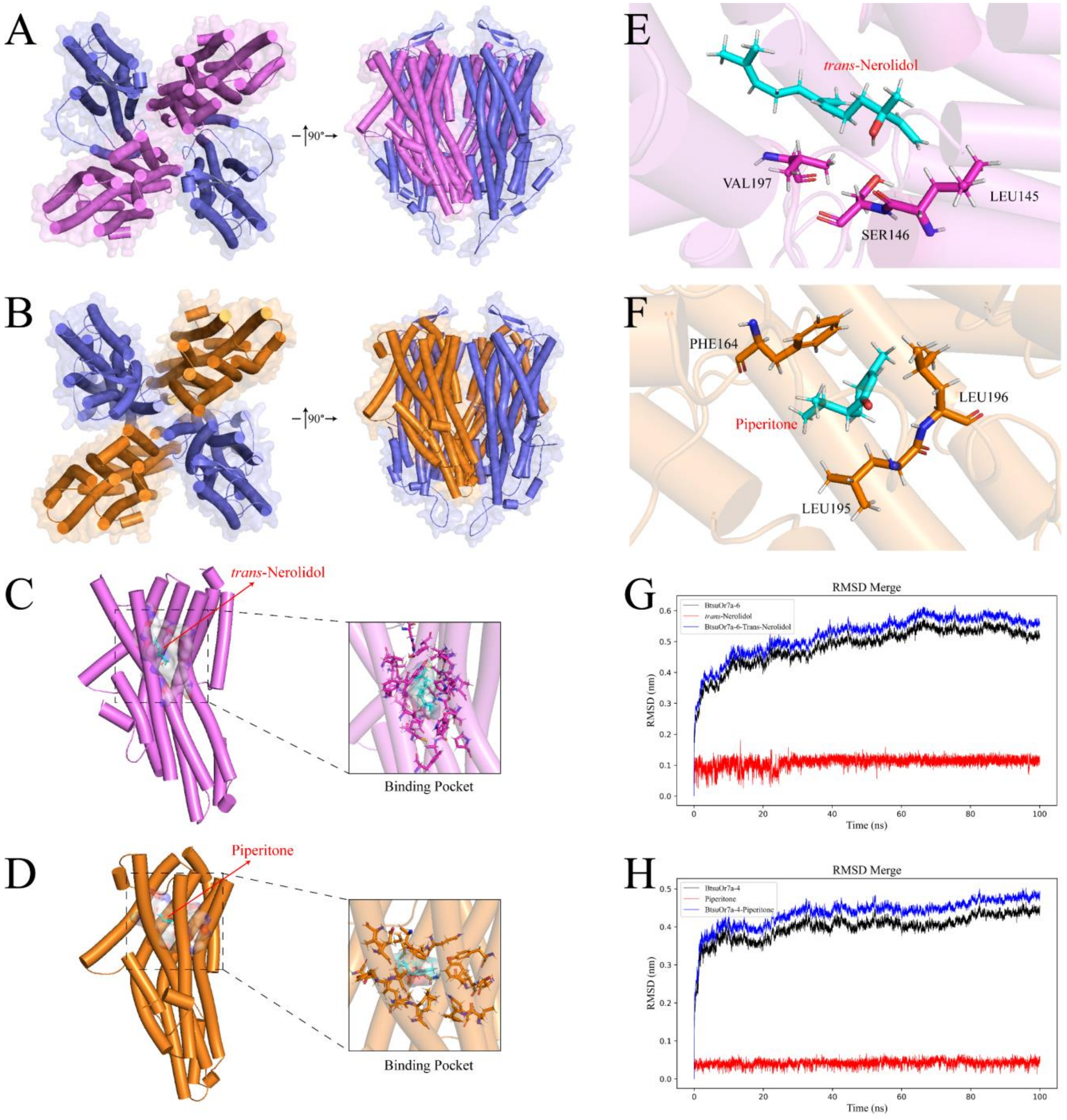

Figure 8: Molecular dynamics simulations of substrate-bound BtsuORs. Representative structure of the heteromeric structures of (A) BtsuOR7a-6, (B) BtsuOR7a-4 in complex with BtsuORco in a stoichiometry of two ORs and two ORcos predicted, Close-up view of the docking structure of BtsuORs in complex with (C) *trans*-nerolidol, (D) piperitone , Structural snapshots to show the interactions between BtsuORs and (E) *trans*-nerolidol, (F) piperitone, RMSD analysis of BtsuORs in complex with (G) *trans*-nerolidol, (H) piperitone.

Discussion

Genome offers a foundation for studying host specialization and adaptation

The chromosome-level genome assembly of *B. tsuneonis* provides a crucial foundation for understanding the genetic basis of its host specialization and ecological adaptation. Compared to other

Bactrocera species, *B. tsuneonis* exhibits a relatively small genome size (339 Mb) with a lower proportion of repetitive elements (24.17%). This is consistent with previous studies showing that genome size can be influenced by the proportion of transposable elements and repeat sequences, which vary across insect species depending on their evolutionary history and ecological adaptations [93,94,95]. The high contig N50 (11.21 Mb) and scaffold N50 (59.93 Mb) values indicate a well-assembled and contiguous genome, facilitating downstream functional and comparative genomic analyses.

A well-assembled genome is essential for understanding the genetic mechanisms underlying insect specialization. Previous studies on *B. dorsalis* and *B. correcta* have shown that genome structure plays a role in determining ecological plasticity and host adaptability [96,97]. The high completeness (99.2% BUSCO) of *B. tsuneonis* further supports the reliability of this assembly, and accurate gene annotation and functional analysis have been conducted. The availability of this reference genome serves as a valuable resource for investigating the molecular basis of host selection, ecological divergence, and evolutionary trajectories within *Bactrocera* species.

Gene contractions in olfaction highlight adaptation to host

Comparative genomic analysis revealed significant contractions in the chemosensory gene families of *B. tsuneonis*, particularly in OBPs and ORs. These gene families play a critical role in insect olfactory perception and host recognition, facilitating the detection of plant volatiles and pheromones [98,99]. In the genome of *B. tsuneonis*, these gene families have undergone a significant reduction compared to polyphagous *Bactrocera* species (Fig. 4), a trend that has also been observed in other oligophagous insects [100,101]. This contraction may enhance the ability of *B. tsuneonis* to accurately identify and select its host, thereby improving its ecological adaptability. We hypothesize that the contraction of OR genes may play a vital role in population establishment and the rapid spread of invasive species, facilitating efficient host localization and optimizing foraging and reproductive strategies.

Despite the reduction in chemosensory genes, detoxification-related gene families, including CYP450s, ABCs, and GSTs, exhibited expansion in *B. tsuneonis*. During our gene family analysis, an abnormal increase in the number of detoxification-related genes was observed in *B. minax*. Therefore, in this study, we did not include a comparison of detoxification-related genes between *B. minax* and *B. tsuneonis*. These gene families are essential for metabolizing plant secondary metabolites and insecticides [102]. The expansion of detoxification genes in *B. tsuneonis* may reflect an evolutionary adaptation to citrus-derived allelochemicals, providing enhanced metabolic resistance to host-specific phytotoxins.

Tissue-specific expression of OBPs and ORs highlights their roles in olfactory perception

Genomic studies have revealed that the olfactory system of *B. tsuneonis* consists of 39 OBPs and 68 ORs, highlighting the complexity of its chemosensory system [96,97]. To further investigate the roles of these genes in olfactory function and oviposition preference, this study analyzed their transcriptional levels across various tissues, including the antennae, head (excluding antennae), legs, and ovipositor. The results indicated distinct expression patterns of OBPs and ORs in different body parts of *B. tsuneonis*, emphasizing their specialized roles in olfactory perception (Fig. 3). OBPs exhibited broad expressions, with significant enrichment in the antennae, ovipositor, and legs. 13 *BtsuOBPs* were highly expressed in the antennae, reinforcing the antennae's role as the primary olfactory organ for detecting volatile compounds. These OBPs are likely involved in binding and transporting environmental volatiles to ORs, thereby initiating olfactory signal transduction cascades [103,104]. Additionally, several *BtsuOBPs* were expressed in the legs, suggesting potential roles beyond olfactory perception. For instance, *BdorOBP28a-2* expression in the legs has been implicated in *B. dorsalis* resistance to malathion [105].

In contrast, OR genes were predominantly expressed in the antennae, consistent with their function in olfactory signal transduction [106]. Previous studies have demonstrated that ORs are essential for recognizing specific host-associated volatiles and play a critical role in mediating insect behavior [12,107]. The limited expression of ORs in non-olfactory tissues suggests that their primary function is in odor recognition. Interestingly, both OBPs and ORs were detected in the ovipositor, suggesting a potential role in oviposition site selection. Olfactory genes are expressed in reproductive tissues, likely contributing to host recognition during oviposition [108]. The high expression levels of OBPs and ORs in the antennae particularly emphasize their importance in volatile perception, aiding fruit flies in recognizing host fruits and potentially detecting ecological risks or competitive pressures associated with specific odors.

BtsuOBP83a mediates host selection by binding key volatiles

Transcriptomic analysis revealed that *BtsuOBP83a* and *BtsuOBP83b* exhibited the highest expression levels in female antennae, underscoring their critical role in olfactory perception (Fig. 3C). To investigate their function in host volatile recognition, we assessed the binding ability of these two highly expressed OBPs to 10 host-derived volatile compounds and 33 non-host-derived volatile compounds. Fluorescence competitive binding assays demonstrated that *BtsuOBP83a* selectively binds to two host-specific volatiles, *trans*-nerolidol and piperitone, suggesting that these compounds serve as key olfactory cues for *B. tsuneonis*. OBPs play a crucial role in odorant transport and are essential for facilitating ligand-receptor interactions within the insect olfactory system [16,19]. The strong binding affinity observed in this study supports the hypothesis that *BtsuOBP83a* serves as a key mediator in host odor recognition for *B. tsuneonis*, playing an important ecological role in host

selection and reproductive behavior. Compared to polyphagous species, *B. tsuneonis* has fewer OBPs, which may indicate an evolutionary trade-off that prioritizes specificity in odor detection over diversity.

According to previous studies, host preference in both specialists and generalists is primarily influenced by visual and olfactory cues [100]. BtsuOBP83a exhibited specific binding affinity to two host-derived volatiles while showing weak binding to non-host-derived volatiles, further supporting the idea that specialist insects rely on an olfactory system to accurately detect and respond to host-specific chemical cues. However, the preference for *Maoping Tangerine* as the primary host may limit the adaptability of *B. tsuneonis*, making it more vulnerable to environmental fluctuations and changes in host availability. To further elucidate the molecular recognition mechanisms underlying these preferences, we conducted molecular docking analyses to examine the interactions between *BtsuOBP83a* and several host volatiles. The lower binding energies observed in these analyses indicate stronger ligand-protein interactions, supporting the fluorescence binding assay results and validating the structural basis of these interactions. Future behavioral assays, such as Y-tube olfactometer and oviposition preference tests, will be valuable for linking OBP – ligand binding profiles to actual host-seeking behaviors, and for confirming the ecological relevance of key volatiles identified in this study.

BtsuOr7a-6 and BtsuOr7a-4 mediate host recognition by detecting key volatiles

To further investigate the interactions between the 2 volatile compounds and ORs in *B. tsuneonis*, we utilized AlphaFold2 structural prediction, molecular docking, and MD simulations. Based on AlphaFold2 predictions, OR and ORco are hypothesized to assemble into a heterotetrameric structure, $(OR)_2$–$(ORco)_2$, with two possible structural arrangements: adjacent and diagonal configurations [109]. In this study, 10 models were generated for each configuration, with the majority adopting a diagonal arrangement, suggesting that this conformation may be more stable in fruit flies. Molecular docking experiments identified *BtsuOr7a-6* and *BtsuOr7a-4* as the key receptors with the lowest binding free energy for *trans*-nerolidol and piperitone, respectively, among the 68 OR candidates. These findings suggest that these ORs are the primary receptors involved in detecting these volatiles. Previous studies have demonstrated that the OR7a family is essential for the detection of chemical signals commonly recognized by *D. melanogaster* [110]. ORs are key determinants of odor coding, and their ligand specificity directly influences insect behavior [111,112]. The low binding energy values obtained from molecular docking indicate that these ORs have evolved to detect specific citrus volatiles with high sensitivity, reinforcing their role in host location.

Molecular dynamics simulations further validated the binding stability of the two volatiles with their respective ORs. Core residues within the binding pocket, such as VAL197 and LEU195, provided

the primary driving force through hydrophobic interactions. These results reveal the high adaptability of the OR binding pocket for hydrophobic volatiles and the crucial role of polar residues in recognizing complex ligands. The highly stable binding of *BtsuOr7a-6* to *trans*-nerolidol suggests that this receptor plays a central role in mediating host attraction, while the specificity of *BtsuOr7a-4* for piperitone indicates its involvement in detecting additional host-related cues. These findings support the idea that ORs drive host localization and reproductive behaviors in fruit flies by recognizing host-specific volatiles [113]. Additionally, the contraction of OR genes in *B. tsuneonis* reflects an adaptation toward detecting a narrow yet ecologically relevant set of host volatiles, further supporting its olfactory specialization. To further confirm the ligand-receptor relationships predicted here, future work will employ heterologous expression systems to functionally characterize these ORs and directly validate their roles in odorant perception.

Oligophagy and its evolutionary significance in insects

Oligophagy, or the specialization of insects on a narrow range of host plants, represents a distinct evolutionary strategy that contrasts with polyphagy, where insects feed on a broad spectrum of plant species. Oligophagous insects, such as *B. tsuneonis*, exhibit strong host specificity, often displaying precise adaptations in their chemoreception, detoxification mechanisms, and behavioral strategies. The evolutionary drivers and consequences of oligophagy have been widely studied in insect ecology and evolutionary biology, highlighting the trade-offs between host specialization and ecological flexibility [114,115]. One of the key adaptations associated with oligophagy is the reduction in the number of olfactory-related genes, as specialized herbivorous insects typically evolve a more selective olfactory repertoire. [100,101]. This mechanism provides an advantage in locating suitable host plants within complex environments but comes at the cost of reduced adaptability to new or alternative hosts. In this study, the contraction of OR genes and the functional analysis of OBPs in *B. tsuneonis* further support this pattern, as they prioritize sensitivity to a limited set of ecologically relevant chemical signals.

Despite the ecological advantages of host specialization, oligophagy imposes inherent constraints. Oligophagous insects are more vulnerable to fluctuations in host availability, environmental changes, and habitat disturbances, making them potentially more susceptible to population declines under unfavorable conditions [116,117]. Additionally, their evolutionary flexibility is restricted, as they are less capable of shifting to new hosts compared to polyphagous species. However, in stable environments where host plants are abundant, oligophagy can be a highly successful strategy, allowing insects to avoid interspecific competition and optimize feeding efficiency [118]. Overall, the evolution of oligophagy reflects a trade-off between ecological specialization and adaptability. Insect species that exhibit oligophagy, such as *B. tsuneonis*, have fine-tuned their olfactory and

detoxification systems to maximize efficiency in host detection and utilization. Future research should continue to explore the genetic and ecological mechanisms underpinning oligophagy, particularly in pest species, to improve our understanding of host-insect interactions and inform pest management strategies.

Conclusions

This study presents the first high-quality chromosome-level genome assembly of *B. tsuneonis*, revealing key genomic adaptations underlying its host specificity. Comparative genomic analysis identified a significant contraction in chemosensory gene families, particularly OBPs and ORs, consistent with its oligophagous nature. Functional assays confirmed that *BtsuOBP83a* binds strongly to host volatiles *trans*-nerolidol and piperitone, while *BtsuOr7a-6* and *BtsuOr7a-4* serve as key receptors for host odor recognition. These interactions, primarily driven by hydrophobic forces, reveal the structural basis of host recognition in *B. tsuneonis*. This study provides insights into the molecular mechanisms of host selection in *B. tsuneonis* and contributes valuable genomic evidence on olfactory adaptation in oligophagous insects. Future research should validate these key genes in vivo and explore behavior-based pest control strategies for more precise and sustainable management of fruit flies.

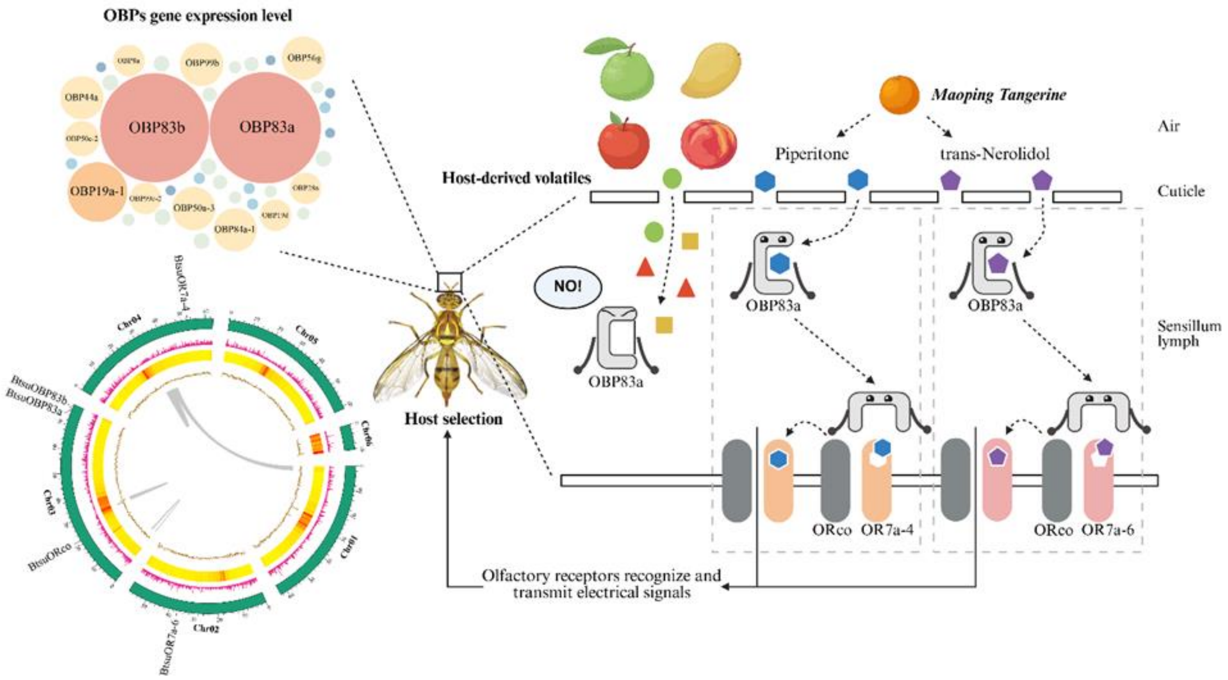

Figure 9: Schematic diagram of genome assembly, OBP expression level in antennae, and olfactory protein recognition mechanism of host volatiles in *B. tsuneonis*.

Additional Files

Supplementary Fig. S1. Correlation between gene expression levels from RNA-seq and RT-qPCR.

Supplementary Fig. S2. Gas chromatograph-mass spectrometry analyses (GC-MS) of host fruits (guava, mango, and apple from the previous study).

Supplementary Fig. S3. Gas chromatograph-mass spectrometry (GC-MS) analyses without fruit.

Supplementary Fig. S4. SDS-PAGE analysis of recombinant target proteins. (M: Molecular Weight Marker; A: Protein Sample After Enterokinase Cleavage; B: Target Protein After Enterokinase Cleavage; C: PBS Elution Fraction; D: 500 mM Imidazole Elution Fraction).

Supplementary Fig. S5. Binding curves and Scatchard plots of the fluorescence probe 1-NPN to BtsuOBPs.

Supplementary Fig. S6. Comparison of binding properties of (A) BtsuOBP83a and (B) BtsuOBP83b with respect to guava, mango, apple.

Supplementary Fig. S7. Key residues of BtsuORs with respect to trans-nerolidol and piperitone.

Supplementary Fig. S8. RMSF and SASA analysis of BtsuOR7a-6 in complex with trans-nerolidol.

Supplementary Fig. S9. RMSF and SASA analysis of BtsuOR7a-4 in complex with piperitone.

Supplementary Table S1. Primers used in this study.

Supplementary Table S2. Restriction enzymes used in this study.

Supplementary Table S3. Statistics of sequencing data of *Bactrocera tsuneonis* genome.

Supplementary Table S4. Completeness of *Bactrocera tsuneonis* genome assembly and annotation evaluated by BUSCO based on insecta_odb10 database.

Supplementary Table S5. Statistics for repeat elements in the genome of *Bactrocera tsuneonis*.

Supplementary Table S6. Functional annotation statistics of *Bactrocera tsuneonis* genome.

Supplementary Table S7. Information regarding gene family clustering in the 17 species used for comparative analyses.

Supplementary Table S8. Statistics on detoxification, heatshock protein (HSP), and sensing-related genes across *Bactrocera* insects and other insects.

Supplementary Table S9. GC-MS Analysis of VOCs in *Maoping Tangerine*.

Supplementary Table S10. Chemical compounds used in this study.

Supplementary Table S11. Binding affinities of all tested ligands to BtsuOBPs.

Supplementary Table S12. BtsuOrs with ligands interaction energy calculated by molecular docking.

Data Availability

The genome sequence had been deposited at the National Center for Biotechnology Information (NCBI), under the accession number of JBKBCS000000000. The NCBI BioProject accession number is PRJNA1182520. The transcriptome sequence had been deposited at the National Center for

Biotechnology Information (NCBI), under the BioProject accession number is PRJNA1246368.

Abbreviations

1-NPN: N-phenyl-1-naphthylamine; ABC: ATP-binding cassette transporter; BLAST: Basic Local Alignment Search Tool; BUSCO: Benchmarking Universal Single-Copy Orthologs; CCE: Carboxyl/cholinesterase; CCS: Circular consensus sequence; CDS: Coding sequence; COG: Clusters of Orthologous Genes; CSP: Chemosensory protein; CYP450: Cytochrome P450 monooxygenase; EI: Electron ionization; GC-MS: Gas chromatography coupled with mass spectrometry; GFF: General feature format; GO: Gene Ontology; GR: Gustatory receptor; GST: Glutathione S-transferase; Hi-C: High-resolution chromosome conformation capture; HMM: Hidden Markov model; HSP: Heat shock protein; HS-SPME: Headspace Solid Phase Micro-extraction; IPTG: Isopropyl-β-D-thiogalactopyranoside; IR: Ionotropic receptor; KEGG: Kyoto Encyclopedia of Genes and Genomes; LINE: Long interspersed nuclear element; LTR: Long terminal repeat; MD: Molecular dynamics; MMGBSA: Molecular mechanics generalized Born surface area; Mya: Million years ago; NCBI: National Center for Biotechnology Information; OBP: Odorant-binding protein; OD: Optical density; OR: Odorant receptor; ORco: Odorant receptor co-receptor; ORF: Open reading frames; PCR: Polymerase Chain Reaction; RMSD: Root-mean-square deviation; RMSF: Root-mean-square fluctuation; RNA-seq: RNA sequencing; SASA: Solvent-accessible surface area; SDS-PAGE: Sodium dodecyl sulfate-polyacrylamide gel electrophoresis; SignalP: Signal peptides; SNMP: Sensory neuron membrane protein; UGT: UDP-glucuronosyltransferase; UTR: Untranslated regions; VDWAALS: Van der Waals forces.

Competing Interests

The authors declare no competing interests.

Funding

This work is supported by National Natural Science Foundation of China (32202288) and National Key R&D Program of China (2022YFC2601500).

Authors' Contributions

Tengda Guo, Yujia Qin and Zhihong Li conceived the project; Tengda Guo, Wenzhao Yang and Yuan Zhang performed the experiments; Tengda Guo and Weisong Li performed the bioinformatic analyses; Tengda Guo, Yujia Qin and Zhihong Li evaluated the results; Tengda Guo wrote the manuscript. Yujia Qin and Zhihong Li improved and revised the manuscript. All authors read and approved of the final manuscript.

Acknowledgments

We thank the 2115 Talent Development Program of China Agricultural University.

References

[1] Mochizuki M, Arai T, Mishiro K, Okazaki Y, Higashiura Y. Control of the Japanese orange fly, *Bactrocera tsuneonis* (Diptera: Tephritidae), through several preharvest management practices: establishment of a phytosanitary measure for citrus fruits for export. Appl Entomol Zool 2024;59:317–29. https://doi.org/10.1007/s13355-024-00881-w.

[2] Opadith P, Iwamoto S, Narahara M, Okazaki Y, Higashiura Y, Otake J, et al. Development of microsatellite markers for the Japanese orange fly, *Bactrocera tsuneonis* (Diptera: Tephritidae). Appl Entomol Zool 2022;57:283–8. https://doi.org/10.1007/s13355-022-00783-9.

[3] Ono H, Ota S, Kanno S, Nomura Y, Narahara M, Okazaki Y. Detection of environmental DNA of the Japanese orange fly, *Bactrocera tsuneonis* (Diptera: Tephritidae), from immature mandarin orange fruits. Appl Entomol Zoolog 2025;60:45–51. https://doi.org/10.1007/s13355-024-00890-9.

[4] Zhang Y, Feng S, Zeng Y, Ning H, Liu L, Zhao Z, et al. The first complete mitochondrial genome of *Bactrocera tsuneonis* (Miyake) (Diptera: Tephritidae) by next-generation sequencing and its phylogenetic implications. Int J Biol Macromol 2018;118:1229–37. https://doi.org/10.1016/j.ijbiomac.2018.06.099.

[5] Wu GA, Terol J, Ibanez V, López-García A, Pérez-Román E, Borredá C, et al. Genomics of the origin and evolution of Citrus. Nature 2018;554:311–6. https://doi.org/10.1038/nature25447.

[6] Li F, Zhao X, Li M, He K, Huang C, Zhou Y, et al. Insect genomes: progress and challenges. Insect Mol Biol 2019;28:739–58. https://doi.org/10.1111/imb.12599.

[7] Li F, Wang X, Zhou X. The Genomics Revolution Drives a New Era in Entomology. Annu Rev Entomol 2025;70:379–400. https://doi.org/10.1146/annurev-ento-013024-013420.

[8] Vargas RI, Pinero JC, Leblanc L. An Overview of Pest Species of *Bactrocera* Fruit Flies (Diptera: Tephritidae) and the Integration of Biopesticides with Other Biological Approaches for Their Management with a Focus on the Pacific Region. Insects 2015;6:297–318. https://doi.org/10.3390/insects6020297.

[9] Wu Z, Cui Y, Ma J, Qu M, Lin J. Analyses of chemosensory genes provide insight into the evolution of behavioral differences to phytochemicals in *Bactrocera* species. Mol Phylogenet Evol 2020;151:106858. https://doi.org/10.1016/j.ympev.2020.106858.

[10] Zhao Z, Carey JR, Li Z. The Global Epidemic of Bactrocera Pests: Mixed-Species Invasions and Risk Assessment. Annu Rev Entomol 2024;69:219–37. https://doi.org/10.1146/annurev-ento-012723-102658.

[11] Liu Z, Xie Q, Guo H, Xu W, Wang J. An odorant binding protein mediates *Bactrocera dorsalis*

olfactory sensitivity to host plant volatiles and male attractant compounds. Int J Biol Macromol 2022;219:538–44. https://doi.org/10.1016/j.ijbiomac.2022.07.198.

[12] Zhang Y, Liu W, Luo Z, Yuan J, Wuyun Q, Zhang P, et al. Odorant Receptor BdorOR49b Mediates Oviposition and Attraction Behavior of *Bactrocera dorsalis* to Benzothiazole. J Agric Food Chem 2024;72:7784–93. https://doi.org/10.1021/acs.jafc.3c09791.

[13] Vogt RG, Rogers ME, Franco MD, Sun M. A comparative study of odorant binding protein genes:: differential expression of the PBP1-GOBP2 gene cluster in *Manduca sexta* (Lepidoptera) and the organization of OBP genes in *Drosophila melanogaster* (Diptera). J Exp Biol 2002;205:719–44. https://doi.org/10.1242/jeb.205.6.719.

[14] Pelosi P, Maida R. Odorant-Binding Proteins in Insects. Comp Biochem Physiol B-Biochem Molec Biol 1995;111:503–14. https://doi.org/10.1016/0305-0491(95)00019-5.

[15] Sandler BH, Nikonova L, Leal WS, Clardy J. Sexual attraction in the silkworm moth: structure of the pheromone-binding-protein-bombykol complex. Chem Biol 2000;7:143–51. https://doi.org/10.1016/S1074-5521(00)00078-8.

[16] Leal WS, Odorant Reception in Insects: Roles of Receptors, Binding Proteins, and Degrading Enzymes. Annu Rev Entomol 2013;58:373–91. https://doi.org/10.1146/annurev-ento-120811-153635.

[17] Pelosi P, Zhou J, Ban LP, Calvello M. Soluble proteins in insect chemical communication. Cell Mol Life Sci 2006;63:1658–76. https://doi.org/10.1007/s00018-005-5607-0.

[18] Zhou J, Robertson G, He X, Dufour S, Hooper AM, Pickett JA, et al. Characterisation of Bombyx mori Odorant-binding Proteins Reveals that a General Odorant-binding Protein Discriminates Between Sex Pheromone Components. J Mol Biol 2009;389:529–45. https://doi.org/10.1016/j.jmb.2009.04.015.

[19] Brito NF, Moreira MF, Melo ACA. A look inside odorant-binding proteins in insect chemoreception. J Insect Physiol 2016;95:51–65. https://doi.org/10.1016/j.jinsphys.2016.09.008.

[20] Sachse S, Krieger J. Olfaction in insects. e-Neuroforum 2011;2:49–60. https://doi.org/10.1007/s13295-011-0020-7

[21] Wicher D, Miazzi F. Functional properties of insect olfactory receptors: ionotropic receptors and odorant receptors. Cell Tissue Res 2021;383:7–19. https://doi.org/10.1007/s00441-020-03363-x.

[22] Vosshall LB, Hansson BS. A Unified Nomenclature System for the Insect Olfactory Coreceptor. Chem Senses 2011;36:497–8. https://doi.org/10.1093/chemse/bjr022.

[23] Ha TS, Smith DP. Odorant and pheromone receptors in insects. Front Cell Neurosci 2009;3:10.

https://doi.org/10.3389/neuro.03.010.2009.

[24] Reed RR. After the holy grail: Establishing a molecular basis for mammalian olfaction. Cell 2004;116:329–36. https://doi.org/10.1016/S0092-8674(04)00047-9.

[25] Butterwick JA, del Marmol J, Kim KH, Kahlson MA, Rogow JA, Walz T, et al. Cryo-EM structure of the insect olfactory receptor Orco. Nature 2018;560:447–52. https://doi.org/10.1038/s41586-018-0420-8.

[26] del Marmol J, Yedlin MA, Ruta V. The structural basis of odorant recognition in insect olfactory receptors. Nature 2021;597:126–31. https://doi.org/10.1038/s41586-021-03794-8.

[27] Wang Y, Qiu L, Wang B, Guan Z, Dong Z, Zhang J, et al. Structural basis for odorant recognition of the insect odorant receptor OR-Orco heterocomplex. Science 2024;384:1453–60. https://doi.org/10.1126/science.adn6881.

[28] Zheng L, Zhang Y, Yang W, Zeng Y, Jiang F, Qin Y, et al. New Species-Specific Primers for Molecular Diagnosis of *Bactrocera minax* and *Bactrocera tsuneonis* (Diptera: Tephritidae) in China Based on DNA Barcodes. Insects 2019;10:447. https://doi.org/10.3390/insects10120447.

[29] Marcais G, Kingsford C. A fast, lock-free approach for efficient parallel counting of occurrences of k-mers. Bioinformatics 2011;27:764–70. https://doi.org/10.1093/bioinformatics/btr011.

[30] Vurture GW, Sedlazeck FJ, Nattestad M, Underwood CJ, Fang H, Gurtowski J, et al. GenomeScope: fast reference-free genome profiling from short reads. Bioinformatics 2017;33:2202–4. https://doi.org/10.1093/bioinformatics/btx153.

[31] Cheng H, Concepcion GT, Feng X, Zhang H, Li H. Haplotype-resolved de novo assembly using phased assembly graphs with hifiasm. Nat Methods 2021;18:170–5. https://doi.org/10.1038/s41592-020-01056-5.

[32] Guan D, McCarthy SA, Wood J, Howe K, Wang Y, Durbin R. Identifying and removing haplotypic duplication in primary genome assemblies. Bioinformatics 2020;36:2896–8. https://doi.org/10.1093/bioinformatics/btaa025.

[33] Li H, Durbin R. Fast and accurate short read alignment with Burrows-Wheeler transform. Bioinformatics 2009;25:1754–60. https://doi.org/10.1093/bioinformatics/btp324.

[34] Dudchenko O, Batra SS, Omer AD, et al. De novo assembly of the *Aedes aegypti* genome using Hi-C yields chromosome-length scaffolds. Science 2017;356:92–5. https://doi.org/10.1126/science.aal3327.

[35] Durand NC, Shamim MS, Machol I, et al. Juicer provides a oneclick system for analyzing loop-resolution Hi-C experiments. Cell Syst 2016;3:95–8. https://doi.org/10.1016/j.cels.2016.07.002.

[36] Chen N. Using RepeatMasker to identify repetitive elements in genomic sequences. Curr Protoc Bioinformatics 2004;5:4–10.https://doi.org/10.1002/0471250953.bi0410s25.

[37] Storer J, Hubley R, Rosen J, Wheeler TJ, Smit AF. The Dfam community resource of transposable element families, sequence models, and genome annotations. Mob DNA 2021;12:2. https://doi.org/10.1186/s13100-020-00230-y.

[38] Jurka J, Kapitonov VV, Pavlicek A, et al. Repbase Update, a database of eukaryotic repetitive elements. Cytogenet Genome Res 2005;110:462–7. https://doi.org/10.1159/000084979.

[39] Flynn JM, Hubley R, Goubert C, et al. RepeatModeler2 for automated genomic discovery of transposable element families. Proc Natl Acad Sci USA 2020;117:9451–7. https://doi.org/10.1073/pnas.1921046117.

[40] Ou S, Jiang N. LTR_FINDER_parallel: parallelization of LTR_FINDER enabling rapid identification of long terminal repeat retrotransposons. Mobile DNA 2019;10:48. https://doi.org/10.1186/s13100-019-0193-0.

[41] Ou S, Jiang N. LTR_retriever: a highly accurate and sensitive program for identification of long terminal repeat retrotransposons. Plant Physiol 2018;176:1410–22. https://doi.org/10.1104/pp.17.01310.

[42] Benson G. Tandem repeats finder: a program to analyze DNA sequences. Nucleic Acids Res 1999;27:573–80. https://doi.org/10.1093/nar/27.2.573.

[43] Stanke M, Keller O Gunduz I Hayes A Waack S Morgenstern B. AUGUSTUS: ab initio prediction of alternative transcripts. Nucleic Acids Res 2006;34: 435–9. https://doi.org/10.1093/nar/gkl200.

[44] Majoros WH, Pertea M, Salzberg SL. TigrScan and GlimmerHMM:: two open source ab initio eukaryotic gene-finders. Bioinformatics 2004;20:2878–9. https://doi.org/10.1093/bioinformatics/bth315.

[45] Keilwagen J, Wenk M, Erickson JL, Schattat MH, Grau J, Hartung F. Using intron position conservation for homology-based gene prediction. Nucleic Acids Res 2016;44:e89. https://doi.org/10.1093/nar/gkw092.

[46] Haas BJ, Salzberg SL, Zhu W, Pertea M, Allen JE, Orvis J, et al. Automated eukaryotic gene structure annotation using EVidenceModeler and the program to assemble spliced alignments. Genome Biol 2008;9:R7. https://doi.org/10.1186/gb-2008-9-1-r7.

[47] Buchfink B, Xie C, Huson DH. Fast and sensitive protein alignment using DIAMOND. Nat Methods 2015;12:59–60. https://doi.org/10.1038/nmeth.3176.

[48] Jones P, Binns D, Chang H-Y, Fraser M, Li W, McAnulla C, et al. InterProScan 5: genome-scale protein function classification. Bioinformatics 2014;30:1236–40. https://doi.org/10.1093/bioinformatics/btu031.

[49] Huerta-Cepas J, Forslund K, Coelho LP, Szklarczyk D, Jensen LJ, von Mering C, et al. Fast

Genome-Wide Functional Annotation through Orthology Assignment by eggNOG-Mapper. Mol Biol Evol 2017;34:2115–22. https://doi.org/10.1093/molbev/msx148.
[50] Chen C, Wu Y, Li J, Wang X, Zeng Z, Xu J, et al. TBtools-II: A "one for all, all for one"bioinformatics platform for biological big-data mining. Mol Plant 2023;16:1733–42. https://doi.org/10.1016/j.molp.2023.09.010.
[51] Emms DM, Kelly S. OrthoFinder: solving fundamental biases in whole genome comparisons dramatically improves orthogroup inference accuracy. Genome Biol 2015;16:157. https://doi.org/10.1186/s13059-015-0721-2.
[52] Laetsch DR, Blaxter ML. KinFin: Software for Taxon-Aware Analysis of Clustered Protein Sequences. G3-Genes Genomes Genet 2017;7:3349–57. https://doi.org/10.1534/g3.117.300233.
[53] Capella-Gutierrez S, Silla-Martinez JM, Gabaldon T. trimAl: atool for automated alignment trimming in large-scale phylogenetic analyses. Bioinformatics 2009;25:1972–3. https://doi.org/10.1093/bioinformatics/btp348.
[54] Stamatakis A. RAxML version 8: a tool for phylogenetic analysis and post-analysis of large phylogenies. Bioinformatics 2014;30:1312–3. https://doi.org/10.1093/bioinformatics/btu033.
[55] Yang Z. PAML 4: phylogenetic analysis by maximum likelihood. Mol Biol Evol 2007;24:1586–91. https://doi.org/10.1093/molbev/msm088.
[56] Kumar S, Suleski M, Craig JM, Kasprowicz AE, Sanderford M, Li M, et al. TimeTree 5: An Expanded Resource for Species Divergence Times. Mol Biol Evol 2022;39:msac174. https://doi.org/10.1093/molbev/msac174.
[57] Krosch MN, Schutze MK, Armstrong KF, Graham GC, Yeates DK, Clarke AR. A molecular phylogeny for the Tribe Dacini (Diptera: Tephritidae): Systematic and biogeographic implications. Mol Phylogenet Evol 2012;64:513–23. https://doi.org/10.1016/j.ympev.2012.05.006.
[58] Yaakop S, Ibrahim NJ, Shariff S, Zain BMM. Molecular clock analysis on five *Bactrocera* species flies (Diptera: Tephritidae) based on combination of COI and NADH sequences. Orient Insects 2015;49:150–64. https://doi.org/10.1080/00305316.2015.1081421.
[59] Zhao Z, Su T, Chesters D, Wang S, Ho SYW, Zhu C, et al. The Mitochondrial Genome of *Elodia flavipalpis* Aldrich (Diptera: Tachinidae) and the Evolutionary Timescale of Tachinid Flies. PLoS One 2013;8:e61814. https://doi.org/10.1371/journal.pone.0061814.
[60] Russo CAM, Mello B, Frazao A, Voloch CM. Phylogenetic analysis and a time tree for a large drosophilid data set (Diptera: Drosophilidae). Zool J Linn Soc 2013;169:765–75. https://doi.org/10.1111/zoj12062.
[61] Gaunt MW, Miles MA. An insect molecular clock dates the origin of the insects and accords

with palaeontological and biogeographic landmarks. Mol Biol Evol 2002;19:748–61. https://doi.org/10.1093/oxfordjournals.molbev.a004133.

[62] Nardi F, Carapelli A, Boore JL, Roderick GK, Dallai R, Frati F. Domestication of olive fly through a multi-regional host shift to cultivated olives: Comparative dating using complete mitochondrial genomes. Mol Phylogenet Evol 2010;57:678–86. https://doi.org/10.1016/j.ympev.2010.08.008.

[63] Xie J, Chen Y, Cai G, Cai R, Hu Z, Wang H. Tree Visualization By One Table (tvBOT): a web application for visualizing, modifying and annotating phylogenetic trees. Nucleic Acids Res 2023;51:W587–92. https://doi.org/10.1093/nar/gkad359.

[64] De Bie T, Cristianini N, Demuth JP, et al. CAFE: a computational tool for the study of gene family evolution. Bioinformatics 2006;22:1269–71. https://doi.org/10.1093/bioinformatics/btl097.

[65] Finn RD, Bateman A, Clements J, Coggill P, Eberhardt RY, Eddy SR, et al. Pfam: the protein families database. Nucleic Acids Res 2014;42:222–30. https://doi.org/10.1093/nar/gkt1223.

[66] McGinnis S, Madden TL. BLAST: at the core of a powerful and diverse set of sequence analysis tools. Nucleic Acids Res 2004;32:20–5. https://doi.org/10.1093/nar/gkh435.

[67] Potter SC, Luciani A, Eddy SR, et al. HMMER web server: 2018 update. Nucleic Acids Res 2018;46:W200–4. https://doi.org/10.1093/nar/gky448.

[68] Vizueta J, Sanchez-Gracia A, Rozas J. bitacora: A comprehensive tool for the identification and annotation of gene families in genome assemblies. Mol Ecol Resour 2020;20:1445–52. https://doi.org/10.1111/1755-0998.13202.

[69] Edgar RC. MUSCLE: multiple sequence alignment with high accuracy and high throughput. Nucleic Acids Res 2004;32:1792–7. https://doi.org/10.1093/nar/gkh340.

[70] Minh BQ, Schmidt HA, Chernomor O, et al. IQ-TREE 2: new models and efficient methods for phylogenetic inference in the genomic era. Mol Biol Evol 2020;37:1530–4. https://doi.org/10.1093/molbev/msaa015.

[71] Kim D, Paggi JM, Park C, Bennett C, Salzberg SL. Graph-based genome alignment and genotyping with HISAT2 and HISAT-genotype. Nat Biotechnol 2019;37:907–15. https://doi.org/10.1038/s41587-019-0201-4.

[72] Liao Y, Smyth GK, Shi W. The R package Rsubread is easier, faster, cheaper and better for alignment and quantification of RNA sequencing reads. Nucleic Acids Res 2019;47:e47. https://doi.org/10.1093/nar/gkz114.

[73] Mu H, Chen J, Huang W, Huang G, Deng M, Hong S, et al. OmicShare tools: A zero-code interactive online platform for biological data analysis and visualization. iMeta 2024;3.

https://doi.org/10.1002/imt2.228.
[74] Romeo JT. New SPME guidelines. J Chem Ecol 2009;35:1383. https://doi.org/10.1007/s10886-009-9733-2.
[75] Bradford M. Rapid and Sensitive Method for Quantitation of Microgram Quantities of Protein Utilizing Principle of Protein-Dye Binding. Anal Biochem 1976;72:248–54. https://doi.org/10.1016/0003-2697(76)90527-3.
[76] Jumper J, Evans R, Pritzel A, Green T, Figurnov M, Ronneberger O, et al. Highly accurate protein structure prediction with AlphaFold. Nature 2021;596:583–9. https://doi.org/10.1038/s41586-021-03819-2.
[77] Graef J, Ehrt C, Rarey M. Binding Site Detection Remastered: Enabling Fast, Robust, and Reliable Binding Site Detection and Descriptor Calculation with DoGSite3. J Chem Inf Model 2023;63:1–10. https://doi.org/10.1021/acs.jcim.3c00336.
[78] Volkamer A, Griewel A, Grombacher T, Rarey M. Analyzing the Topology of Active Sites: On the Prediction of Pockets and Subpockets. J Chem Inf Model 2010;50:2041–52. https://doi.org/10.1021/ci100241y.
[79] Volkamer A, Kuhn D, Grombacher T, Rippmann F, Rarey M. Combining Global and Local Measures for Structure-Based Druggability Predictions. J Chem Inf Model 2012;52:360–72. https://doi.org/10.1021/ci200454v.
[80] Trott O, Olson AJ. Software News and Update AutoDock Vina: Improving the Speed and Accuracy of Docking with a New Scoring Function, Efficient Optimization, and Multithreading. J Comput Chem 2010;31:455–61. https://doi.org/10.1002/jcc.21334.
[81] Van der Spoel D, Lindahl E, Hess B, Groenhof G, Mark AE, Berendsen HJC. GROMACS: Fast, flexible, and free. J Comput Chem 2005;26:1701–18. https://doi.org/10.1002/jcc.20291.
[82] Abraham MJ, Murtola T, Schulz R, Páll S, Smith JC, Hess B, et al. GROMACS: High performance molecular simulations through multi-level parallelism from laptops to supercomputers. SoftwareX 2015;1–2:19–25. https://doi.org/10.1016/j.softx.2015.06.001.
[83] Li S, Zhu S, Jia Q, Yuan D, Ren C, Li K, et al. The genomic and functional landscapes of developmental plasticity in the American cockroach. Nat Commun 2018;9:1008. https://doi.org/10.1038/s41467-018-03281-1.
[84] Francis F, Vanhaelen N, Haubruge E. Glutathione S-transferases in the adaptation to plant secondary metabolites in the *Myzus persicae* aphid. Arch Insect Biochem Physiol 2005;58:166–74. https://doi.org/10.1002/arch.20049.
[85] Jin R, Mao K, Liao X, Xu P, Li Z, Ali E, et al. Overexpression of *CYP6ER1* associated with clothianidin resistance in *Nilaparvata lugens* (Stal). Pest Biochem Physiol 2019;154:39–45.

https://doi.org/10.1016/j.pestbp.2018.12.008.

[86] Feder ME, Hofmann GE. Heat-shock proteins, molecular chaperones, and the stress response: Evolutionary and ecological physiology. Annu Rev Physiol 1999;61:243–82. https://doi.org/10.1146/annurev.physiol.61.1.243.

[87] García-Reina A, Rodríguez-García MJ, Ramis G, et al. Real-time cell analysis and heat shock protein gene expression in the TcA *Tribolium castaneum* cell line in response to environmental stress conditions: RTCA and hsps expression in the TcA cell line. Insect Sci 2017;24:358–70. https://doi.org/10.1111/1744-7917.12306.

[88] Lu K, Chen X, Liu W, et al. Characterization of heat shock protein 70 transcript from *Nilaparvata lugens* (Stål): its response to temperature and insecticide stresses. Pestic Biochem Physiol 2017;142:102–10. https://doi.org/10.1016/j.pestbp.2017.01.011.

[89] Eyun S, Soh HY, Posavi M, Munro JB, Hughes DST, Murali SC, et al. Evolutionary History of Chemosensory-Related Gene Families across the Arthropoda. Molecular Biology and Evolution 2017;34:1838–62. https://doi.org/10.1093/molbev/msx147.

[90] Robertson HM. Molecular Evolution of the Major Arthropod Chemoreceptor Gene Families. Annual Review of Entomology 2019;64:227–42. https://doi.org/10.1146/annurev-ento-020117-043322.

[91] Vogt RG, Miller NE, Litvack R, Fandino RA, Sparks J, Staples J, et al. The insect SNMP gene family. Insect Biochem Mol Biol 2009;39:448–56. https://doi.org/10.1016/j.ibmb.2009.03.007.

[92] Xu PX, Atkinson R, Jones DNM, Smith DP. *Drosophila* OBP LUSH is required for activity of pheromone-sensitive neurons. Neuron 2005;45:193–200. https://doi.org/10.1016/j.neuron.2004.12.031.

[93] Sessegolo C, Burlet N, Haudry A. Strong phylogenetic inertia on genome size and transposable element content among 26 species of flies. Biol Lett 2016;12:20160407. https://doi.org/10.1098/rsbl.2016.0407.

[94] Zhao L, Yuan H, Liu X, Chang H, Jing X, Nie Y, et al. Evolutionary dynamics of repetitive elements and their relationship with genome size in Acrididae. Genomics 2025;117:110971. https://doi.org/10.1016/j.ygeno.2024.110971.

[95] Cabral-de-Mello DC, Palacios-Gimenez OM. Repetitive DNAs: the “invisible” regulators of insect adaptation and speciation. Curr Opin Insect Sci 2025;67:101295. https://doi.org/10.1016/j.cois.2024.101295.

[96] Jiang F, Liang L, Wang J, Zhu S. Chromosome-level genome assembly of *Bactrocera dorsalis* reveals its adaptation and invasion mechanisms. Commun Biol 2022;5:25. https://doi.org/10.1038/s42003-021-02966-6.

[97] Guo T, Feng S, Zhang Y, Li W, Qin Y, Li Z. Chromosome-level genome assembly of *Bactrocera correcta* provides insights into its adaptation and invasion mechanisms. Genomics 2023;115:110736. https://doi.org/10.1016/j.ygeno.2023.110736.
[98] He X, Tzotzos G, Woodcock C, Pickett JA, Hooper T, Field LM, et al. Binding of the General Odorant Binding Protein of Bombyx mori BmorGOBP2 to the Moth Sex Pheromone Components. J Chem Ecol 2010;36:1293–305. https://doi.org/10.1007/s10886-010-9870-7.
[99] Liu Y, Gu S, Zhang Y, Guo Y, Wang G. Candidate Olfaction Genes Identified within the Helicoverpa armigera Antennal Transcriptome. PLoS One 2012;7:e48260. https://doi.org/10.1371/journal.pone.0048260.
[100] Wang Y, Fang G, Xu P, Gao B, Liu X, Qi X, et al. Behavioral and genomic divergence between a generalist and a specialist fly. Cell Reports 2022;41:111654. https://doi.org/10.1016/j.celrep.2022.111654.
[101] Fonseca PM, Robe LJ, Carvalho TL, Loreto ELS. Characterization of the chemoreceptor repertoire of a highly specialized fly with comparisons to other Drosophila species. Genet Mol Biol 2024;47:e20220383. https://doi.org/10.1590/1678-4685-GMB-2022-0383.
[102] Nauen R, Bass C, Feyereisen R, et al. The role of cytochrome P450s in insect toxicology and resistance. Annu Rev Entomol 2022;67:105–24. https://doi.org/10.1146/annurev-ento-070621-061328.
[103] Duan S, Mao L, Sun S, Chen R, Abdelkhalek ST, Wang M. Key site residues of *Cnaphalocrocis medinalis* odorant-binding protein 13 CmedOBP13 involved in interacting with rice plant volatiles. Int J Biol Macromol 2025;290:139007. https://doi.org/10.1016/j.ijbiomac.2024.139007.
[104] Yang Y, Tan S, Wang Q, Wang F, Zhang Y. Key amino acids in odorant-binding protein OBP7 enable *Bradysia odoriphaga* to recognize host plant volatiles. Int J Biol Macromol 2025;284:138179. https://doi.org/10.1016/j.ijbiomac.2024.138179.
[105] Chen X, Lei Y, Liang C, Lei Q, Wang J, Jiang H. Odorant Binding Protein Expressed in Legs Enhances Malathion Tolerance in *Bactrocera dorsalis* (Hendel). J Agric Food Chem 2024;72:4376–83. https://doi.org/10.1021/acs.jafc.3c08458.
[106] Vosshall LB, Amrein H, Morozov PS, Rzhetsky A, Axel R. A spatial map of olfactory receptor expression in the *Drosophila* antenna. Cell 1999;96:725–36. https://doi.org/10.1016/S0092-8674(00)80582-6.
[107] Miyazaki H, Otake J, Mitsuno H, Ozaki K, Kanzaki R, Chieng AC-T, et al. Functional characterization of olfactory receptors in the Oriental fruit fly *Bactrocera dorsalis* that respond to plant volatiles. Insect Biochem Mol Biol 2018;101:32–46.

https://doi.org/10.1016/j.ibmb.2018.07.002.

[108] Xu L, Jiang H-B, Yu J-L, Lei Q, Pan D, Chen Y, et al. An Odorant Receptor Expressed in Both Antennae and Ovipositors Regulates Benzothiazole-Induced Oviposition Behavior in *Bactrocera dorsalis*. J Agric Food Chem 2024;72:6954–63. https://doi.org/10.1021/acs.jafc.3c09557.

[109] Wang C, Cao S, Shi C, Guo M, Sun D, Liu Z, et al. The novel function of an orphan pheromone receptor reveals the sensory specializations of two potential distinct types of sex pheromones in noctuid moth. Cell Mol Life Sci 2024;81:259. https://doi.org/10.1007/s00018-024-05303-2.

[110] Lin C-C, Prokop-Prigge KA, Preti G, Potter CJ. Food odors trigger *Drosophila* males to deposit a pheromone that guides aggregation and female oviposition decisions. Elife 2015;4. https://doi.org/10.7554/eLife.08688.

[111] Miyazaki H, Otake J, Mitsuno H, Ozaki K, Kanzaki R, Chieng AC-T, et al. Functional characterization of olfactory receptors in the Oriental fruit fly *Bactrocera dorsalis* that respond to plant volatiles. Insect Biochem Mol Biol 2018;101:32–46. https://doi.org/10.1016/j.ibmb.2018.07.002.

[112] Ono H. Functional characterization of an olfactory receptor in the Oriental fruit fly, *Bactrocera dorsalis*, that responds to eugenol and isoeugenol. Comp Biochem Physiol B-Biochem Mol Biol 2022;258:110696. https://doi.org/10.1016/j.cbpb.2021.110696.

[113] Fleischer J, Pregitzer P, Breer H, Krieger J. Access to the odor world: olfactory receptors and their role for signal transduction in insects. Cell Mol Life Sci 2018;75:485–508. https://doi.org/10.1007/s00018-017-2627-5.

[114] Xue J, Zhou X, Zhang C, Yu L, Fan H, Wang Z, et al. Genomes of the rice pest brown planthopper and its endosymbionts reveal complex complementary contributions for host adaptation. Genome Biol 2014;15:521. https://doi.org/10.1186/s13059-014-0521-0.

[115] Steffan-Dewenter I, Tscharntke T. Butterfly community structure in fragmented habitats. Ecol Lett 2000;3:449–56. https://doi.org/10.1111/j.1461-0248.2000.00175.x.

[116] Hafsi A, Facon B, Ravigné V, Chiroleu F, Quilici S, Chermiti B, et al. Host plant range of a fruit fly community (Diptera: Tephritidae): does fruit composition influence larval performance? BMC Ecology 2016;16:40. https://doi.org/10.1186/s12898-016-0094-8.

[117] Facon B, Hafsi A, Charlery de la Masselière M, Robin S, Massol F, Dubart M, et al. Joint species distributions reveal the combined effects of host plants, abiotic factors and species competition as drivers of species abundances in fruit flies. Ecology Letters 2021;24:1905–16. https://doi.org/10.1111/ele.13825.

[118] Charlery de la Masselière M, Facon B, Hafsi A, Duyck P-F. Diet breadth modulates preference

- performance relationships in a phytophagous insect community. Sci Rep 2017;7:16934. https://doi.org/10.1038/s41598-017-17231-2.
