# Supplementary material for: A high-quality chromosome-level genome assembly of the oligophagous fruit fly Bactrocera tsuneonis (Diptera: Tephritidae) and insights into its host specificity
Source: Gigascience. 2025 Nov 20;14:giaf143. doi: 10.1093/gigascience/giaf143 (PMC12723664; doi:10.1093/gigascience/giaf143)
Supplement: giaf143_Authors_Response_To_Reviewer_Comments_Original_Submission [file giaf143_authors_response_to_reviewer_comments_original_submission.pdf]

Author's Response To Reviewer Comments

Revised version for “A high-quality chromosome-level genome assembly of the oligophagous fruit fly Bactrocera tsuneonis (Diptera: Tephritidae) and insights into its host specificity”

Dear editor,

We are honored to have the opportunity to resubmit our manuscript entitled “A high-quality chromosome-level genome assembly of the oligophagous fruit fly Bactrocera tsuneonis (Diptera: Tephritidae) and insights into its host specificity”. We would like to express our sincere gratitude to you and the reviewers for your valuable time and insightful comments, which have significantly contributed to the improvement of our manuscript. We have carefully addressed all the comments and revised the manuscript accordingly.

We hereby confirm that the manuscript has not been published, accepted for publication, or is under consideration for publication elsewhere, either in whole or in part. This work is original, and all necessary acknowledgements have been made. The submission has been approved by all authors and affiliated institutions, and all authors have read and agreed to the final version of the manuscript. Please feel free to contact us if any further information is needed.
Yours sincerely,
Corresponding Author: Yujia Qin qinyujia@cau.edu.cn; Zhihong Li lizh@cau.edu.cn
First Author: Tengda Guo guotd7911@163.com

Reviewer #1
Comment 1: Identification of specific active volatile compounds attracting B. tsuneonis to citrus, particularly through Y-tube olfactometer assays to confirm the attractiveness of trans-nerolidol and piperitone.
Authors’ reply: We appreciate this valuable suggestion. At present, due to the lack of a stable laboratory colony for B. tsuneonis and the difficulty of maintaining adults under controlled conditions, we were unable to conduct Y-tube olfactometer assays. However, we acknowledge the importance of behavioral validation and have discussed this limitation explicitly in the revised Discussion (line 675-678). We are actively working to overcome current challenges in laboratory rearing and aim to conduct behavioral assays in future studies to directly test the attractiveness of these volatiles.

Comment 2: While molecular docking and dynamics simulations suggest the potential of BtsuOr7a-6 and BtsuOr7a-4 to bind trans-nerolidol and piperitone, additional experimental validation is essential to confirm their role as receptors.
Authors’ reply: We thank for this suggestion. While heterologous expression systems (e.g., Xenopus oocytes or transgenic Drosophila) are ideal, these require considerable setup. We have now included this as a perspective in the revised manuscript (line 704-706), and we are actively planning further OR functional assays via heterologous systems in future work.

Comment 3: Transcriptomic findings highlighting OBP and OR genes with heightened expression in the antennae necessitate validation through quantitative PCR.
Authors’ reply: We have conducted qRT-PCR for a set of experiment related OBP and OR genes that showed antennae-biased expression in the transcriptomic data. The results support the RNA-seq patterns and are now provided in the revised Results section (line 489-494) and Methods (line 241-246).

Reviewer #2
Comment 1: The insects were collected from the wild. It is not indicated if one specimen was used for DNA extraction, or if more than one was used to extract DNA and DNA sequencing data. Also the sex of the specimen(s) was not indicated.
Authors’ reply: Has been revised, line 117-123, “For Illumina sequencing, genomic DNA was extracted from a single male adult (with the abdomen removed) using the Wizard SV Genomic DNA Purification System Kit from Promega, and the quality and concentration of the DNA were measured using a microvolume UV

spectrophotometer” and “For long-read sequencing, genomic DNA was extracted from a single male adult (with the abdomen removed)”.

Comment 2: The same applies to the RNA and RNA-seq data, how many specimens were used for collecting tissues, what was the sex?
Authors’ reply: Has been revised, line 136-139, “For transcriptome sequencing, different body parts, including 50 antennae,5 heads (without antennae),10 legs, and 5 ovipositors (female only), were dissected separately from field-collected female and male adults. Three biological replicates were prepared for each tissue type. Total RNA was extracted using standard protocols”.

Comment 3: Figure 1: It is not clear as to whether chromosome y has no homology to any other chromosome of the species compared.
Authors’ reply: As noted in the Results section (line 378-382), “no syntenic genes associated with the D. melanogaster Y chromosome were identified in the six assembled chromosomes or unanchored scaffolds of B. tsuneonis,” indicating that no Y-linked homology could be detected in our assembly. The figure legend for Figure 1 already reflects this, and we respectfully believe no further modification is needed.

Comment 4: A comparative analysis of the tephritidae sex chromosomes is not presented, in order to compare synteny levels to the one of autosomes.
Authors’ reply: In the Results section (line 378-382), B. tsuneonis Chr02 and B. dorsalis Chr04 exhibit collinearity with the X chromosome of D. melanogaster, suggesting they may represent putative sex chromosomes. In contrast, no syntenic genes associated with the Y chromosome were detected. These comparisons provide preliminary insights into the structure and evolution of Tephritid sex chromosomes relative to autosomes, which we plan to investigate further in future work.

Editorial Manager®

Cookie Preference Center

We use cookies which are necessary to make our site work. We may also use additional cookies to analyze, improve and personalize our content and your digital experience. For more information, see our Cookie Policy.

You may choose not to allow some types of cookies. However, blocking some types may impact your experience of our site and the services we are able to offer. See the different category headings below to find out more or change your settings.

Allow all

Manage Consent Preferences

Strictly Necessary Cookies

Always active

These cookies are necessary for the website to function and cannot be switched off in our systems. They are usually only set in response to actions made by you which amount to a request for services, such as setting your privacy preferences, logging in or filling in forms. You can set your browser to block or alert you about these cookies, but some parts of the site will not then work.

Functional Cookies

These cookies enable the website to provide enhanced functionality and personalisation. They may be set by us or by third party providers whose services we have added to our pages. If you do not allow these cookies then some or all of these services may not function properly.

Performance Cookies

These cookies allow us to count visits and traffic sources so we can measure and improve the performance of our site. They help us to know which pages are the most and least popular and see how visitors move around the site.

Targeting Cookies

These cookies may be set through our site by our advertising partners. They may be used by those companies to build a profile of your interests and show you relevant adverts on other sites. If you do not allow these cookies, you will experience less targeted advertising.

Cookie List

Clear

Apply Cancel

Consent Leg.Interest

Confirm my choices

Powered by onetrust
