# Supplementary material for: A high-quality chromosome-level genome assembly of the oligophagous fruit fly Bactrocera tsuneonis (Diptera: Tephritidae) and insights into its host specificity
Source: Gigascience. 2025 Nov 20;14:giaf143. doi: 10.1093/gigascience/giaf143 (PMC12723664; doi:10.1093/gigascience/giaf143)
Supplement: giaf143_Authors_Response_To_Reviewer_Comments_Revision_1 [file giaf143_authors_response_to_reviewer_comments_revision_1.pdf]

Author's Response To Reviewer Comments

Revised version for “A high-quality chromosome-level genome assembly of the oligophagous fruit fly Bactrocera tsuneonis (Diptera: Tephritidae) and insights into its host specificity”

Dear editor,

We are honored to have the opportunity to resubmit our manuscript entitled “A high-quality chromosome-level genome assembly of the oligophagous fruit fly Bactrocera tsuneonis (Diptera: Tephritidae) and insights into its host specificity”. We would like to express our sincere gratitude to you and the reviewers for your valuable time and insightful comments, which have significantly contributed to the improvement of our manuscript. We have carefully addressed all the comments and revised the manuscript accordingly.

We hereby confirm that the manuscript has not been published, accepted for publication, or is under consideration for publication elsewhere, either in whole or in part. This work is original, and all necessary acknowledgements have been made. The submission has been approved by all authors and affiliated institutions, and all authors have read and agreed to the final version of the manuscript. Please feel free to contact us if any further information is needed.
Yours sincerely,
Corresponding Author: Yujia Qin qinyujia@cau.edu.cn; Zhihong Li lizh@cau.edu.cn
First Author: Tengda Guo guotd7911@163.com

Reviewer #3
Comment 1: This assembly is really PacBio + HiC, the Illumina data was not used for assembly. Illumina data was used in kmer based analysis, but the HiFi data alone could also have been used for this same analysis.
Authors’ reply: We have revised the text to clarify data usage. Illumina short reads were used only for genome survey and quality evaluation, while the final chromosome-level assembly was generated solely from PacBio HiFi and Hi-C data. Line 102-104: “In this study, we assembled a high-quality chromosome-level genome of B. tsuneonis using a combination of PacBio high-fidelity (HiFi) sequencing, and high-resolution chromosome conformation capture (Hi-C) technologies”. Line 170-171: “The Illumina short reads were used solely for this purpose and were not included in the assembly process”. Line 379-380: “A total of 24.91 Gb of Illumina short reads, 37.82 Gb of PacBio HiFi long reads, and 58.88 Gb of Hi-C data were generated”.

Comment 2: in the methods, full length transcriptome sequencing was mentioned (essentially IsoSeq), but it was unclear how it was analyzed and how it was used for overall genome annotation, manual annotation of genes (particularly can be useful for chemosensory genes) or tissue specific analyses. How was this data used?
Authors’ reply: We have revised the Methods section to clarify the use of Iso-Seq data in genome annotation. Line 200-203: “Simultaneously, full-length transcriptomic (Iso-Seq) data were incorporated into the annotation workflow to support transcriptome-based gene prediction. The polished Iso-Seq isoforms were aligned to the assembled genome and used to enhance gene model accuracy”. Line 433-435: “Protein-coding genes in the B. tsuneonis genome were predicted using a combination of three approaches: ab initio prediction, homology-based prediction, and full-length transcriptome (Iso-Seq)-based prediction”.

Comment 3: I was able to follow, but really a better description of how plant chemistry is being used in combination of protein structure prediction, modeling, and protein expression needs to be made. Really walk people through how the structure of the insect proteins is being compared to the compounds present in host fruit, etc etc.
Authors’ reply: We have expanded the manuscript to better describe the integration of plant chemistry with protein modeling and molecular simulations. Line 92-101: “Molecular dynamics (MD) simulation techniques are widely used to predict the binding affinity, binding modes, and interaction mechanisms between

proteins and their ligands [28]. In olfactory research, molecular docking is commonly applied to identify binding sites and interaction modes between ORs and their specific volatile ligands, whereas MD simulations further reveal the structural stability and conformational dynamics of receptor-ligand complexes [29]. This method provides advantages in analyzing the three-dimensional structures, charge distributions, and interaction mechanisms of proteins (including ORs), offering new insights into the molecular basis of odorant recognition [27,29]. With the rapid advancement of computational technology, MD simulations have become increasingly efficient, serving as a powerful tool for elucidating protein functions and exploring OR-ligand recognition mechanisms [30]". Line 276-277: "To identify the host-derived volatile compounds of host fruit (Maoping Tangerine) for subsequent GC-MS analysis, volatile chemicals were collected using solid-phase microextraction (SPME)". Line 358-360: "To further evaluate the binding stability and conformational dynamics of odorant receptors (BtsuOr7a-6 and BtsuOr7a-4) complexed with their respective ligands (trans-nerolidol and piperitone), MD simulations were performed". Line 563-566: "Based on the SPME-GC–MS analysis and fluorescence binding assays, trans-nerolidol and piperitone were identified as the two major host-derived volatiles that showed strong binding affinity to BtsuOBP83a. To further elucidate the molecular recognition mechanisms underlying these interactions, molecular docking was performed using the predicted OBP structure".

Comment 4: Take a closer look at chromosome structure in Tephritid fruit flies. You report changes in chromosome number, but a lot of that is related to chromosome naming. Please attempt to say "autosomes", X and Y or if unknown be clear that the autosomes can't be separated from sex chromosome. One application of Illumina data can be to characterize the chromosome as a sex chromosome based on baised coverage in females vs males (e.g. AD ratio). This could be used to better assign your chromosomes as autosomes (there are 5) and X, rather than reporting as having 6 chromosomes (which is misleading). You also can review classical cytogenetic experiments as evidence.
Authors' reply: We have revised the Results section (Table 1) to clarify chromosome naming and to distinguish between autosomes and sex chromosomes. The revised manuscript notes that B. tsuneonis likely possesses five autosomes and one X chromosome, with Chr2 showing collinearity to the D. melanogaster X chromosome. Line 412-413: "Together, these results indicate that B. tsuneonis likely possesses a karyotype of five autosomes and one X chromosome (5 + X)". While Illumina read coverage–based sex chromosome assignment (AD ratio) was not performed in this study, we acknowledge that such analyses, along with classical cytogenetic evidence, will be valuable for future work.

In addition, we have carefully reviewed all comments and annotations provided in the marked-up PDF from Reviewer #3. Detailed responses have been inserted directly after each comment within the annotated PDF, ensuring that every point has been addressed clearly and specifically. We sincerely appreciate these constructive suggestions, which have further improved the accuracy and clarity of the revised manuscript.

Editorial Manager®

Cookie Preference Center

We use cookies which are necessary to make our site work. We may also use additional cookies to analyze, improve and personalize our content and your digital experience. For

more information, see our Cookie Policy.

You may choose not to allow some types of cookies. However, blocking some types may impact your experience of our site and the services we are able to offer. See the different category headings below to find out more or change your settings.

Allow all

Manage Consent Preferences

Strictly Necessary Cookies

Always active

These cookies are necessary for the website to function and cannot be switched off in our systems. They are usually only set in response to actions made by you which amount to a request for services, such as setting your privacy preferences, logging in or filling in forms. You can set your browser to block or alert you about these cookies, but some parts of the site will not then work.

Functional Cookies

These cookies enable the website to provide enhanced functionality and personalisation. They may be set by us or by third party providers whose services we have added to our pages. If you do not allow these cookies then some or all of these services may not function properly.

Performance Cookies

These cookies allow us to count visits and traffic sources so we can measure and improve the performance of our site. They help us to know which pages are the most and least popular and see how visitors move around the site.

Targeting Cookies

These cookies may be set through our site by our advertising partners. They may be used by those companies to build a profile of your interests and show you relevant adverts on other sites. If you do not allow these cookies, you will experience less targeted advertising.

Cookie List

Clear

Apply Cancel

Consent Leg.Interest

Confirm my choices

Powered by onetrust
