# Supplementary material for: A high-quality chromosome-level genome assembly of the oligophagous fruit fly Bactrocera tsuneonis (Diptera: Tephritidae) and insights into its host specificity
Source: Gigascience. 2025 Nov 20;14:giaf143. doi: 10.1093/gigascience/giaf143 (PMC12723664; doi:10.1093/gigascience/giaf143)
Supplement: giaf143_Reviewer_1_Report_Original_Submission — Jia-Ying Zhu -- 6/4/2025 [file giaf143_reviewer_1_report_original_submission.pdf]

Reviewer Report

Title: A high-quality chromosome-level genome assembly of the oligophagous fruit fly Bactrocera tsuneonis (Diptera: Tephritidae) and insights into its host specificity

Version: Original Submission **Date:** 6/4/2025

Reviewer name: Jia-Ying Zhu

Reviewer Comments to Author:

Guo et al. conducted genome sequencing of the citrus pest Bactrocera tsuneonis, assembling its genome into chromosome-level structures and performing comparative analysis after annotation. Through genomic analysis, OBP and OR genes were identified, and their differential expression in adult tissues, particularly in the antennae, was investigated. Key citrus volatiles were identified, and recombinant BtsuOBP83a and BtsuOBP83b were produced using the pGEM-T vector in Escherichia coli BL21. Fluorescence competitive binding assays demonstrated the strong binding affinity of BtsuOBP83a and BtsuOBP83b to two citrus volatiles, trans-nerolidol and piperitone. Molecular docking and dynamics simulations indicated that BtsuOr7a-6 and BtsuOr7a-4 may also bind these volatiles, suggesting their involvement in host odor recognition. The study provides insights into the olfactory adaptation and host specificity of B. tsuneonis towards citrus, aiding in the development of environmentally friendly pest control strategies. Several critical issues require attention:

1. Identification of specific active volatile compounds attracting B. tsuneonis to citrus, particularly through Y-tube olfactometer assays to confirm the attractiveness of trans-nerolidol and piperitone.
2. While molecular docking and dynamics simulations suggest the potential of BtsuOr7a-6 and BtsuOr7a-4 to bind trans-nerolidol and piperitone, additional experimental validation is essential to confirm their role as receptors for these volatiles.
3. Transcriptomic findings highlighting OBP and OR genes with heightened expression in the antennae necessitate validation through quantitative PCR to strengthen their association with olfactory processes in B. tsuneonis.

Methods

Are the methods appropriate to the aims of the study, are they well described, and are necessary controls included? Choose an item.

Conclusions

Are the conclusions adequately supported by the data shown? Choose an item.

Reporting Standards

Does the manuscript adhere to the journal's guidelines on minimum standards of reporting? Choose an item.

Choose an item.

Statistics

Are you able to assess all statistics in the manuscript, including the appropriateness of statistical tests used? Choose an item.

Quality of Written English

Please indicate the quality of language in the manuscript: Choose an item.

Declaration of Competing Interests

Please complete a declaration of competing interests, considering the following questions:

- Have you in the past five years received reimbursements, fees, funding, or salary from an organisation that may in any way gain or lose financially from the publication of this manuscript, either now or in the future?
- Do you hold any stocks or shares in an organisation that may in any way gain or lose financially from the publication of this manuscript, either now or in the future?
- Do you hold or are you currently applying for any patents relating to the content of the manuscript?
- Have you received reimbursements, fees, funding, or salary from an organization that holds or has applied for patents relating to the content of the manuscript?
- Do you have any other financial competing interests?
- Do you have any non-financial competing interests in relation to this paper?

If you can answer no to all of the above, write 'I declare that I have no competing interests' below. If your reply is yes to any, please give details below.

I declare that I have no competing interests.

I agree to the open peer review policy of the journal. I understand that my name will be included on my report to the authors and, if the manuscript is accepted for publication, my named report including any attachments I upload will be posted on the website along with the authors' responses. I agree for my report to be made available under an Open Access Creative Commons CC-BY license (http://creativecommons.org/licenses/by/4.0/). I understand that any comments which I do not wish to be included in my named report can be included as confidential comments to the editors, which will not be published.

Choose an item.

To further support our reviewers, we have joined with Publons, where you can gain additional credit to further highlight your hard work (see: https://publons.com/journal/530/gigascience). On publication of this paper, your review will be automatically added to Publons, you can then choose whether or not to claim your Publons credit. I understand this statement.

Yes Choose an item.
