# Supplementary material for: A high-quality chromosome-level genome assembly of the oligophagous fruit fly Bactrocera tsuneonis (Diptera: Tephritidae) and insights into its host specificity
Source: Gigascience. 2025 Nov 20;14:giaf143. doi: 10.1093/gigascience/giaf143 (PMC12723664; doi:10.1093/gigascience/giaf143)
Supplement: giaf143_Reviewer_2_Report_Original_Submission — Ioannis Ragoussis -- 6/25/2025 [file giaf143_reviewer_2_report_original_submission.pdf]

Reviewer Report

Title: A high-quality chromosome-level genome assembly of the oligophagous fruit fly Bactrocera tsuneonis (Diptera: Tephritidae) and insights into its host specificity

Version: Original Submission **Date:** 6/25/2025

Reviewer name: Ioannis Ragoussis

Reviewer Comments to Author:

The paper by Tengda Guo et al presents the assembly of the fruit fly Bactrocera tsuneonis genome, followed by evolutionary analysis of genes that may confer host specificity and the functional analysis of odorant receptors, leading to the identification of citrus volatiles that can be recognised by these receptors. These findings are in line with the specificity of this fly for citrus fruits and enhance our understanding of how these species evolved and adapted to specific hosts. Importantly, these findings open the way for suitable pest management strategies.

The genome assembly and transcriptomic work are convincingly executed. The assembly is at chromosome level and will enable further studies and comparisons to other tephritidae species by the wider biology and agriculture/pest management community.

The molecular work to characterise the odorant receptors and identify biding volatiles is very informative and also useful for the field.

Points:

1. The insects were collected from the wild. It is not indicated if one specimen was used for DNA extraction, or if more than one was used to extract DNA and DNA sequencing data. Also the sex of the specimen (s) was not indicated.
2. The same applies to the RNA and RNA-seq data, how many specimens were used for collecting tissues, what was the sex?

These are very important points, relevant for the use of the assembly and studies regarding polymorphisms and gene expression interpretation.

3. Figure 1: It is not clear as to whether chromosome y has no homology to any other chromosome of the species compared
4. A comparative analysis of the tephritidae sex chromosomes is not presented, in order to compare synteny levels to the one of autosomes.

Methods

Are the methods appropriate to the aims of the study, are they well described, and are necessary controls included? Choose an item.

Conclusions

Are the conclusions adequately supported by the data shown? Choose an item.

Reporting Standards

Does the manuscript adhere to the journal's guidelines on [minimum standards of reporting?]() Choose an item.

Choose an item.

Statistics

Are you able to assess all statistics in the manuscript, including the appropriateness of statistical tests used? Choose an item.

Quality of Written English

Please indicate the quality of language in the manuscript: Choose an item.

Declaration of Competing Interests

Please complete a declaration of competing interests, considering the following questions:

- Have you in the past five years received reimbursements, fees, funding, or salary from an organisation that may in any way gain or lose financially from the publication of this manuscript, either now or in the future?
- Do you hold any stocks or shares in an organisation that may in any way gain or lose financially from the publication of this manuscript, either now or in the future?
- Do you hold or are you currently applying for any patents relating to the content of the manuscript?
- Have you received reimbursements, fees, funding, or salary from an organization that holds or has applied for patents relating to the content of the manuscript?
- Do you have any other financial competing interests?
- Do you have any non-financial competing interests in relation to this paper?

If you can answer no to all of the above, write 'I declare that I have no competing interests' below. If your reply is yes to any, please give details below.

I declare that I have no competing interests

I agree to the open peer review policy of the journal. I understand that my name will be included on my report to the authors and, if the manuscript is accepted for publication, my named report including any attachments I upload will be posted on the website along with the authors' responses. I agree for my report to be made available under an Open Access Creative Commons CC-BY license (http://creativecommons.org/licenses/by/4.0/). I understand that any comments which I do not wish to be included in my named report can be included as confidential comments to the editors, which will not be published.

Choose an item.

To further support our reviewers, we have joined with Publons, where you can gain additional credit to further highlight your hard work (see: https://publons.com/journal/530/gigascience). On publication of this paper, your review will be automatically added to Publons, you can then choose whether or not to claim your Publons credit. I understand this statement.

Yes Choose an item.
