# Supplementary material for: A high-quality chromosome-level genome assembly of the oligophagous fruit fly Bactrocera tsuneonis (Diptera: Tephritidae) and insights into its host specificity
Source: Gigascience. 2025 Nov 20;14:giaf143. doi: 10.1093/gigascience/giaf143 (PMC12723664; doi:10.1093/gigascience/giaf143)
Supplement: giaf143_Reviewer_3_Report_Revision_1 — Scott M Geib -- 9/29/2025 [file giaf143_reviewer_3_report_revision_1.pdf]

Reviewer Report

Title: A high-quality chromosome-level genome assembly of the oligophagous fruit fly Bactrocera tsuneonis (Diptera: Tephritidae) and insights into its host specificity

Version: Revision 1 **Date:** 9/29/2025

Reviewer name: Scott M Geib

Reviewer Comments to Author:

Please find detailed edits in attached markup PDF.
Overall this is a rather comprehensive analysis of the Bactrocera genome and makes a nice contribution to the scientific community. I appreciate the focus on olfaction and the inclusion of protein structure analysis, modeling, and binding assays. A few comments:
1) This assembly is really PacBio + HiC, the Illumina data was not used for assembly. Illumina data was used in kmer based analysis, but the HiFi data alone could also have been used for this same analysis.
2) in the methods, full length transcriptome sequencing was mentioned (essentially IsoSeq), but it was unclear how it was analyzed and how it was used for overall genome annotation, manual annotation of genes (particularly can be useful for chemosensory genes) or tissue specific analyses. How was this data used?
3) I was able to follow, but really a better description of how plant chemistry is being used in combination of protein structure prediction, modeling, and protein expression needs to be made. Really walk people through how the structure of the insect proteins is being compared to the compounds present in host fruit, etc etc.
4) Take a closer look at chromosome structure in Tephritid fruit flies. You report changes in chromosome number, but a lot of that is related to chromosome naming. Please attempt to say "autosomes" , X and Y or if unknown be clear that the autosomes can't be separated from sex chromosome. One application of Illumina data can be to characterize the chromosome as a sex chromosome based on baised coverage in females vs males (e.g. AD ratio). This could be used to better assign your chromosomes as autosomes (there are 5) and X, rather than reporting as having 6 chromosomes (which is misleading). You also can review classical cytogenetic experiments as evidence.

Methods

Are the methods appropriate to the aims of the study, are they well described, and are necessary controls included? Choose an item.

Conclusions

Are the conclusions adequately supported by the data shown? Choose an item.

Reporting Standards

Does the manuscript adhere to the journal's guidelines on minimum standards of reporting? Choose an item.

Choose an item.

Statistics

Are you able to assess all statistics in the manuscript, including the appropriateness of statistical tests used? Choose an item.

Quality of Written English

Please indicate the quality of language in the manuscript: Choose an item.

Declaration of Competing Interests

Please complete a declaration of competing interests, considering the following questions:

- Have you in the past five years received reimbursements, fees, funding, or salary from an organisation that may in any way gain or lose financially from the publication of this manuscript, either now or in the future?
- Do you hold any stocks or shares in an organisation that may in any way gain or lose financially from the publication of this manuscript, either now or in the future?
- Do you hold or are you currently applying for any patents relating to the content of the manuscript?
- Have you received reimbursements, fees, funding, or salary from an organization that holds or has applied for patents relating to the content of the manuscript?
- Do you have any other financial competing interests?
- Do you have any non-financial competing interests in relation to this paper?

If you can answer no to all of the above, write 'I declare that I have no competing interests' below. If your reply is yes to any, please give details below.

I declare that I have no competing interests

I agree to the open peer review policy of the journal. I understand that my name will be included on my report to the authors and, if the manuscript is accepted for publication, my named report including any attachments I upload will be posted on the website along with the authors' responses. I agree for my report to be made available under an Open Access Creative Commons CC-BY license (http://creativecommons.org/licenses/by/4.0/). I understand that any comments which I do not wish to be included in my named report can be included as confidential comments to the editors, which will not be published.

Choose an item.

To further support our reviewers, we have joined with Publons, where you can gain additional credit to further highlight your hard work (see: https://publons.com/journal/530/gigascience). On publication of this paper, your review will be automatically added to Publons, you can then choose whether or not to claim your Publons credit. I understand this statement.

Yes Choose an item.
